# Chromosomal-level reference genome of Chinese peacock butterfly (*Papilio bianor*) based on third-generation DNA sequencing and Hi-C analysis

**DOI:** 10.1093/gigascience/giz128

**Published:** 2019-11-04

**Authors:** Sihan Lu, Jie Yang, Xuelei Dai, Feiang Xie, Jinwu He, Zhiwei Dong, Junlai Mao, Guichun Liu, Zhou Chang, Ruoping Zhao, Wenting Wan, Ru Zhang, Yuan Li, Wen Wang, Xueyan Li

**Affiliations:** 1 Center for Ecological and Environmental Sciences, Northwestern Polytechnical University, No.1 Dongxiang Road, Chang'an District, Xi'an, Shaanxi 710129, China; 2 State Key Laboratory of Genetic Resources and Evolution, Kunming Institute of Zoology, Chinese Academy of Sciences, No.32 Jiaochang Raod, Kunming, Yunnan 650223, China; 3 Key Laboratory of Animal Genetics, Breeding and Reproduction of Shaanxi Province, College of Animal Science and Technology, Northwest A&F University, No.22 Xinong Road,Yangling, Shaanxi 712100, China; 4 School of Marine Science and Technology, Zhejiang Ocean University, No.1 Haida South Road, Lincheng Changzhi Island, Zhoushan, Zhejiang 316022, China; 5 Nextomics Biosciences Institute, No.666 Gaoxin Road, Wuhan, Hubei 430000, China; 6 Center for Excellence in Animal Evolution and Genetics, Chinese Academy of Sciences, No.32 Jiaochang Raod, Kunming, Yunnan 650223, China

**Keywords:** *Papilio bianor*, single-molecule real-time (SMRT) sequencing, high-throughput chromosome conformation capture map, chromosome-level reference genome, butterfly

## Abstract

**Background:**

*Papilio bianor* Cramer, 1777 (commonly known as the Chinese peacock butterfly) (Insecta, Lepidoptera, Papilionidae) is a widely distributed swallowtail butterfly with a wide number of geographic populations ranging from the southeast of Russia to China, Japan, India, Vietnam, Myanmar, and Thailand. Its wing color consists of both pigmentary colored scales (black, reddish) and structural colored scales (iridescent blue or green dust). A high-quality reference genome of *P. bianor* is an important foundation for investigating iridescent color evolution, phylogeography, and the evolution of swallowtail butterflies.

**Findings:**

We obtained a chromosome-level *de novo* genome assembly of the highly heterozygous *P. bianor* using long Pacific Biosciences sequencing reads and high-throughput chromosome conformation capture technology. The final assembly is 421.52 Mb on 30 chromosomes (29 autosomes and 1 Z sex chromosome) with 13.12 Mb scaffold N50. In total, 15,375 protein-coding genes and 233.09 Mb of repetitive sequences were identified. Phylogenetic analyses indicated that *P. bianor* separated from a common ancestor of swallowtails ∼23.69–36.04 million years ago. Demographic history suggested that the population expansion of this species from the last interglacial period to the last glacial maximum possibly resulted from its decreased natural enemies and its adaptation to climate change during the glacial period.

**Conclusions:**

We present a high-quality chromosome-level reference genome of *P. bianor* using long-read single-molecule sequencing and Hi-C–based chromatin interaction maps. Our results lay the foundation for exploring the genetic basis of special biological features of *P. bianor* and also provide a useful data source for comparative genomics and phylogenomics among butterflies and moths.

## Background

Butterflies are widely considered one of the most aesthetically appealing and popular animals owing to their extraordinarily diverse wing patterns among species, populations, sexes, and seasonal forms [1–3]. They also have many other intriguing traits such as complex life cycles, diverse larval morphology and habits, and high species diversity [4]. In light of this interest, butterflies have been regarded as important model organisms in such fields as morphology, physiology, ecology, development, genetics, and evolutionary biology [4–6] since Darwin proposed his theory of natural selection in 1859 [7]. Back in 1864, Bates, the famous pioneer of mimicry theory, predicted that “the study of butterflies…will someday be valued as one of the most important branches of Biological science” [8]. With recent technological advances, it is possible to conduct direct analysis (and even manipulation) of the genomes of individuals sampled from natural habitats without the need of inbreeding to reduce heterozygosity or to develop laboratory lines [9–11]. Thus, butterflies are becoming a promising system to explore the genetics and evolution of morphological diversification and speciation.

Compared with the extensive butterfly diversity of >18,000 described species [12], only 37 butterfly species in 6 families including 5 swallowtails (Papilionidae) have had their reference genomes dissected (as of 1 May 2019) [9, 13–31]. Among them, chromosomal-level reference genomes have been assembled only for 2 nymphalids (*Heliconius melpomene* and *Melitaea cinxia*) and 1 swallowtail (*Papilio xuthus*) [9, 24, 25] using linkage map methods. Chromosomal-level reference genomes for more butterflies are not only indispensable to identify subtle genetic variations underpinning morphological traits that may often result from small mutations in regulatory elements [32, 33] but also will provide a unique opportunity to promote evolutionary biological studies on butterflies as an important model system.

The development of third-generation single-molecule technology has paved the way for the dissection of complex genomes of different kinds of wild organisms including butterflies [25, 28, 30, 34, 35]. Combined with high-throughput chromosome conformation capture (Hi-C) technology, which was developed to identify chromatin interactions across the entire genome and is now also used as a powerful tool to assist genome assembly [36], chromosomal-level reference genomes have been obtained for many organisms including such insects as fruit flies [37], mosquitoes [38], and moths [39, 40]. Despite this, up to now there have been no such examples combining single-molecule sequencing and Hi-C technologies to assemble chromosomal-level reference genomes reported for butterflies.

**Figure 1:**
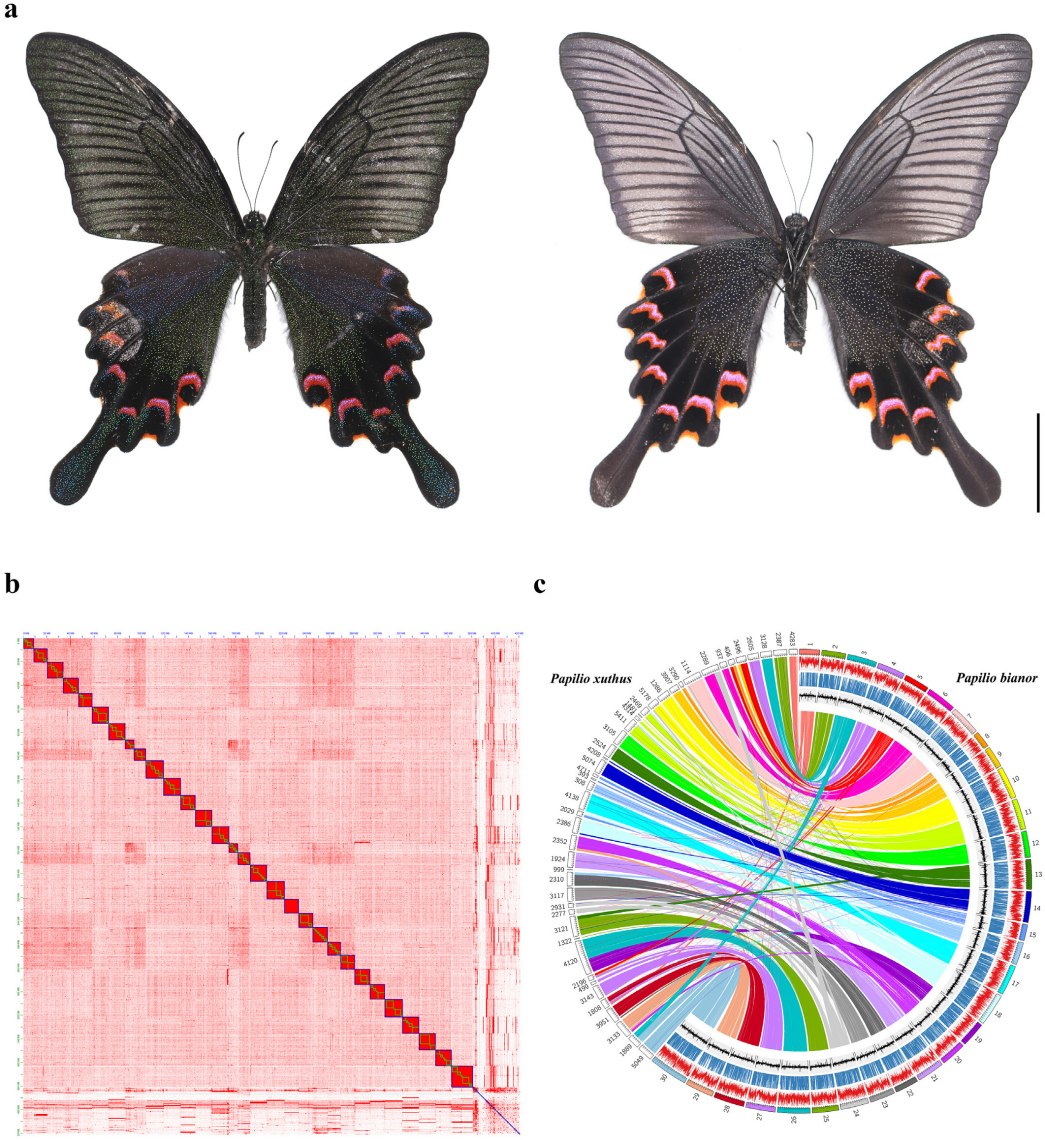
Characterization of *Papilio bianor*. (a) Female adult. Left, dorsal view; right, ventral view (scale bar = 20.0 mm; photo by Zhiwei Dong). (b) Heat map of chromosomal interactions. Each chromosome is framed with a blue block, and each scaffold is framed with a green block. (c) Circos plot of *P. bianor* chromosome-level reference genome with the previously released *Papilio xuthus* genome (obtained from a Chinese group) [9]. Shown from outermost to innermost are (1) gene density, (2) repeat element density, (3) GC content, and (4) syntenic regions with *P. xuthus* (left).

*Papilio bianor* Cramer, 1777 (NCBI:txid76199) (Papilionidae, Papilioninae, Papilionini) (Fig. 1a), also known as the Chinese peacock black swallowtail emerald or the Chinese peacock, is a widely distributed swallowtail butterfly with a large range of geographic populations ranging from the southeast of Russia to China, Japan, India, Vietnam, Myanmar, and Thailand [41–43]. Its larvae mainly feed on plants of the family Rutaceae, such as *Citrus reticulate, Euodia meliifolia*, and *Zanthoxylum bungeanum* [41, 44, 45], and its complete life cycle lasts 40–50 days. Its wing colors consist of both pigmentary colored scales (black, reddish) and structurally colored scales (iridescent blue or green dust) [45], which makes it a promising model to explore the origin and evolution of combined colors in insects. Scientific interest in *P. bianor* has long existed, e.g., in its prothoracicotropic hormones [46], oviposition behavior [44, 47, 48], phylogenetic position and species delimitation [49–53], chromosome numbers [54], or mitochondrial genome [50, 55]. Here, combining Pacific Biosciences (PacBio) single-molecule real-time (SMRT) and Hi-C technologies, we constructed the chromosome-level reference genome of *P. bianor* (30 chromosomes).

## Data Description

### Insect collection and breeding

Wild eggs of *P. bianor* were collected in a northern surburb of Kunming City, Yunnan, China and then reared under conditions of 26°C, 80% relative humidity with 16 h/8 h light/darkness. The hatched larvae were fed with the Rutaceous plant *Zanthxylum piperitum* under the same conditions. Two fifth instar larvae were collected for Hi-C sequencing. Pupae were reared under the same conditions as the eggs until their eclosion. Adults were collected for a genome survey using the IIlumina sequencing platform and for *de novo* genome sequencing using the PacBio platform.

### Genome survey using Illumina sequencing technology

Genomic DNA was isolated from the thorax and abdomen of a single male adult using a Gentra Puregene Blood kit (Qiagen, Germany) following manual instructions. Paired-end (PE) libraries of 2 different insertion sizes (150 and 500 bp) were constructed and sequenced on an Illumina HiSeq2000 platform at BGI (Shenzhen, China). The total number of sequencing reads was ∼16.45 Gb for PE150 and 28.42 Gb for PE500 (Table S1). We estimated genome size using Illumina short reads (PE150 and PE500), by *k-*mer distribution analysis with *k* = 17, using the formula: G = k-mer_number/k-mer_depth [56]. Our data indicate that *P. bianor* has an estimated genome size of 496.05 Mb and a high heterozygosity of 1.81% (Fig. S1 and Table S2).

### Library construction and sequencing using SMRT and Hi-C technologies

Genomic DNA was extracted from the thorax and abdomen of another male adult and used to construct one 20-kb library for the PacBio platform according to the manufacturers’ protocols (NextOmics, China). With 10 SMRT cells in the PacBio RSII platform, we generated 43.19 Gb subreads with a mean read length of 16.4 kb after removing adaptor sequences within sequences (Table S1). The long subreads were used fo*r de novo* genome assembly of *P. bianor*.

A sample mixed from the whole bodies of 2 male larval individuals (fifth instar) was used for library construction for Hi-C sequencing according to the methods reported in the previous study [36]. A 400–700 bp library was sequenced on the Illumina HiSeq X Ten platform with 150 PE mode, resulting in ∼75.11 Gb raw reads (Table S1).

### Chromosomal-level genome assembly

Considering the high heterozygosity of *P. bianor* (1.81%: Fig. S1 and Table S2), we first performed a PacBio-only assembly using Wtdbg (version 1.2.8; Wtdbg, RRID:SCR_017225; with –tidy-reads 5000 -k 0 -p 17 -S 1) [57], which is a *de novo* sequence assembler for noisy long reads produced by PacBio or Oxford Nanopore Technologies and is based on the fuzzy Bruijn graph algorithm. Second, to eliminate the high error rate of the PacBio long reads, we further polished the PacBio-only assembled sequences using Illumina reads as follows: all the Illumina reads were mapped to the PacBio-only assembly with BWA (version 0.7.12-r1039; BWA, RRID:SCR_010910) [58], which was further corrected with 2-round Pilon (version 1.21; Pilon, RRID:SCR_014731) correction [59, 60]. Third, because the polished assembly still contained a number of shorter contigs with significantly lower coverage, which perhaps represents the regions of high heterozygosity that were not merged to equivalent segments in the homologous chromosomes, we used a looser cut-off for identity (>90%) to merge the contigs with lower coverage and smaller size (size < 1,000 bp and coverage < 50 or size < 10,000 bp and coverage < 35) into the longer contigs as previously reported [14]. Fourth, the raw reads generated from the Hi-C sequencing were mapped to the polished assembled genome using Juicer (version 1.5; Juicer, RRID:SCR_017226) [61] and 3D *de novo* assembly (version 180114; 3D *de novo* assembly, RRID:SCR_017227) [38] to improve the assembly. Approximately 90.50% of contigs were anchored onto 30 super-scaffolds (Fig. 1b and Table S3; for more details see Fig. S2), which likely correspond to the 30 chromosomes as reported by cytogenetic karyotype [54]. Finally, we obtained the chromosomal-level high-quality assembly of *P. bianor* with a total length of ∼421.52 Mb and the longest scaffold N50 (13.12 Mb) of any published butterfly genome to date (Tables 1 and S4). The assembled genome accounts for 85% of the estimated genome size (496.05 Mb) by the *k-*mer distribution analysis (Table S2).

**Table 1:** Comparison of quality and composition of different butterfly genomes

Family	Species	Genome size (Mb)	Genome size without gap (Mb)	Heterozygosity^a^ (%)	Scaffold N50 (kb)	BUSCO^b^ (%)	*De novo* assembled transcripts^a^ (%)	GC content (%)	Repeat (%)	Exon (%)	Intron (%)	Number of proteins (k)
Papilionidae	*Papilio bianor*	421	421	1.8	13,120	96.3	NA	36.6	55.3	5.05	27.44	15.4
	*Papilio xuthus* [9]	244	238	1.0	6,199	97.6	NA	33.8	22.4	8.59	45.50	13.1
	*Papilio machaon* [9]	281	266	1.2	1,150	95.5	98	32.3	22.3	7.37	30.36	15.5
	*Papilio polytes*[17]	227	218	NA	3,672	91.8	NA	34.0	23.8	12.97	48.58	12.2
	*Papilio memnon*[23]	233	219	NA	5,457	96.6	NA	32.8	22.5	11.31	43.17	12.4
	*Papilio glaucus*[14]	375	361	2.3	231	95.5	98	35.4	22.0	5.07	25.60	15.7
Hesperiidae	*Achalarus lyciades*[31]	567	536	1.5	558	97.3	98	35.3	25.0	3.57	28.40	15.9
	*Lerema accius*[13]	298	290	1.5	525	95.1	98	34.4	15.5	6.96	31.60	17.4
	*Megathymus ursus violae* [16]	429	427	0.1	4,153	98.3	99	34.7	25.8	4.59	30.90	14.1
Pieridae	*Pieris rapae* [15]	246	243	1.5	617	98.0	99	32.7	22.7	7.91	33.30	13.2
	*Phoebis sennae* [21]	406	347	1.2	257	97.7	97	39.0	17.2	6.20	25.50	16.5
Nymphalidae	*Danaus plexippus* [18]	249	242	0.6	716	98.0	96	31.6	16.3	8.40	28.10	15.1
	*Heliconius melpomene* [24]	274	270	NA	194	95.6	NA	32.8	24.9	6.38	25.40	12.8
	*Melitaea cinxia* [25]	390	361	NA	119	83.0	97	32.6	27.5	4.34	31.20	16.7
	*Bicyclus anynana* [28]	475	470	NA	638	97.6	NA	36.5	25.8	4.73	38.36	22.6
Riodinidae	*Calephelis nemesis*[27]	809	783	0.5	206	95.6	99	34.9	34.8	2.25	19.60	15.4
	*Calephelis virginiensis* [27]	855	824	1.3	175	93.9	99	35.0	38.8	2.17	20.50	15.6
Lycaenidae	*Calycopis cecrops*[26]	729	689	1.2	233	95.5	96	37.1	34.0	3.11	24.00	16.5

^a^The heterozygosity of *P. bianor, P. machaon*, and *P. xuthus* was calculated on the basis of *k-*mer distribution analysis. The heterozygosity values of others (*P. glaucus, A. lyciades, L. accius, M. ursus violae, P. rapae, P. sennae, D. plexippus, C. nemesis, C. virginiensis, C. cecrops*) were estimated using the Genome Analysis Toolkit (GATK).

^b^BUSCO is calculated in this study.

NA: not available in the referenced citation.

### Quality evaluation of assembled genome

The assembled genome quality was evaluated using 3 methods. First, the completeness of the assembly was evaluated by BUSCO (version 2.0; BUSCO, RRID:SCR_015008) [62] with the insecta_odb9 BUSCO set. The BUSCO data showed that the *P. bianor* assembly covered 96.90% of the core genes with 96.30% covered genes complete (Table S5), which is similar to those of other published high-quality butterfly genomes (Table 1). We also checked the mapping rates of Illumina and PacBio reads to the *P. bianor* assembly by BWA (version 0.7.12-r1039; BWA, RRID:SCR_010910) [58] and BLASR (BLASR, RRID:SCR_000764) [63]. Our results indicate that 96.31% of Illumina reads mapped to the assembled genome with few heterozygous regions (Fig. S3 and Table S6); 96.86% of PacBio reads also mapped to the assembled genome with few heterozygous regions (Fig. S4 and Table S7). Third, we compared the syntenic relationships of the *P. bianor* genome with that of *P. xuthus*, which is the only chromosomal-level assembly (by linkage map methods) [9] among all *Papilio* reference genomes released to date and thus was considered to be the best-assembled one (Fig. 1c). We found that 61,082,412 bp of the *P. bianor* assembled genome could be aligned (1:1) with high confidence (-m 0.01) to the *P. xuthus* reference genome. All these results suggest that the *P. bianor* genome, which is assembled on the basis of PacBio reads, Illumina reads, and Hi-C data sequenced from different wild individuals, is of high quality (including completeness, base level contiguity, and accuracy) (Table 1).

### Genome annotation

**Figure 2:**
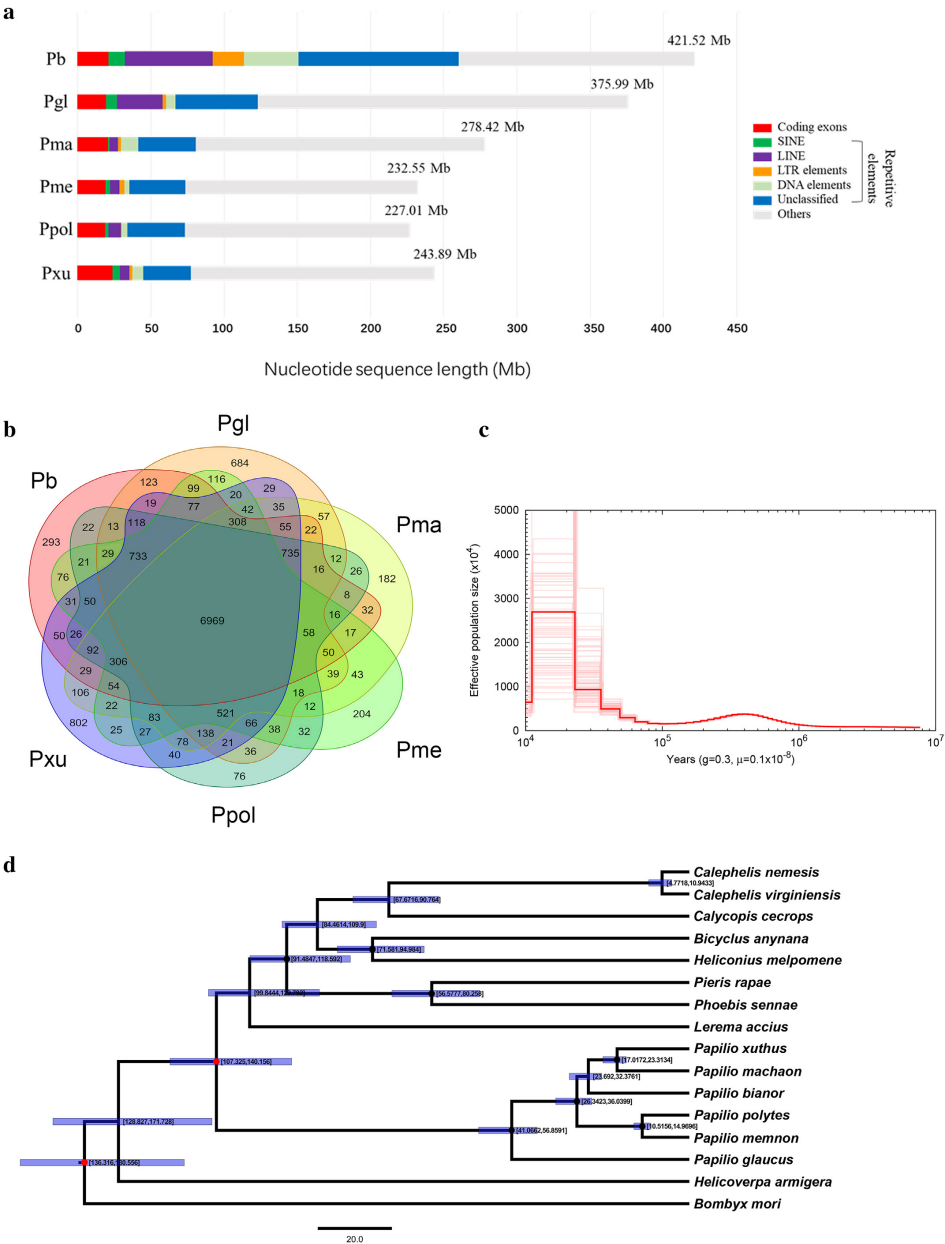
Genomic analysis of *Papilio bianor*. (a) Breakdown of the whole-genome assemblies into different functional classes in *Papilio*. (b) Venn diagram of the shared gene families of *Papilio*. (c) The dynamic changes of the effective population size were plotted using PSMC software, with 100 bootstrap replicates to test the robust variations. The parameter “g” represents the generation time in years, and the parameter “μ” means the per generation mutation rate. Pb: *Papilio bianor*; Pgl: *Papilio glaucus*; Pma: *Papilio machaon*; Pme: *Papilio memnon*; Ppol: *Papilio polytes*; Pxu: *Papilio xuthus*. (d) Maximum likelihood phylogenetic tree of Papilionoidea constructed by the concatenated alignment of 1,378 1-to-1 single-copy ortholog genes. The numbers in the square brackets on the nodes are the 95% confidence intervals of divergence time. The red dots are fossil evidence downloaded from the TimeTree website [89], and the black dots are inferred time obtained from the TimeTree website. Both were used to calibrate divergent time.

Repetitive sequences including tandem repeats and transposable elements (TEs) were searched for in the *P. bianor* assembled genome. To do this, we first used Tandem Repeats Finder (version 4.07b; Tandem Repeats Database, RRID:SCR_005659; with 2 7 7 80 10 50 2000 -d -h parameters) [64] to annotate the tandem repeats. Then, TEs were identified using a combination of *de novo* and homology-based approaches at both the DNA and protein levels. At the DNA level, we used RepeatModeler (version 1.0.4; RepeatModeler, RRID:SCR_015027) [65] to construct a *de novo* repeat library, which built a repeat consensus database with classification information, and then we adopted RepeatMasker (version 4.0.5; RepeatMasker, RRID:SCR_012954) [66] to search similar TEs against the known Repbase TE library (version 16.02) [67] and *de novo* repeat library. We also used LTR_FINDER (LTR Finder, RRID:SCR_015247) [68] to find long terminal repeats (LTRs). At the protein level, software RepeatProteinMask (version 3.3.0, a package in RepeatMasker) [66] was used to search the assembled *P. bianor* genome against the TE protein database using the WU-BLASTX engine. Finally, we identified and masked 55.3% of the *P. bianor* assembly as repeat regions (Table S8), which is the highest in published butterfly genomes (Table 1). Among all TEs, the most abundant class of repetitive elements are long interspersed nuclear elements (LINEs, 14.22%), and the next are DNA transposons (8.81%) (Table S9). Compared with the reference genomes of other swallowtail butterflies, LINEs, DNA transposons, and LTRs have expanded in the *P. bianor* genome (Fig. 2a). To confirm the reliability of the high repetitive sequences seen in *P. bianor*, which is much higher than those (40%) of other butterflies (Table 1), we also used other *de novo* annotation methods reported by Lavoie et al. [69] and Platt II et al. [70] to annotate the repetitive sequences of the *P. bianor* genome. On the basis of these methods, the *P. bianor* genome possesses 53% repeat elements, similar to the previous annotated results (Tables 1 and S8), thus confirming a high proportion of repetitive sequences in the *P. bianor* genome.

To annotate protein-coding genes of *P. bianor*, we used both *de novo* and homology-based gene prediction approaches. For *de novo* gene prediction, the repeat-masked genome was analyzed by SNAP (version 2006–07-28; SNAP, RRID:SCR_002127) [71], GENSCAN (version 1.0; GENSCAN, RRID:SCR_012902) [72], glimmerHMM (version 3.0.3; glimmerHMM, RRID:SCR_002654) [73], and AUGUSTUS (version 2.5.5; Augustus, RRID:SCR_008417) [74]. For homology-based predictions, the protein sequences from 8 insect species including the beetle *Tribolium castaneum* [75], fruit fly *Drosophila melanogaster* [76], silkworm *Bombyx mori* [77], moth *Helicoverpa armigera* [78], and 4 butterfly species *Papilio polytes* [23], *P. xuthus* [9], *Heliconius melpomene* [24], and *Danaus plexippus* [20] were used as templates for homology-based gene prediction. Then we used TBLASTN (version 2.2.26; TBLASTN, RRID:SCR_011822) [79] with an E-value cut-off of 1e−5 to align the protein sequences of the reference gene set to the *P. bianor* genome, and GeneWise (v2.2.0; GeneWise, RRID:SCR_015054) [80] to perform more precise alignment. Gene sequences with length < 150 bp or percent identity < 25% were removed. EvidenceModeler software (EVM, version 1.1.1; RRID:SCR_014659) [81] was used to integrate the genes predicted by the homology and *de novo* approaches and generate a comprehensive, non-redundant gene set. Finally, 15,375 protein-coding genes were annotated in the assembled *P. bianor* genome (Table S10), which is similar to the published reference genomes of other swallowtail butterflies (Fig. S3).

The KEGG, TrEMBL, SwissProt, and COG databases were searched for best matches to *P. bianor* for the protein sequences yielded by EVM software, using BLASTP (version 2.2.26; BLASTP, RRID:SCR_001010) with an E-value cutoff of 1e−5, and Pfam, PRINTS, ProDom, and SMART databases were searched for known motifs and domains in our sequences using InterProScan software (version 5.18–57.0; InterProScan, RRID:SCR_005829) [82]. We also searched all predicted gene sequences against the GenBank nonredundant protein (nr) database using BLASTN (BLASTN, RRID:SCR_001598) with a maximal e-value of 1e−5. In sum, 13,343 genes were annotated with ≥1 related function, which accounts for ∼86.78% of the *P. bianor* annotated genes (Table S11).

### Gene family identification and phylogenetic analysis

We used OrthoMCL (version 2.0.9; OrthoMCL DB: Ortholog Groups of Protein Sequences, RRID:SCR_007839) [83] to cluster the *P. bianor* annotated genes with an E-value cutoff of 1 e−5, and Markov Chain Clustering with default inflation parameter in an all-to-all BLASTP analysis of entries for the reference genomes of 6 swallowtail butterflies including *P. bianor* in this study and the other 5 published so far (*P. polytes, P. xuthus, Papilio machaon, Papilio glaucus*, and *Papilio memnon*). The result showed that 293 gene families were specific to *P. bianor* (Fig. 2b). Using CAFE, version 4.0.1 [84], we also identified 375 expanded gene families and 1,863 contracted gene families in *P. bianor*. The *P. bianor* expanded gene families were enriched in 17 GO categories and the contracted gene families were enriched in 14 GO categories, most of which are related to oxygen metabolism (Tables S12 and S13).

To reveal the phylogenetic position of *P. bianor* among Papilionoidea, we selected 14 butterfly species in 5 families (Papilionidae [6]: *P. xuthus, P. polytes, P. machaon, P. glaucus, P. memnon*; Hesperiidae [1]: *Lerema accius*; Pieridae [2]: *Phoebis sennae, Pieris rapae*; Nymphalidae [2]: *Bicyclus anynana, Heliconius melpomene*; Riodinidae [2]: *Calephelis nemesis, Calephelis virginiensis*; Lycaenidae [1]: *Calycopis cecrops*) [9, 13–15, 17, 21, 23, 24, 26–28] with 2 moths (*B. mori* [77], *H. armigera* [78]) as outgroups for phylogenetic analysis. A total of 1,378 one-to-one single-copy orthologs that contain only 1 protein for each species were collected and clustered by OrthoMCL (version 2.0.9; OrthoMCL DB: Ortholog Groups of Protein Sequences, RRID:SCR_007839) [83] from these 16 species and their nucleic acid sequences were aligned using PRANK (version 3.8.31; PRANK, RRID:SCR_017228) [85]. Gene alignments were concatenated and phylogenetic trees were constructed using RAxML (version 7.2.8; RAxML, RRID:SCR_006086) [86] with the GTR+G+I model. Furthermore, to clarify our results, we also have constructed the gene trees for each of the orthologs with RAxML software (version 7.2.8; RAxML, RRID:SCR_006086) [84] by choosing the GTR+G+I model and inferred the species tree from these with ASTRAL software (version 5.6.3) [87] (Fig. S4). As expected, the results are consistent with each other. To further investigate the divergence time of these species, the phylogeny was further analyzed by MCMCtree in PAML (version 4.5; PAML, RRID:SCR_014932) software [88] using default parameters, and calibrated with published divergent times of some nodes estimated from fossil evidence or obtained from the TimeTree website [89]. Our phylogenetic tree showed that *P. bianor* clusters at the base of *P. machaon* and *P. xuthus* and diverged from them 23 million years ago (mya); the *Papilio* genus was monophyletic with a crown node age of ∼41.07–56.86 mya (Fig. 2d). This tree is largely consistent with those constructed from cytochrome oxidases I, cytochrome oxidases II, and elongation factor 1α [90, 91], and from 425 loci from 2 outgroups and 173 species of butterflies [92].

We also inferred the demographic histories of *P. bianor* applying the Pairwise Sequentially Markovian Coalescence (PSMC; PSMC, RRID:SCR_017229; with -p 64*1 parameters) analysis [93] (3.56 × 10^−3^ mutations per site per generation calculated by r8s [94]; 3 or 4 generations per year [48]), which was carried out by mapping Illumina short reads to the assembled genome with BWA (version 0.7.12-r1039; BWA, RRID:SCR_010910) [58] and calling variants with SAMtools (version 1.3.1; SAMTOOLS, RRID:SCR_002105; with samtools mepileup -C50 –uf parameters) [95]. Our result suggested that the effective population size increased significantly corresponding to the transition phase from the last interglacial period (∼0.14–0.12 mya) to the last glacial maximum (∼0.021–0.018 mya) (Fig. 2c), which is in good agreement with the other 5 published *Papilio* species [96]. We hypothesize that the population expansion of this species possibly results from the decrease of its natural enemies (e.g., birds or lizards) and from its adaptation to climate change during the last interglacial period and last glacial maximum

## Conclusion

We present the chromosomal-level genome assembly of *P. bianor* with a contig and scaffold N50 of 5.50 and 12.51 Mb, respectively. The assembled genome included 15,375 protein-coding genes, 293 species-specific gene families, 375 expanded gene families, and 1,863 contracted gene families. *P. bianor* diverged from other *Papilio* ∼23.69–36.04 mya. Our results also show that the effective population size of *P. bianor* increased significantly during the glacial period. Our results lay the foundation for exploring the special biological features of the Chinese peacock butterfly, and also provide a useful data source for comparative genomics and phylogenomics among butterflies and lepidopterans.

## Availability of Supporting Data and Materials

The raw reads have been deposited at NCBI in the SRA under BioProject Number: PRJNA530186. The chromosome-level genome, annotation, and other supporting data are also available via the *GigaScience* database, GigaDB [97].

## Additional Files

**Figure S1:** *k-mer* (*k* = 17) distribution in *Papilio bianor* genome. The first peak (depth = 26) is a heterozygous peak, which is higher than the main peak (depth = 53), suggesting that the *P. bianor* genome is highly heterozygous. The x-axis is depth (×); the y-axis is the proportion that represents the frequency at that depth divided by the total frequency of all the depth.

**Figure S2:** Heat map of per-chromosomal interactions. Each scaffold is framed with a green block.

**Figure S3:** The coverage distribution of Illumina reads mapping to *Papilio bianor* genome. The histogram follows a normal distribution, indicating few heterozygous regions in the assembled genome.

**Figure S4:** The coverage distribution of PacBio reads mapping to *Papilio bianor* genome. The histogram follows a normal distribution, indicating few heterozygous regions in the assembled genome.

**Figure S5:** The statistics of annotated protein-coding genes of *Papilio*. (a) Messenger RNA length, (b) coding sequence (CDS) length, (c) exon length, (d) intron length, (e) exon number. The x-axis represents length or number and the y-axis represents the density of genes. Pb: *Papilio bianor*; Pgl: *Papilio glaucus*; Pma: *Papilio machaon*; Pme: *Papilio memnon*; Ppol: *Papilio polytes*; Pxu: *Papilio xuthus*.

**Figure S6:** Maximum Likelihood phylogenetic tree of Papilionoidea constructed by merging each of the single-copy orthologs.

**Table S1:** The statistics of sequencing data generated for *Papilio bianor* genome. The sequencing depth was calculated by the assembled genome size.

**Table S2:** Genome size estimation of *Papilio bianor* with *k-*mer distribution analysis using *k* = 17.

**Table S3:** The statistics of assembled chromosome-level genome of *Papilio bianor*. The Hi-C data were filtered by HiC-Pro software. In total, 6,690,421 pairs of reads, accounting for 68.04% of the total Hi-C data, were used in downstream analysis.

**Table S4:** The contiguity assessment of genome assembly of *Papilio bianor*.

**Table S5:** The quality evaluation of assembled genome of *Papilio bianor* by BUSCO software with insecta_odb9.

**Table S6:** The statistics of mapping ratio of Illumina reads to *Papilio bianor* assembled genome.

**Table S7:** The statistics of mapping ratio of PacBio reads to *Papilio bianor* assembled genome.

**Table S8:** The statistics of the annotated repeat sequences in *Papilio bianor* genome.

**Table S9:** The statistics of the TE contents in *Papilio bianor* genome.

**Table S10:** The statistics of predicted protein-coding genes in *Papilio bianor* genome.

**Table S11:** The statistics of gene function annotation in *Papilio bianor* genome.

**Table S12:** The GO term enrichment of expanded gene families in *Papilio bianor* genome.

**Table S13:** The GO term enrichment of contracted gene families in *Papilio bianor* genome.

giz128_GIGA-D-19-00120_Original_Submission

giz128_GIGA-D-19-00120_Revision_1

giz128_Response_to_Reviewer_Comments_Original_Submission

giz128_Reviewer_1_Report_Original_Submission Christopher Ward -- 5/14/2019 Reviewed

giz128_Reviewer_1_Report_Revision_1 Christopher Ward -- 9/3/2019 Reviewed

giz128_Reviewer_2_Report_Original_Submission Annabel Charlotte Whibley -- 5/19/2019 Reviewed

giz128_Supplemental_Files

## Abbreviations

BLASR: Basic Local Alignment with Successive Refinement; bp: base pair; BUSCO: Benchmarking Universal Single-Copy Orthologs; BWA: Burrows-Wheeler Aligner; CAFE: Computational Analysis of gene Family Evolution; COG: Clusters of Orthologous Groups; EVM: EvidenceModeler; Gb: gigabase pairs; GC: guanine-cytosine; GO: gene ontology; Hi-C: high-throughput chromosome conformation capture; kb: kilobase pairs; KEGG: Kyoto Encyclopedia of Genes and Genomes; LINE: long interspersed nuclear element; LTR: long terminal repeat; Mb: megabase pairs; mya: million years ago; NCBI: National Center for Biotechnology Information; PacBio: Pacific Biosciences; PAML: Phylogenetic Analysis by Maximum Likelihood; PE: paired-end; PSMC: Pairwise Sequentially Markovian Coalescence; RAxML: Randomized Axelerated Maximum Likelihood; SMRT: single-molecule real-time; SNAP: Semi-HMM-based Nucleic Acid Parser; SRA: Sequence Read Archive; TE: transposable element; TrEMBL: Translation of European Molecular Biology Laboratory.

## Competing interests

The authors declare that they have no competing interests.

## Authors' Contributions

X.L. and W.Wang conceived and supervised the study. J.H., Z.D., Z.C., G.L., R.Zhao, and W.Wan fed and collected the samples. G.L., J.H., R.Zhao, and W.Wan extracted the genomic DNA. Y.L. took charge of Hi-C sequencing. S.L. and X.D. assembled the genome. S.L., J.Y., F.X., and R.Zhang carried out the quality assessment, repeat annotation, and gene annotation. J.Y., F.X., J.M., and R.Zhang carried out evolutionary analyses. S.L. uploaded the raw read data, genome assembly, and annotation in the GenBank and *GigaScience* (GigaDB) databases. S.L., X.L., and W.Wang wrote the manuscript. All authors read and approved the final manuscript.
