# Supplementary material for: Chromosomal-level reference genome of Chinese peacock butterfly (Papilio bianor) based on third-generation DNA sequencing and Hi-C analysis
Source: Gigascience. 2019 Nov 4;8(11):giz128. doi: 10.1093/gigascience/giz128 (PMC6827417; doi:10.1093/gigascience/giz128)
Supplement: giz128_GIGA-D-19-00120_Original_Submission [file giz128_giga-d-19-00120_original_submission.pdf]

GigaScience

Chromosomal-level reference genome of Chinese peacock butterfly (Papilio bianor) based on third-generation DNA sequencing and Hi-C analysis

--Manuscript Draft--

**Manuscript Number:**	GIGA-D-19-00120
**Full Title:**	Chromosomal-level reference genome of Chinese peacock butterfly (Papilio bianor) based on third-generation DNA sequencing and Hi-C analysis
**Article Type:**	Data Note
**Funding Information:**	National Natural Science Foundation of China (31621062) — Dr. Wen Wang<br>Chinese Academy of Sciences (XDB13000000) — Dr. Wen Wang<br>CAS "Light of West China" — Dr. Xueyan Li
**Abstract:**	Background<br><br>Papilio bianor Cramer, 1777 (i.e. Chinese peacock) (Insecta, Lepidoptera, Papilionidae) is a widely distributed swallowtail butterfly with a large number of geographic populations from the Southeast of Russia to China, Japan, India, Vietnam, Myanmar and Thailand. Its wing color consists of both pigmentary colored scales (black, reddish) and structural colored scales (iridescent blue or green dust). A high-quality reference genome of P. bianor is thus important for investigating iridescent color evolution, phylogeography, and evolution of swallowtail butterflies.<br><br>Findings<br><br>Here, we obtained a chromosome-level de novo genome assembly of the high heterozygous Chinese peacock (Papilio bianor) (1.81%) using long Pacific Biosciences (PacBio) sequencing reads (43.19 Gb) and high-through chromosome conformation capture (Hi-C) technology. The final assembly is 402.00 Mb on 30 chromosomes (29 autosomes and 1 sex chromosomes W) with 5.50 Mb contig N50 and 12.51 Mb scaffold N50. Totally 15,375 protein-coding genes and 222.29 Mb (55.30%) of repetitive sequences were identified. The phylogenetic trees of representative species of butterflies and moths constructed using one to one single-copy orthologous genes indicate that the Chinese peacock was separated from a common ancestor of swallowtails about 23.69-36.04 million years ago (mya). Demographic history inferred using the Pairwise Sequentially Markovian Coalescence (PSMC) analysis suggested that the population expansion of this species from the last interglacial period to the last glacial maximum possibly resulted from its decreased natural enemies and its adaptation to climate diversity during glacial period.<br><br>Conclusions<br><br>We present a high-quality chromosome-level reference genome of the Chinese peacock (Papilio bianor) using long-read single-molecule sequencing and Hi-C-based chromatin interaction maps. Our results lay the foundation for exploring genetic basis of special biological features of the Chinese peacock butterfly, and also provide a useful datasource for comparative genomics and phylogenomics among butterflies and moths.
**Corresponding Author:**	Xueyan Li, Ph.D<br><br>CHINA
**Corresponding Author Secondary Information:**	
**Corresponding Author's Institution:**	
**Corresponding Author's Secondary**	

Powered by Editorial Manager® and ProduXion Manager® from Aries Systems Corporation

**Institution:**	
**First Author:**	Xueyan Li, Ph.D
**First Author Secondary Information:**	
**Order of Authors:**	Xueyan Li, Ph.D
	Sihan Lu, Ph.D
	Jie Yang
	Xuelei Dai
	Feiang Xie
	Jinwu He
	Zhiwei Dong
	Junlai Mao
	Guichun Liu
	Zhou Chang
	Ruoping Zhao
	Wenting Wan
	Ru Zhang
	Wen Wang
**Order of Authors Secondary Information:**	
**Additional Information:**	
**Question**	**Response**
Are you submitting this manuscript to a special series or article collection?	No
**Experimental design and statistics**<br><br>Full details of the experimental design and statistical methods used should be given in the Methods section, as detailed in our Minimum Standards Reporting Checklist. Information essential to interpreting the data presented should be made available in the figure legends.<br><br>Have you included all the information requested in your manuscript?	Yes
**Resources**<br><br>A description of all resources used, including antibodies, cell lines, animals and software tools, with enough information to allow them to be uniquely identified, should be included in the	Yes

Powered by Editorial Manager® and ProduXion Manager® from Aries Systems Corporation

Methods section. Authors are strongly encouraged to cite Research Resource Identifiers (RRIDs) for antibodies, model organisms and tools, where possible.<br><br>Have you included the information requested as detailed in our Minimum Standards Reporting Checklist?	
**Availability of data and materials**<br><br>All datasets and code on which the conclusions of the paper rely must be either included in your submission or deposited in publicly available repositories (where available and ethically appropriate), referencing such data using a unique identifier in the references and in the “Availability of Data and Materials” section of your manuscript.<br><br>Have you have met the above requirement as detailed in our Minimum Standards Reporting Checklist?	Yes

Manuscript

Click here to download Manuscript Pb-Genome_submitted version_MS_20190411.docx

Chromosomal-level reference genome of Chinese peacock butterfly (*Papilio bianor*) based on third-generation DNA sequencing and Hi-C analysis

Sihan Lu[1,2,†], Jie Yang[1,†], Xuelei Dai[3,†], Feiang Xie[4,†], Jinwu He[1], Zhiwei Dong[2], Junlai Mao[4], Guichun Liu[1,2], Zhou Chang[2], Ruoping Zhao[2], Wenting Wan[1], Ru Zhang[1], Wen Wang[2,5,*,#], Xueyan Li[2,*]

[1] Center for Ecological and Environmental Sciences, Northwestern Polytechnical University, Xi'an, Shanxi 710072, China.

[2] State Key Laboratory of Genetic Resources and Evolution, Kunming Institute of Zoology, Chinese Academy of Sciences, Kunming, Yunnan 650223, China.

[3] Key Laboratory of Animal Genetics, Breeding and Reproduction of Shaanxi Province, College of Animal Science and Technology, Northwest A&F University, Yangling 712100, China

[4] School of Marine Science and Technology, Zhejiang Ocean University, Zhoushan, Zhejiang 316022, China

[5] Center for Excellence in Animal Evolution and Genetics, Kunming, Yunnan 650223, China

[†]These authors contributed equally to this work.

[*]Correspondence should be addressed to L.X.Y (lixy@mail.kiz.ac.cn), W.W (wwang@mail.kiz.ac.cn).

[#]Current address: Center for Ecological and Environmental Sciences, Northwestern Polytechnical University, Xi'an, Shanxi 710072, China

Abstract

Background: *Papilio bianor* Cramer, 1777 (i.e. Chinese peacock) (Insecta, Lepidoptera, Papilionidae) is a widely distributed swallowtail butterfly with a large number of geographic populations from the Southeast of Russia to China, Japan, India, Vietnam, Myanmar and Thailand. Its wing color consists of both pigmentary colored scales (black, reddish) and structural colored scales (iridescent blue or green dust). A high-quality reference genome of *P. bianor* is thus important for investigating iridescent color evolution, phylogeography, and evolution of swallowtail butterflies.

Findings: We obtained a chromosome-level *de novo* genome assembly of the high heterozygous *Papilio bianor* (1.81 %) using long Pacific Biosciences (PacBio) sequencing reads and high-through chromosome conformation capture (Hi-C) technology. The final assembly is 402.00 Mb on 30 chromosomes (29 autosomes and 1 sex chromosomes W) with 12.51 Mb scaffold N50. Totally 15,375 protein-coding genes and 222.29 Mb of repetitive sequences were identified. The phylogenetic trees indicated that *P. bianor* was separated from a common ancestor of swallowtails about 23.69-36.04 million years ago. Demographic history suggested that the population expansion of this species from the last interglacial period to the last glacial maximum possibly resulted from its decreased natural enemies and its adaptation to climate diversity during glacial period.

Conclusions: We present a high-quality chromosome-level reference genome of *Papilio bianor* using long-read single-molecule sequencing and Hi-C-based chromatin interaction maps. Our results lay the foundation for exploring genetic basis of special biological features of *P. bianor*, and also provide a useful datasource for comparative genomics and phylogenomics among butterflies and moths.

Keywords: *Papilio bianor*; single-molecule real-time (SMRT) sequencing; High-through chromosome conformation capture (Hi-C) map; chromosome-level reference genome; Butterfly.

Background information

Butterflies are one of most charming animals especially for their extraordinarily diverse wing patterns among species, populations, sexes, and even seasonal forms [1-3]. They also have many other intriguing traits such as complex life cycles, diverse larval morphology and habits, and high species diversity etc. [4]. Thus, butterflies have been regarded as one of the most important model organisms in different fields from morphology, physiology, ecology, development, genetics to evolutionary biology [4-6] since Darwin proposed his theory of natural selection in 1859 [7]. Back in 1864, Bates, the famous inventor of mimicry theory, predicted that *"the study of butterflies…will someday be valued as one of the most important branches of Biological science."* [8]. With the feasibility to dissect the heterozygous genomes of such wild insects like butterflies and to perform genetic manipulation on them [9-11], butterflies have been becoming a promising system to explore the genetics, evolution, morphological diversification and speciation.

Compared with butterfly diversity of more than 18,000 described species [12], only 37 butterfly species in 6 families including five swallowtails (Papilionidae) have their reference genomes dissected [9, 13-30]. Among them, the chromosomal-level reference genomes were assembled only for two nymphids (*Heliconius melpomene* and *Melitaea cinxia*) and one swallowtail (*Papilio xuthus*) [9, 24, 25] using linkage map method. Chromosomal-level reference genomes for more butterflies are not only indispensible to identify subtle genetic variations underpinning morphological traits which often resulted from small mutations in regulatory elements [31, 32], but also will provide unique opportunity to promote the evolutionary biological studies on the famous butterfly system.

The development of third generation single molecule technology has paved the way to dissect complex genomes of different kinds of wild organisms including butterflies [25, 28, 30, 33, 34]. Combined with high-through chromosome conformation capture (Hi-C) technology, which is mainly used to identify chromatin interactions across the entire genome and now also used as a powerful tool to assist genome assembly [35], chromosomal-level reference genomes have been obtained for some organisms including such insects as fruit fly [36], mosquito [37], moth [38, 39] etc. Nevertheless, up to now no such case combining single molecule sequencing and Hi-C technologies to assemble chromosomal-level reference genomes is reported for butterflies.

Papilio bianor Cramer, 1777 (Papilionidae, Papilioninae, Papilionini) (**Fig. 1a**), also known as Chinese peacock black swallowtail emerald or Chinese peacock, is a widely distributed swallowtail butterfly with a large range of geographic populations from the Southeast of Russia to China, Japan, India, Vietnam, Myanmar and Thailand [40-42]. Its larvae mainly feed plants of Rutaceae like *Citrus reticulate*, *Euodia meliifolia* and *Zanthoxylum bungeanum* [40, 43, 44], and its complete life cycle spend 40 to 50 days. Its wing colors consist of both pigmentary colored scales (black, reddish) and structural colored scales (iridescent blue or green dust) [44], which make it a promising model to explore the origin and evolution of combined colors in insects. Scientific interests in *P. bianor* have long existed, for examples in its prothracicotropic hormones (PTTHs) [45], oviposition behavior [43, 46, 47], phylogenetic position and species delimit [48-52], chromosome numbers [53] or mitochondrial genome [49, 54]. Here, combining SMRT and Hi-C technologies, we

constructed the chromosome-level reference genome of *P. bianor* (30 chromosomes), which is the fourth chromosomal-level reference genome in butterflies.

Data Description

Insect collection and breeding

Wild eggs of *P. bianor* were collected in north surburb of Kunming city (Yunnan, China), and then reared under the conditions of 26 ℃, 80% relative humidity with 16h/8h light/darkness. The hatched larvae were fed with Rutaceous plant *Zanthxylum piperitum* under the same conditions. Two 5th instar larva were collected for Hi-C sequencing. Pupa were reared under the same conditions as the eggs until their eclosion. Adults were collected for genome survey using IIlumina platform and for *de novo* genome sequencing using PacBio platform.

Genome survey using Illumina sequencing technology

Genomic DNA was isolated from thorax and abdomen of single male adult using a Gentra Puregene Blood kit (Qiagen, Germany) following manual instructions. Paired-end libraries of two different insertion sizes (150 bp and 500 bp) were constructed and sequenced on an Illumina HiSeq2000 platform at BGI (Shenzhen, China). The total number of sequencing reads was approximately 16.45 Gb for PE150 and 28.42 Gb for PE500 (**Table S1**). We estimated genome size using Illumina short reads (PE150 and PE500), by k-mer distribution analysis with k = 17, using the formula: G = k-mer_number/k-mer_depth [55]. Our data indicate that *P. bianor* has an estimated genome size of 473.07 Mb and a high heterozygosity of 1.81% (**Fig. S1** & **Table S2**).

Library construction and sequencing using SMRT and Hi-C technologies

Genomic DNA was extracted from thorax and abdomen of another male adult and used to construct one 20-kb library for the PacBio platform according to the manufacturers' protocols (NextOmics, China). With ten single-molecular real-time (SMRT) cells in the PacBio RSII platform, we generated 43.19 Gb subreads with a average read length of 16.4 kb after removing adaptor sequences within sequences (**Table S1**). The long subreads were used for *de novo* genome assembly of *P. bianor*.

The sample mixed from whole body of two male larval individuals (the fifth instar) was used to construct library for Hi-C sequencing according to the similar method in the previous study [35]. A 400-700 bp library was sequenced on the Illumina HiSeq X Ten platform with 150 paired-end mode, and resulted in ~75.11 Gb raw reads (**Table S1**).

Chromosomal-level genome assembly

Considering the high heterozygosity of *P. bianor* (1.81%: **Fig. S1** & **Table S2**), we firstly performed a PacBio-only assembly using Wtdbg (v1.2.8; with --tidy-reads 5000 -k 0 -p 17 -S 1) [56], which is a *de novo* sequence assembler for long noisy reads produced by PacBio or Oxford Nanopore Technologies and is based on the fuzzy Bruijn graph (FBG) algorithm. Secondly, to eliminate the high error rate of the PacBio long reads, we further polished the PacBio-only assembled sequences using Illumina reads as following. All the Illumina reads were mapped to the PacBio-only assembly with BWA-mem [57], which was further corrected with 2-round Pilon (v1.21) correction [58, 59]. Thirdly, because the polished assembly still contained a number of shorter contigs with significantly lower coverage, which perhaps represents the high heterozygous regions that were not merged to equivalent segments in the

homologous chromosomes, we used a looser cutoff for identity (> 90%) to merge the contigs with lower coverage and smaller size (size < 1000 bp and coverage < 50 or size < 10000 bp and coverage < 35) into the longer contigs as the previously reported [14]. Fourthly, the raw reads generated from the Hi-C sequencing were mapped to the polished assembled genome using Juicer [60] and 3D *de novo* assembly [37] softwares to improve the assembly. Approximately 90.50% of contigs were anchored onto 30 super-scaffolds (**Fig. 1b** & **Table S3**), which quite possibly correspond to the 30 chromosomes as reported by cytogenetic karyotype [53]. Finally, we obtained the chromosomal-level high-quality assembly of *P. bianor* with total length of ~402.00 Mb and the longest scaffold N50 (12.51 M) among the published butterfly genomes (**Table 1** & **Table S4**). The assembled genome accounts for 85% of estimated genome size (473.07 Mb) by the k-mer distribution analysis (**Table S2**).

Quality evaluation of assembled genome

The assembled quality was evaluated using three methods as following. Firstly, The completeness of the assembly was evaluated by Benchmarking Universal Single-Copy Orthologs (BUSCO) (version 2.0; BUSCO, RRID:SCR 015008) [61] software. The BUSCO data showed that *P. bianor* assembly covered 96.90% of the core genes with 96.30% covered genes complete (**Table S5**), which are similar to those published high quality butterfly genomes (**Table 1**). We also checked the mapping rates of Illumina and PacBio reads to the *P. bianor* assembly by BWA [57] and BLASR [62], and found high mapping rate of 96.31% and 96.86%, respectively (**Table S6** & **Table S7**). Thirdly, we compared syntenic relationships between gemomes of *P. bianor* and *P. xuthus* (**Fig. 1c**) and found that 94.96% of the *P. bianor* assembled genome sequences can be aligned (1:1) to the *P. xuthus* reference genome. All these results suggest that

the assembled *P. bianor* genome is of high quality (including completeness, base level continuity and accuracy) (**Table 1**).

Genome annotation

Repetitive sequences including tandem repeats and transposable elements (TEs) were searched in the *P. bianor* assembled genome. Firstly, we used Tandem Repeat Finder (version 4.07b; with 2 7 7 80 10 50 2000 -d -h parameters) [63] to annotate the tandem repeats. Then, TEs were identified using a combination of *de novo* and homology-based approaches at both the DNA and protein levels. At the DNA level, we used RepeatModeler (version 1.0.4; RepeatModeler, RRID:SCR_015027) [64] to construct a *de novo* repeat library, which built a repeat consensus database with classification information, and then we adopted RepeatMasker (version 4.0.5) [65] to search similar TEs against the known Repbase TE library (version 16.02) [66] and *de novo* repeat library. We also used LTR_FINDER (LTR Finder, RRID:SCR_015247) [67] to find long terminal repeats. At the protein level, software RepeatProteinMask [65] was used to search the assembled *P. bianor* genome against the TE protein database using a WU-BLASTX engine. Finally, we identified and masked 55.30% of the *P. bianor* assembly as repeat regions (**Table S8**), which is the highest in published butterfly genomes (**Table 1**). Among all TEs, the most abundant class of repetitive elements is long interspersed nuclear elements (LINEs, 14.22%), and the next is DNA transposons (8.81%) (**Table S9**). Compared with the reference genomes of other swallowtail butterflies, LINEs, DNA transposons and long terminal repeats (LTRs) of repeats have expanded in *P. bianor* genome (**Fig. 2a**).

To annotate protein-coding genes of *P. bianor*, we used both *de novo* and homology-based gene prediction approaches. For *de novo* gene prediction, the repeat-masked genome was analyzed by SNAP (version 2006-07-28) [68], GENSCAN (version 1.0) [69], glimmerHMM (version 3.0.3) [70], and AUGUSTUS (version 2.5.5; Augustus: Gene Prediction, RRID:SCR 008417) [71]. For homology-based predictions, the protein sequences from eight insects including beetle *Tribolium castaneum* [72], fruit fly *Drosophila melanogaster* [73], silkworm *Bombyx mori* [74], moth *Helicoverpa armigera* [75], and four butterflies *Papilio polytes* [23], *Papilio xuthus* [9], *Heliconius melpomene* [24] and *Danaus plexippus* [20], were used as templates for homology-based gene prediction. Then we used TBLASTN [76] with an E-value cut-off of 1e-5 to align the protein sequences of the reference gene set to *P. bianor* genome, and GeneWise (v2.2.0) [77] to perform more precise alignment. Gene sequences with length < 150 bp or percent identity < 25% were removed. EvidenceModeler software (EVM, version 1.1.1) [78] was used to integrate the genes predicted by the homology and *de novo* approaches and generate a comprehensive, non-redundant gene set. Finally, 15,375 protein-coding genes were annotated in the assembled *P. bianor* genome (**Table S10**), which is similar to those published reference genomes of other swallowtail butterflies (**Fig. S2**).

The KEGG, TrEMBL, SwissProt and Cog databases were searched for best matches to *P. bianor* the protein sequences yielded by EVM software, using BLASTP (version 2.2.26) with an (E)-value cutoff of 1e-5, and Pfam, PRINTS, ProDom and SMART databases were searched for known motifs and domains in our sequences using InterProScan software (version 5.18-57.0; InterProScan, RRID:SCR_005829) [79]. We also searched all predicted gene sequences to GenBank nonredundant protein (nr)

using BLASTN (RRID:SCR 001598) with a maximal e-value of 1e-5. In sum, 13,343 genes were annotated with at least 1 related function, which accounts for about 86.78% of the *P. bianor* annotated genes (**Table S11**).

Gene family identification and phylogenetic analysis

We use OrthoMCL (version 2.0.9; OrthoMCL DB: Ortholog Groups of Protein Sequences, RRID:SCR 007839) [80] to cluster the *P. bianor* annotated genes with an (E)-value cutoff of 1 e-5, and Markov Chain Clustering with default inflation parameter in an all-to-all BLASTP analysis of entries for the reference genomes of six swallowtail butterflies including *P. bianor* in this study and other five published so far (*P. polytes*, *P. xuthus*, *P. machaon*, *P. glaucus*, and *P. memnon*). The result showed that 293 gene families were specific to *P. bianor* (**Fig. 2b**). Using Computational Analysis of gene Family Evolution (CAFE; version 4.0.1) [81], we also identified 375 expanded gene families and 1863 contracted gene families in *P. bianor*. The *P. bianor* expanded gene families were enriched in 17 GO categories and the contracted gene families were enriched in 14 GO categories, most of which are related to oxygen metabolism (**Table S12** & **Table S13**).

To reveal phylogenetic position of *P. bianor* among Papilionoidea, we selected 16 butterfly species in five families (Papilionidae (6): *Papilio xuthus*, *Papilio polytes, Papilio machaon*, *Papilio glaucus*, *Papilio memnon*; Hesperiidae (1): *Lerema accius*; Pieridae (2): *Phoebis sennae*, *Pieris rapae*; Nymphalidae (2): *Bicyclus anynana, Heliconius melpomene*; Riodinidae (2): *Calephelis nemesis*, *Calephelis virginiensis*; Lycaenidae (1): *Calycopis cecrops*) [9, 13-15, 17, 21, 23, 24, 26-28] with 2 moths (*Bombyx mori* [74], *Helicoverpa armigera* [75]) as outgroups for phylogenetic

analysis. 1378 one to one single orthologs were identified from these 14 species and their nucleic acid sequences were aligned using PRANK (version 3.8.31) [82] to construct the phylogenetic trees using RAxML (version 7.2.8; RAxML, RRID:SCR 006086) [83] by choosing the GTR+G+I model. The phylogeny was further analyzed by PAML MCMCtree (version 4.5; PAML, RRID:SCR 014932) program [84], and calibrated with published timings for the divergence of difference species [85]. Our phylogenetic tree showed that *P. bianor* cluster at the base of *P. machaon* and *P. xuhtus*, and diverged from them 23 million years ago (mya); all *Papilio* species is a monophyly, and diverged from other butterflies approximately 41.07-56.86 mya (**Fig. 2c**). This tree is largely consistent with those constructed from cytochrome oxidases I (COI), cytochrome oxidases II (COII) and elongation factor 1α (EF-1α) [86, 87], and from 425 loci from two outgroups and 173 species of butterflies[88].

We also inferred demographic histories of *P. bianor* by SNP calling of Illumina short reads against assembled genome using the Pairwise Sequentially Markovian Coalescence (PSMC) analysis [89] (0.1×10^{-8} mutations per site per generation calculated by r8s [90]; three or four generations per year [47]). Our result suggested that the effective population size increased significantly from the last interglacial period (LIG, approximately 0.1 million years before present) to its maximum at the last glacial maximum (LGM, approximately 0.01 million years before present) (**Fig. 2d**). We infer that the population expansion of this species possibly results from the decrease of its natural enemies (e.g. birds or lizard) and from its adaptation to diverse climate environments during LIG and LGM.

Conclusion

We present the chromosomal-level genome assembly of *P. bianor* with the contig and scaffold N50 of 5.50 Mb and 12.51 Mb, respectively. The assembled genome included 15,375 protein-coding genes, 293 species-specific gene families, 375 expanded gene families and 1863 contracted gene families. The *P. bianor* diverged from other *Papilio* approximately 23.69-36.04 mya. Our results also show that the effective population size of *P. bianor* increased significantly during the glacial period. Our results lay the foundation for exploring the special biological features of the Chinese peacock butterfly, and also provide a useful data source for comparative genomics and phylogenomics among butterflies and Lepidopterans.

Availability of supporting data

The raw reads have been deposited at NCBI in the sequence read archive (SRA) under BioProject Number: PRJNA530186. The chromosome-level genome, annotation, and other supporting data are also available via the *GigaScience* database, *Giga*DB.

Abbreviations

bp: base pair; kb: kilo base; Mb: mega base; Gb: giga base; PE: paired-end; BUSCO: Benchmarking Universal Single-Copy Orthologs; TE: transposable element; GO: gene ontology; KEGG: Kyoto Encyclopedia of Genes and Genomes.

Competing interests

The authors declare that there have no competing interests.

Author contributions

X.L., W.W conceived and supervised the study. J.H., Z.D., Z.C., G.L. fed and collected the samples. G.L., J.H. extracted the genomic DNA. S.L., X.D. assembled the genome. S.L., J.Y., F.X. carried out the quality assessment, repeat annotation, and gene annotation. J.Y., F.X., J.M. carried out evolutionary analyses. S.L. uploaded the raw read data, genome assembly, and annotation in the GenBank and GigaScience (GigaDB) databases. S.L., X.L., W.W. wrote the manuscript. All authors read and approved the final manuscript.

Acknowledgements

This work was supported by grants from the National Natural Science Foundation of China (No. 31621062) (to WW), the Chinese Academy of Sciences (XDB13000000 (to WW), and CAS "Light of West China" (to LXY).

References

1. Boggs CL, Watt WB and Ehrlich PR. Butterflies: ecology and evolution taking flight. University of Chicago Press; 2003.
2. Joron M and Mallet JLB. Diversity in mimicry: paradox or paradigm? Trends in ecology & evolution. 1998;13 11:461-6. doi:Doi 10.1016/S0169-5347(98)01483-9.
3. Nijhout HF. The development and evolution of butterfly wing patterns. Smithson Inst. 1991;293.
4. Heikkila M, Kaila L, Mutanen M, Pena C and Wahlberg N. Cretaceous origin and repeated tertiary diversification of the redefined butterflies. Proceedings Biological sciences. 2012;279 1731:1093-9. doi:10.1098/rspb.2011.1430.
5. Kawahara AY and Breinholt JW. Phylogenomics provides strong evidence for relationships of butterflies and moths. Proceedings Biological sciences. 2014;281 1788:20140970. doi:10.1098/rspb.2014.0970.
6. Mitter C, Davis DR and Cummings MP. Phylogeny and Evolution of Lepidoptera. Annual review of entomology. 2017;62:265-83. doi:10.1146/annurev-ento-031616-035125.
7. Darwin C. The Origin of Species; And, the Descent of Man. Modern library; 1859.
8. Bates H. New species of butterflies from Guatemala and Panama, collected by Osbert Salvin and F. du Cane Godman, Esqs. Entomologist's monthly Magazine. 1864;1 1/7:1-164.
9. Li X, Fan D, Zhang W, Liu G, Zhang L, Zhao L, et al. Outbred genome sequencing and CRISPR/Cas9 gene editing in butterflies. Nature communications. 2015;6:8212. doi:10.1038/ncomms9212.
10. Zhang LL and Reed RD. Genome editing in butterflies reveals that spalt promotes and Distal-less represses eyespot colour patterns. Nature communications. 2016;7 doi:10.1038/Ncomms11769.
11. Markert MJ, Zhang Y, Enuameh MS, Reppert SM, Wolfe SA and Merlin C. Genomic Access to Monarch Migration Using TALEN and CRISPR/Cas9-Mediated Targeted Mutagenesis. G3-Genes Genom Genet. 2016;6 4:905-15. doi:10.1534/g3.116.027029.
12. van Nieukerken EJ, Kaila L, Kitching IJ, Kristensen NP, Lees D, Minet J, et al. Order Lepidoptera Linnaeus, 1758. Zootaxa. 2011;3148:212-21.
13. Cong Q, Borek D, Otwinowski Z and Grishin NV. Skipper genome sheds light on unique phenotypic traits and phylogeny. BMC genomics. 2015;16:639. doi:10.1186/s12864-015-1846-0.
14. Cong Q, Borek D, Otwinowski Z and Grishin NV. Tiger Swallowtail Genome Reveals Mechanisms for Speciation and Caterpillar Chemical Defense. Cell reports. 2015;10 6:910-9. doi:10.1016/j.celrep.2015.01.026.
15. Shen J, Cong Q, Kinch LN, Borek D, Otwinowski Z and Grishin NV. Complete genome of *Pieris rapae*, a resilient alien, a cabbage pest, and a source of anti-cancer proteins. F1000Res. 2016;5:2631. doi:10.12688/f1000research.9765.1.
16. Cong Q, Li W, Borek D, Otwinowski Z and Grishin NV. The Bear Giant-Skipper genome suggests genetic adaptations to living inside yucca roots. Molecular genetics and genomics : MGG. 2018; doi:10.1007/s00438-018-1494-6.

17. Iijima T, Kajitani R, Komata S, Lin CP, Sota T, Itoh T, et al. Parallel evolution of Batesian mimicry supergene in two *Papilio* butterflies, *P. polytes* and *P. memnon*. Science advances. 2018;4 4 doi:10.1126/sciadv.aao5416.
18. Zhan S, Merlin C, Boore JL and Reppert SM. The monarch butterfly genome yields insights into long-distance migration. Cell. 2011;147 5:1171-85. doi:10.1016/j.cell.2011.09.052.
19. Hill JA, Neethiraj R, Rastas P, Clark N, Morehouse N, de la Paz Celorio-Mancera M, et al. A butterfly chromonome reveals selection dynamics during extensive and cryptic chromosomal reshuffling. bioRxiv. 2018:233700.
20. Zhan S, Zhang W, Niitepold K, Hsu J, Haeger JF, Zalucki MP, et al. The genetics of monarch butterfly migration and warning colouration. Nature. 2014;514 7522:317-21. doi:10.1038/nature13812.
21. Cong Q, Shen JH, Warren AD, Borek D, Otwinowski Z and Grishin NV. Speciation in Cloudless Sulphurs Gleaned from Complete Genomes. Genome biology and evolution. 2016;8 3:915-31. doi:10.1093/gbe/evw045.
22. Talla V, Suh A, Kalsoom F, Dinca V, Vila R, Friberg M, et al. Rapid Increase in Genome Size as a Consequence of Transposable Element Hyperactivity in Wood-White (Leptidea) Butterflies. Genome biology and evolution. 2017;9 10:2491-505. doi:10.1093/gbe/evx163.
23. Nishikawa H, Iijima T, Kajitani R, Yamaguchi J, Ando T, Suzuki Y, et al. A genetic mechanism for female-limited Batesian mimicry in *Papilio* butterfly. Nature genetics. 2015;47 4:405-U169. doi:10.1038/ng.3241.
24. Dasmahapatra KK, Walters JR, Briscoe AD, Davey JW, Whibley A, Nadeau NJ, et al. Butterfly genome reveals promiscuous exchange of mimicry adaptations among species. Nature. 2012;487 7405:94-8. doi:10.1038/nature11041.
25. Ahola V, Lehtonen R, Somervuo P, Salmela L, Koskinen P, Rastas P, et al. The Glanville fritillary genome retains an ancient karyotype and reveals selective chromosomal fusions in Lepidoptera. Nature communications. 2014;5 doi:10.1038/Ncomms5737.
26. Cong Q, Shen JH, Borek D, Robbins RK, Otwinowski Z and Grishin NV. Complete genomes of Hairstreak butterflies, their speciation, and nucleo-mitochondrial incongruence. Scientific reports. 2016;6 doi:10.1038/Srep24863.
27. Cong Q, Shen JH, Li WL, Borek D, Otwinowski Z and Grishin NV. The first complete genomes of Metalmarks and the classification of butterfly families. Genomics. 2017;109 5-6:485-93. doi:10.1016/j.ygeno.2017.07.006.
28. Nowell RW, Elsworth B, Oostra V, Zwaan BJ, Wheat CW, Saastamoinen M, et al. A high-coverage draft genome of the mycalesine butterfly *Bicyclus anynana*. GigaScience. 2017;6 7 doi:10.1093/gigascience/gix035.
29. Mallet J. New genomes clarify mimicry evolution. Nature genetics. 2015;47 4:306-7. doi:10.1038/ng.3260.
30. Davey JW, Chouteau M, Barker SL, Maroja L, Baxter SW, Simpson F, et al. Major Improvements to the *Heliconius melpomene* Genome Assembly Used to Confirm 10 Chromosome Fusion Events in 6 Million Years of Butterfly Evolution. G3-Genes Genom Genet. 2016;6 3:695-708. doi:10.1534/g3.115.023655.
31. Loehlin DW and Carroll SB. EVOLUTIONARY BIOLOGY Sex, lies and butterflies. Nature. 2014;507 7491:172-3. doi:Doi 10.1038/Nature13066.

32. Brunetti CR, Selegue JE, Monteiro A, French V, Brakefield PM and Carroll SB. The generation and diversification of butterfly eyespot color patterns. Current Biology. 2001;11 20:1578-85. doi:Doi 10.1016/S0960-9822(01)00502-4.

33. VanBuren R, Bryant D, Edger PP, Tang HB, Burgess D, Challabathula D, et al. Single-molecule sequencing of the desiccation-tolerant grass Oropetium thomaeum. Nature. 2015;527 7579:508-U209. doi:10.1038/nature15714.

34. Andere AA, Ii RNP, Ray DA and Picard CJ. Genome sequence of Phormia regina Meigen (Diptera: Calliphoridae): implications for medical, veterinary and forensic research. BMC genomics. 2016;17 doi:10.1186/s12864-016-3187-z.

35. Belaghzal H, Dekker J and Gibcus JH. Hi-C 2.0: An optimized Hi-C procedure for high-resolution genome-wide mapping of chromosome conformation. Methods. 2017;123:56-65. doi:10.1016/j.ymeth.2017.04.004.

36. Chakraborty M, VanKuren NW, Zhao R, Zhang XW, Kalsow S and Emerson JJ. Hidden genetic variation shapes the structure of functional elements in *Drosophila*. Nature genetics. 2018;50 1:20-+. doi:10.1038/s41588-017-0010-y.

37. Dudchenko O, Batra SS, Omer AD, Nyquist SK, Hoeger M, Durand NC, et al. De novo assembly of the Aedes aegypti genome using Hi-C yields chromosome-length scaffolds. Science. 2017;356 6333:92-5. doi:10.1126/science.aal3327.

38. Chen WB, Yang XW, Tetreau G, Song XZ, Coutu C, Hegedus D, et al. A high-quality chromosome-level genome assembly of a generalist herbivore, Trichoplusia ni. Molecular ecology resources. 2019;19 2:485-96. doi:10.1111/1755-0998.12966.

39. Xiang H, Liu XJ, Li MW, Zhu YN, Wang LZ, Cui Y, et al. The evolutionary road from wild moth to domestic silkworm. Nature ecology & evolution. 2018;2 8:1268-79. doi:10.1038/s41559-018-0593-4.

40. Wu C. Fauna Sinica Insect Vol. 25 Lepidoptera Papilionidae. Beijing: Science Press, 2001.

41. Sinev SY. Catalogue of the Lepidoptera of Russia. Ed. SY Sinev. KMK, Saint-Petersburg-Moscow, 2008.

42. Chou I. Monograph of Chinese butterflies. Zhengzhou: Henan Scientific and Technological Publishing House. 1994:1-854.

43. Ono H, Nishida R and Kuwahara Y. Oviposition stimulant for a Rutaceae-feeding swallowtail butterfly, *Papilio bianor* (Lepidoptera: Papilionidae): Hydroxycinnamic acid derivative from *Orixa japonica*. Applied Entomology and Zoology. 2000;35 1:119-23.

44. Perveen F, Khan A and Sikander. Characteristics of butterfly (Lepidoptera) fauna from Kabal, Swat, Pakistan. Journal of Entomology and Zoology Studies. 2014;2 1:56-69.

45. Yokoyama I, Endo K, Yamanaka A and Kumagai K. Species-specificity in the action of big and small prothoracicotropic hormones (PTTHs) of the swallowtail butterflies, *Papilio xuthus*, *P. machaon*, *P. bianor* and *P. helenus*. Zoological Science. 1996;13 3:449-54. doi:Doi 10.2108/Zsj.13.449.

46. Ono H, Nishida R and Kuwahara Y. A dihydroxy-gamma-lactone as an oviposition stimulant for the swallotail butterfly, *Papilio bianor*, from the Rutaceous plant, Orixa japonica. Biosci Biotech Bioch. 2000;64 9:1970-3. doi:Doi 10.1271/Bbb.64.1970.

47. Dongsheng L. A Preliminary Observation on the Artificial Rearing of Xinyang *Papilio bianor*. JOURNAL OF XINYANG TEACHERS COLLEGE (NATURAL SCIENCE EDITION). 1997;2.
48. Lixin Z, Xiaobing W, Chunsheng W and Banghe Y. Phylogenetic evaluation of *Papilio bianor* and *P. polyctor* (Lepidoptera: Papilionidae). Oriental Insects. 2009;43 1:25-32.
49. Hou LX, Ying S, Yang XW, Yu Z, Li HM and Qin XM. The complete mitochondrial genome of *Papilio bianor* (Lepidoptera: Papilionidae), and its phylogenetic position within Papilionidae. Mitochondrial DNA Part A. 2016;27 1:102-3. doi:10.3109/19401736.2013.873923.
50. Ae S. Some problems in hybrids between *Papilio bianor* and *P. maackii*. Academia (Nanzan Univ). 1962;33:21-8.
51. CHANG Y-J. A study on hybridization of two subspecies of *Papilio bianor* (Lepidoptera, Papilionidae) in Taiwan. Lepidoptera Science. 1990;41 1:1-6.
52. Yamada A. A study of interspecific hybrids between *Papilio bianor* and *P. maackii*. The nature and insects. 1977;12:27-8.
53. Maeki K and Makino S. Chromosome numbers of some Japanese Rhopalocera. Lepid news. 1953;7:36-8.
54. Dong Y, Zhu L-X, Wu Y-f and Wu X-B. The complete mitochondrial genome of the Chinese peacock, *Papilio bianor* (Insecta: Lepidoptera: Papilionidae). Mitochondrial DNA. 2013;24 6:636-8.
55. Li R, Fan W, Tian G, Zhu H, He L, Cai J, et al. The sequence and de novo assembly of the giant panda genome. Nature. 2010;463 7279:311-7. doi:10.1038/nature08696.
56. Ruan J and Li H. Fast and accurate long-read assembly with wtdbg2. BioRxiv. 2019:530972.
57. Li H. Aligning sequence reads, clone sequences and assembly contigs with BWA-MEM. arXiv preprint arXiv:13033997. 2013.
58. Walker BJ, Abeel T, Shea T, Priest M, Abouelliel A, Sakthikumar S, et al. Pilon: An Integrated Tool for Comprehensive Microbial Variant Detection and Genome Assembly Improvement. PloS one. 2014;9 11 doi:10.1371/journal.pone.0112963.
59. Vaser R, Sovic I, Nagarajan N and Sikic M. Fast and accurate de novo genome assembly from long uncorrected reads. Genome research. 2017;27 5:737-46. doi:10.1101/gr.214270.116.
60. Durand NC, Shamim MS, Machol I, Rao SSP, Huntley MH, Lander ES, et al. Juicer Provides a One-Click System for Analyzing Loop-Resolution Hi-C Experiments. Cell Syst. 2016;3 1:95-8. doi:10.1016/j.cels.2016.07.002.
61. Simao FA, Waterhouse RM, Ioannidis P, Kriventseva EV and Zdobnov EM. BUSCO: assessing genome assembly and annotation completeness with single-copy orthologs. Bioinformatics. 2015;31 19:3210-2. doi:10.1093/bioinformatics/btv351.
62. Chaisson MJ and Tesler G. Mapping single molecule sequencing reads using basic local alignment with successive refinement (BLASR): application and theory. BMC bioinformatics. 2012;13 doi:10.1186/1471-2105-13-238.
63. Benson G. Tandem repeats finder: a program to analyze DNA sequences. Nucleic acids research. 1999;27 2:573-80. doi:Doi 10.1093/Nar/27.2.573.
64. Smith A, Hubley R and Green P. RepeatMasker Open-4.0.(2013-2015). 2016.
65. Chen N. Using RepeatMasker to identify repetitive elements in genomic sequences. Current protocols in bioinformatics. 2004;5 1:4.10. 1-4.. 4.

66. Bao WD, Kojima KK and Kohany O. Repbase Update, a database of repetitive elements in eukaryotic genomes. Mobile DNA-Uk. 2015;6 doi:10.1186/s13100-015-0041-9.
67. Xu Z and Wang H. LTR_FINDER: an efficient tool for the prediction of full-length LTR retrotransposons. Nucleic acids research. 2007;35:W265-W8. doi:10.1093/nar/gkm286.
68. Korf I. Gene finding in novel genomes. BMC bioinformatics. 2004;5 doi:Doi 10.1186/1471-2105-5-59.
69. Burge C and Karlin S. Prediction of complete gene structures in human genomic DNA. J Mol Biol. 1997;268 1:78-94. doi:DOI 10.1006/jmbi.1997.0951.
70. Majoros WH, Pertea M and Salzberg SL. TigrScan and GlimmerHMM: two open source ab initio eukaryotic gene-finders. Bioinformatics. 2004;20 16:2878-9. doi:10.1093/bioinformatics/bth315.
71. Stanke M, Keller O, Gunduz I, Hayes A, Waack S and Morgenstern B. AUGUSTUS: ab initio prediction of alternative transcripts. Nucleic acids research. 2006;34:W435-W9. doi:10.1093/nar/gkl200.
72. Tribolium Genome Sequencing C, Richards S, Gibbs RA, Weinstock GM, Brown SJ, Denell R, et al. The genome of the model beetle and pest *Tribolium castaneum*. Nature. 2008;452 7190:949-55. doi:10.1038/nature06784.
73. Adams MD, Celniker SE, Holt RA, Evans CA, Gocayne JD, Amanatides PG, et al. The genome sequence of *Drosophila melanogaster*. Science. 2000;287 5461:2185-95.
74. Duan J, Li R, Cheng D, Fan W, Zha X, Cheng T, et al. SilkDB v2.0: a platform for silkworm (*Bombyx mori*) genome biology. Nucleic acids research. 2010;38 Database issue:D453-6. doi:10.1093/nar/gkp801.
75. Pearce SL, Clarke DF, East PD, Elfekih S, Gordon KHJ, Jermiin LS, et al. Genomic innovations, transcriptional plasticity and gene loss underlying the evolution and divergence of two highly polyphagous and invasive *Helicoverpa* pest species. BMC biology. 2017;15 1:63. doi:10.1186/s12915-017-0402-6.
76. Altschul SF, Madden TL, Schaffer AA, Zhang JH, Zhang Z, Miller W, et al. Gapped BLAST and PSI-BLAST: a new generation of protein database search programs. Nucleic acids research. 1997;25 17:3389-402. doi:DOI 10.1093/nar/25.17.3389.
77. Birney E, Clamp M and Durbin R. GeneWise and genomewise. Genome research. 2004;14 5:988-95. doi:10.1101/gr.1865504.
78. Haas BJ, Salzberg SL, Zhu W, Pertea M, Allen JE, Orvis J, et al. Automated eukaryotic gene structure annotation using EVidenceModeler and the program to assemble spliced alignments. Genome biology. 2008;9 1 doi:10.1186/Gb-2008-9-1-R7.
79. Jones P, Binns D, Chang H-Y, Fraser M, Li W, McAnulla C, et al. InterProScan 5: genome-scale protein function classification. Bioinformatics. 2014;30 9:1236-40.
80. Li L, Stoeckert CJ and Roos DS. OrthoMCL: identification of ortholog groups for eukaryotic genomes. Genome research. 2003;13 9:2178-89.
81. De Bie T, Cristianini N, Demuth JP and Hahn MW. CAFE: a computational tool for the study of gene family evolution. Bioinformatics. 2006;22 10:1269-71.
82. Loytynoja A and Goldman N. An algorithm for progressive multiple alignment of sequences with insertions. Proceedings of the National Academy

of Sciences of the United States of America. 2005;102 30:10557-62. doi:10.1073/pnas.0409137102.

83. Stamatakis A. RAxML version 8: a tool for phylogenetic analysis and post-analysis of large phylogenies. Bioinformatics. 2014;30 9:1312-3. doi:10.1093/bioinformatics/btu033.

84. Yang ZH. PAML 4: Phylogenetic analysis by maximum likelihood. Molecular biology and evolution. 2007;24 8:1586-91. doi:10.1093/molbev/msm088.

85. Kumar S, Stecher G, Suleski M and Hedges SB. TimeTree: A Resource for Timelines, Timetrees, and Divergence Times. Molecular biology and evolution. 2017;34 7:1812-9. doi:10.1093/molbev/msx116.

86. Zakharov EV, Caterino MS and Sperling FA. Molecular phylogeny, historical biogeography, and divergence time estimates for swallowtail butterflies of the genus *Papilio* (Lepidoptera: Papilionidae). Systematic biology. 2004;53 2:278-98.

87. Dupuis JR and Sperling FA. Repeated reticulate evolution in North American *Papilio machaon* group swallowtail butterflies. PloS one. 2015;10 10:e0141882.

88. Espeland M, Breinholt J, Willmott KR, Warren AD, Vila R, Toussaint EFA, et al. A Comprehensive and Dated Phylogenomic Analysis of Butterflies. Current Biology. 2018;28 5:770-+. doi:10.1016/j.cub.2018.01.061.

89. Li H and Durbin R. Inference of human population history from individual whole-genome sequences. Nature. 2011;475 7357:493-U84. doi:10.1038/nature10231.

90. Sanderson MJ. r8s: inferring absolute rates of molecular evolution and divergence times in the absence of a molecular clock. Bioinformatics. 2003;19 2:301-2. doi:DOI 10.1093/bioinformatics/19.2.301.

Table 1: Comparison of quality and composition of different butterfly genomes.

Family	Species	Genome size (Mb)	Genome size without gap (Mb)	Heterozygosity [a] (%)	Scaffold N50 (kb)	BUSCO [b] (%)	*De novo* assembled transcripts [a] (%)	GC content (%)	Repeat (%)	Exon (%)	Intron (%)	Number of proteins (k)
Papilionidae	*Papilio bianor*	**402**	**402**	**1.8**	**12813**	**96.3**	NA	**36.6**	**55.3**	**5.05**	**27.44**	**15.4**
	Papilio xuthus	244	238	NA	6199	97.6	NA	33.8	22.4	8.59	45.50	13.1
	Papilio machaon	281	266	1.2	1150	95.5	98	32.3	22.3	7.37	30.36	15.5
	Papilio polytes	227	218	NA	3672	91.8	NA	34.0	23.8	12.97	48.58	12.2
	Papilio memnon	233	219	NA	5457	96.6	NA	32.8	22.5	11.31	43.17	12.4
	Papilio glaucus	375	361	2.3	231	95.5	98	35.4	22.0	5.07	25.60	15.7
Hesperiidae	*Achalarus lyciades*	567	536	1.5	558	97.3	98	35.3	25.0	3.57	28.40	15.9
	Lerema accius	298	290	1.5	525	95.1	98	34.4	15.5	6.96	31.60	17.4
	Megathymus ursus violae	429	427	0.1	4153	98.3	99	34.7	25.8	4.59	30.90	14.1
Pieridae	*Pieris rapae*	246	243	1.5	617	98.0	99	32.7	22.7	7.91	33.30	13.2
	Phoebis sennae	406	347	1.2	257	97.7	97	39.0	17.2	6.20	25.50	16.5
Nymphalidae	*Danaus plexippus*	249	242	0.6	716	98.0	96	31.6	16.3	8.40	28.10	15.1
	Heliconius melpomene	274	270	NA	194	95.6	NA	32.8	24.9	6.38	25.40	12.8
	Melitaea cinxia	390	361	NA	119	83.0	97	32.6	27.5	4.34	31.20	16.7
	Bicyclus anynana	475	470	NA	638	97.6	NA	36.5	25.8	4.73	38.36	22.6
Riodinidae	*Calephelis nemesis*	809	783	0.5	206	95.6	99	34.9	34.8	2.25	19.60	15.4
	Calephelis virginiensis	855	824	1.3	175	93.9	99	35.0	38.8	2.17	20.50	15.6
Lycaenidae	*Calycopis cecrops*	729	689	1.2	233	95.5	96	37.1	34.0	3.11	24.00	16.5

[a] NA: not available in the referenced citation.
[b] BUSCO is calculated in this study.

Figure legends

Figure 1. Characterization of *Papilio bianor*. (a) Female adult of *P. bianor.* Shown from left to right are: (1) dorsal view, (2) ventral view. (scales = 20.0 mm; Photo by Zhiwei Dong) **(b)** Heatmap of chromosomal interactions. Each chromosome is framed with blue block, and each scaffold is framed with green block. **(c)** Circos plot of *P. bianor* chromosome-level reference genome with the previously released *Papilio xuthus* genome (obtained from Chinese group). Shown from the outermost to innermost are: (1) gene density, (2) repeat element density, (3) GC content, and (4) syntenic regions with *P. xuthus* (left).

Figure 2. Genomic analysis of *Papilio bianor*. (a) Breakdown of the whole-genome assemblies into different segments in *Papilio*. **(b)** Venn diagram of the shared gene families of *Papilio.* **(c)** Maximum Likelihood (ML) phylogenetic tree of Papilionoidea inferred using orthologue genes. The numbers in the square brackets on the nodes are the 95% confidence intervals of divergence time. **(d)** Demographic history of *P. bianor*. "g" indicates generation time in years, and "μ" indicates genomic substitution rate. Pb: *Papilio bianor*; Pgl: *Papilio glaucus*; Pma: *Papilio machaon*; Pme: *Papilio memnon*; Ppol: *Papilio polytes*; Pxu: *Papilio xuthus*.

Figure 1

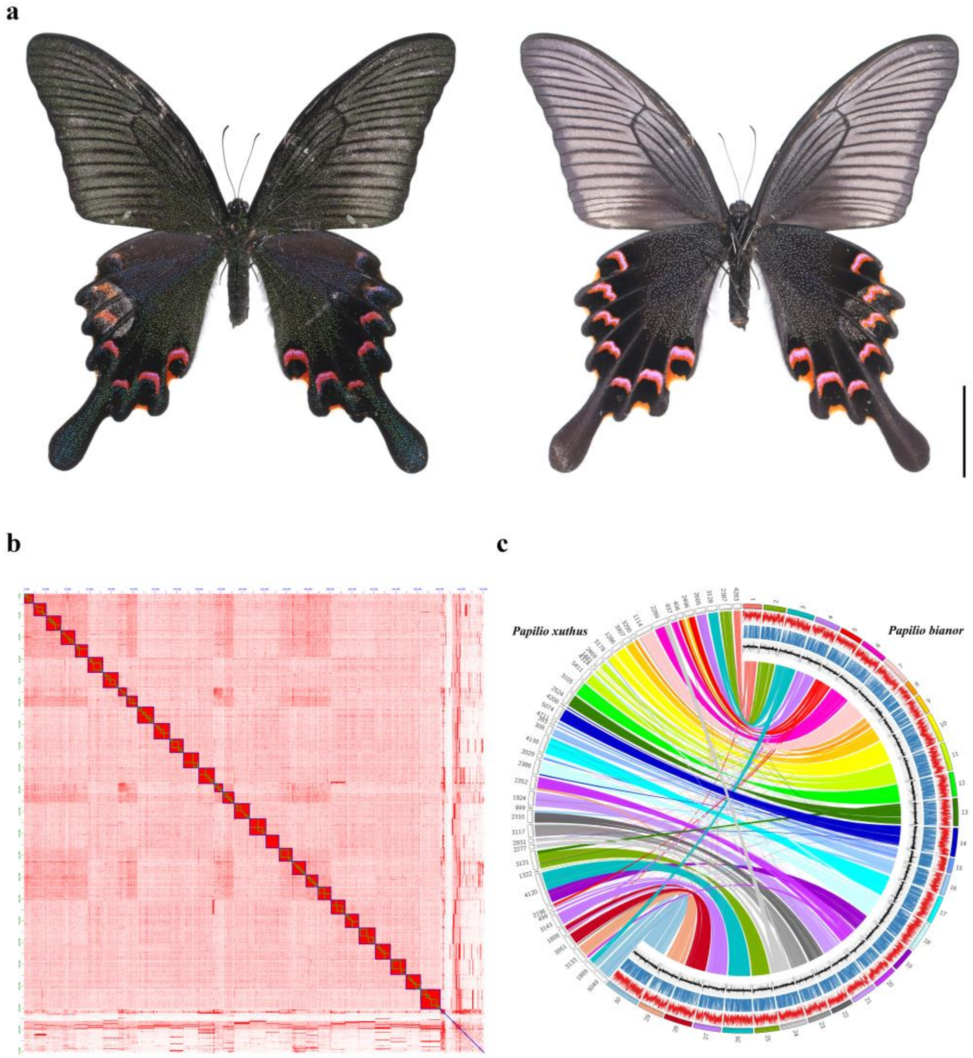

Figure 2

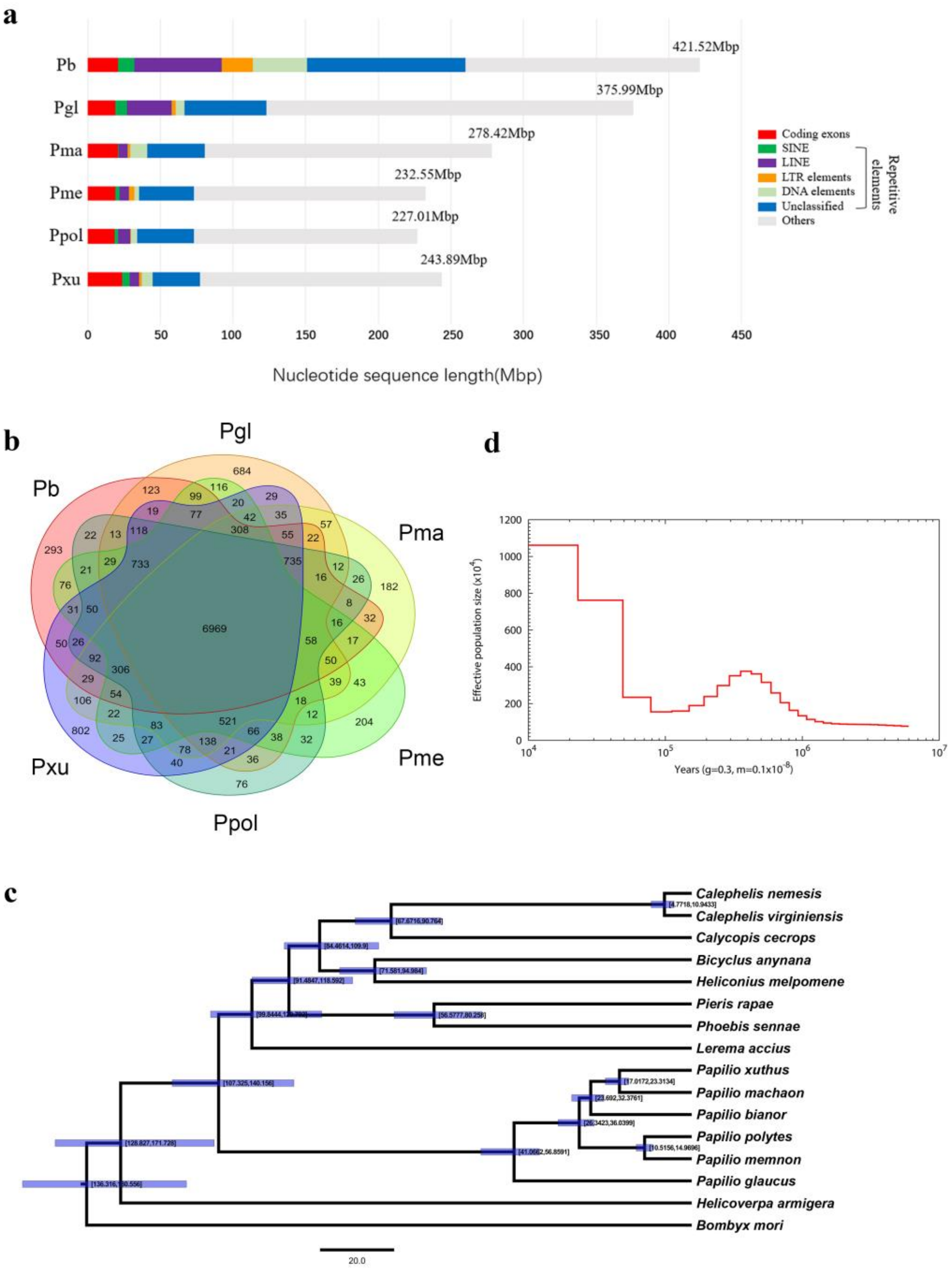
a
Pb
Pgl
Pma
Pme
Ppol
Pxu
421.52Mbp
375.99Mbp
278.42Mbp
232.55Mbp
227.01Mbp
243.89Mbp
0
50
100
150
200
250
300
350
400
450
Nucleotide sequence length(Mbp)
Coding exons
SINE
LINE
LTR elements
DNA elements
Unclassified
Others
Repetitive elements
b
Pgl
Pb
Pma
Pme
Ppol
Pxu
6969
d
Effective population size (x10^4)
Years (g=0.3, m=0.1x10^-8)
c
Calephelis nemesis
Calephelis virginiensis
Calycopis cecrops
Bicyclus anynana
Heliconius melpomene
Pieris rapae
Phoebis sennae
Lerema accius
Papilio xuthus
Papilio machaon
Papilio bianor
Papilio polytes
Papilio memnon
Papilio glaucus
Helicoverpa armigera
Bombyx mori
20.0

Additional files

Figure S1: K-mer (k=17) distribution in *Papilio bianor* genome. The first peak (depth=53) is a heterozygous peak, which is higher than the main peak (depth=26), suggesting the *P. bianor* genome is a highly heterozygous genome. The x-axis is depth (×); the y-axis is the proportion which represents the frequency at that depth divide by the total frequency of all the depth.

Figure S2: The statistics of annotated protein-coding genes of *Papilio.* (a) mRNA length, (b) Coding sequence (CDS) length, (c) exon length, (d) intron length, (e) exon number. The x-axis represents length or number and the y-axis represents the density of genes.

Table S1: The statistics of sequencing data generated for *Papilio bianor* genome. The sequencing depth was calculated by the assembled genome size.

Table S2: Genome size estimation of *Papilio bianor* with K-mer distribution analysis using k=17.

Table S3: The statistics of assembled chromosome-level genome of *Papilio bianor*. The Hi-C data were filtered by HiC-Pro software, and then 6,690,421 pairs of reads could be used in the following analysis, it accounts for 68.04% of the total Hi-C data.

Table S4: The continuity assessment of genome assembly of *Papilio bianor*.

Table S5: The quality evaluation of assembled genome of *Papilio bianor* by BUSCO software.

Table S6: The statistics of mapping ratio of Illumina reads to *Papilio bianor* assembled genome.

Table S7: The statistics of mapping ratio of PacBio reads to *Papilio bianor* assembled genome.

Table S8: The statistics of the annotated repeat sequences in *Papilio bianor* genome.

Table S9: The statistics of the TE contents in *Papilio bianor* genome.

Table S10: The statistics of predicted protein-coding genes in *Papilio bianor* genome.

Table S11: The statistics of gene function annotation in *Papilio bianor* genome.

Table S12: The GO term enrichment of expanded gene families in *Papilio bianor* genome.

Table S13: The GO term enrichment of contracted gene families in *Papilio bianor* genome.

Figure 1 Click here to download Figure Figure 1.jpg

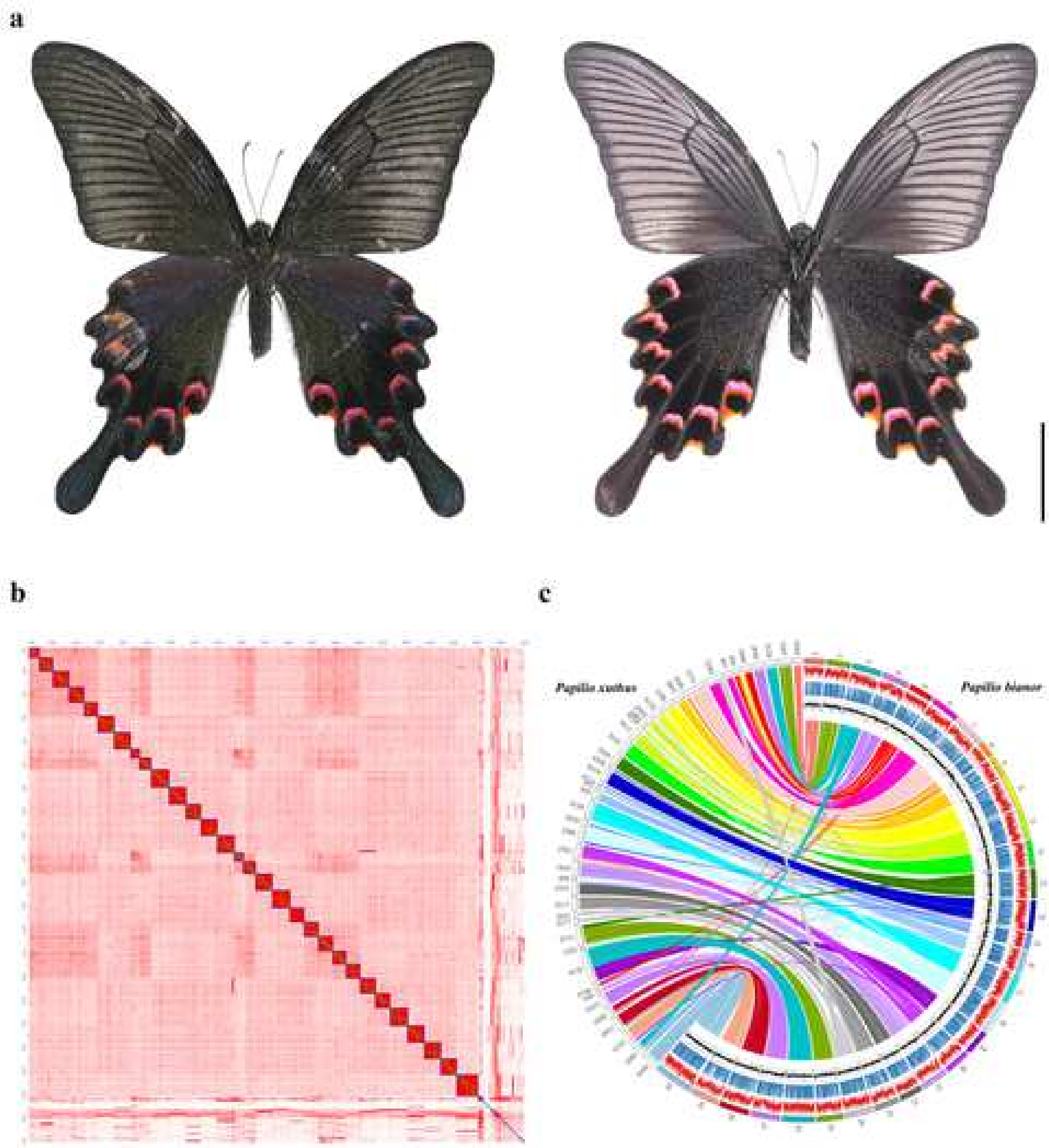

Figure 2

Click here to download Figure Figure 2.jpg

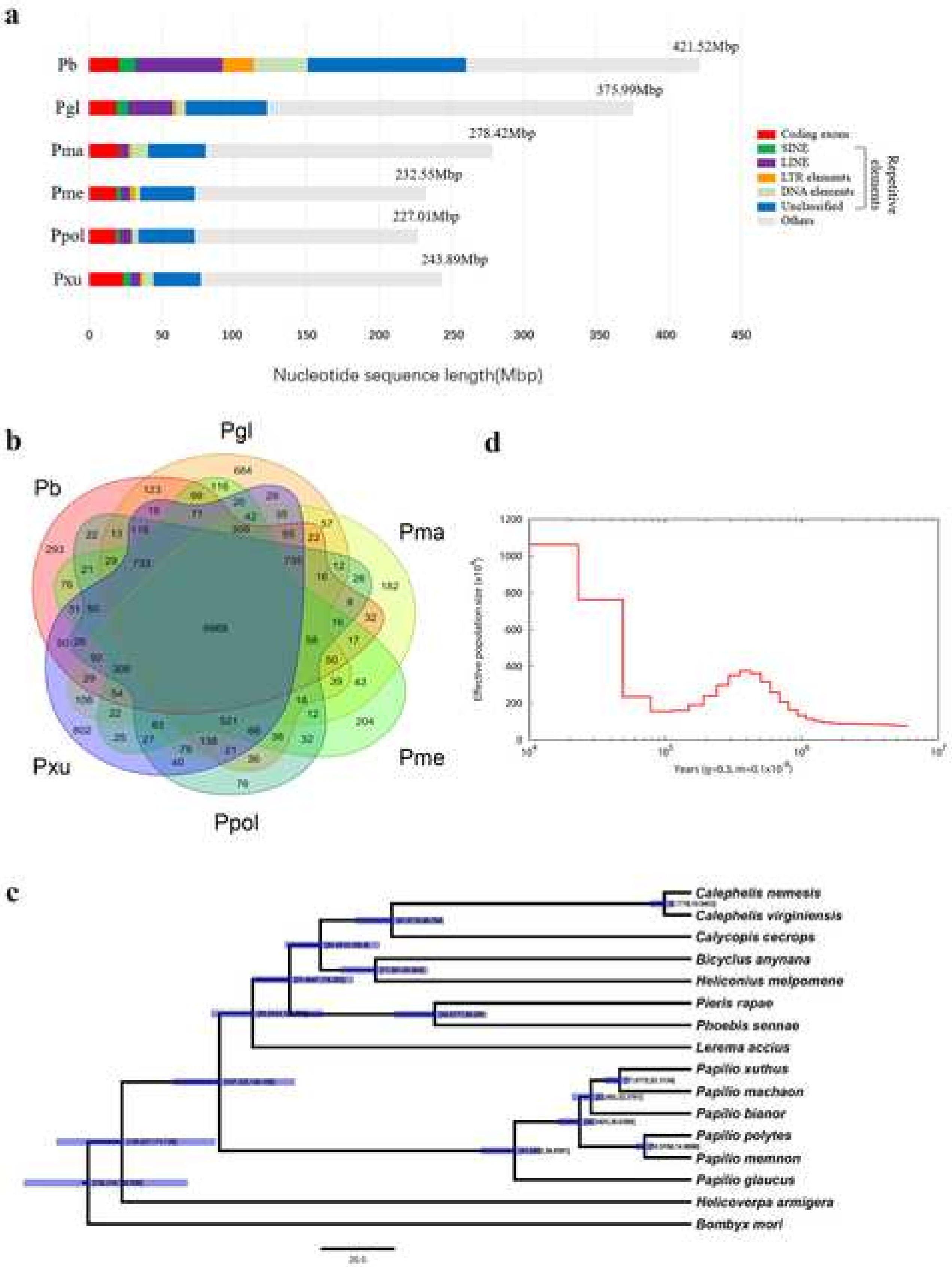

Click here to access/download
Supplementary Material
Pb-Genome_submitted version_Suplementary files_20190409.docx

Supplementary Figure 1

Click here to access/download
Supplementary Material
Figure S1.jpg

Supplementary Figure 2

Click here to access/download
Supplementary Material
Figure S2.jpg

Personal Cover
Click here to download Personal Cover Pb-Genome_submitted version_cover letter_20190409.docx

Dear Editors of *GigaScience*,

We would like to submit our manuscript entitled "**Chromosomal-level reference genome of Chinese peacock butterfly (*Papilio bianor*) based on third-generation DNA sequencing and Hi-C analysis**" for your consideration as a **Data Note** in "***GigaScience***". This submission includes ~6,200 words in main text, 1 table and 2 figures in total, Supplementary Tables 1-13 and Supplementary Figures 1-2. We declare that all the content of the manuscript has not published or submitted for publication elsewhere. We acknowledge that all authors have contributed significantly and that all authors are in agreement with the content of the manuscript.

Butterflies have been favored by naturalists for centuries, and the study of butterflies has been an integral part of ecology and evolution ever since Darwin proposed his theory of natural selection in 1859. Back in 1864, H. W. Bates, the famous inventor of mimicry theory, predicted that *"the study of butterflies…will someday be valued as one of the most important branches of Biological science."*. Chinese peacock butterfly *Papilio bianor* is one of ideal model organisms in genetics, evolutionary biology and phylogeographic researches due to its special features such as easy breeding, different kinds of wing color, and widely geographic distribution. A high-quality chromosome-level reference genome of *P. bianor* is very important for investigating iridescent color evolution, phylogeography, and evolution of swallowtail butterflies.

In this study, we assembled the chromosome-level genome of the high heterozygous Chinese peacock butterfly (*P. bianor*) genome (1.81 %) using combined Illumina, PacBio, and Hi-C technologies. The final assembly is 402.00 Mb on 30 chromosomes (29 autosomes and 1 sex chromosomes W) with the contig and scaffold N50 as 5.50 Mb and 12.51 Mb, respectively. And the maximum length of contig and scaffold as 15.05 Mb and 17.37 Mb, respectively. The genomic resources generated in this study lay the foundation for exploring genetic basis of special biological features of the Chinese peacock butterfly, and also provide a useful datasource for comparative genomics and phylogenomics among butterflies and moths.

These findings is expected to be of interest to a broad audience of entomologist and ecologists, especially the researchers working on butterflies. As the international journal focusing on 'Big data' research from the life and biomedical sciences,

GigaScience represents the ideal platform for us to share our results with the international research community. We look forward to hearing from you at your earliest convenience.

Thank you for your consideration.

Sincerely,

Xueyan Li, Ph.D
State Key Laboratory of Genetic Resources and Evolution
Kunming Institute of Zoology, Chinese Academy of Sciences (CAS), Kunming, Yunnan 650223, China
Email: lixy@mail.kiz.ac.cn, Tel: 86-871-68125339, Fax: 86-871-68125338

Wen Wang, Ph.D
State Key Laboratory of Genetic Resourses and Evolution
Kunming Institute of Zoology, Chinese Academy of Sciences (CAS), Kunming, Yunnan 650223, China
Center for Ecological and Environmental Sciences, Northwestern Polytechnical University, Xi’an 710072, China
Email: wwang@mail.kiz.ac.cn
