# Supplementary material for: Chromosomal-level reference genome of Chinese peacock butterfly (Papilio bianor) based on third-generation DNA sequencing and Hi-C analysis
Source: Gigascience. 2019 Nov 4;8(11):giz128. doi: 10.1093/gigascience/giz128 (PMC6827417; doi:10.1093/gigascience/giz128)
Supplement: giz128_GIGA-D-19-00120_Revision_1 [file giz128_giga-d-19-00120_revision_1.pdf]

GigaScience

Chromosomal-level reference genome of Chinese peacock butterfly (Papilio bianor) based on third-generation DNA sequencing and Hi-C analysis

--Manuscript Draft--

**Manuscript Number:**	GIGA-D-19-00120R1
**Full Title:**	Chromosomal-level reference genome of Chinese peacock butterfly (Papilio bianor) based on third-generation DNA sequencing and Hi-C analysis
**Article Type:**	Data Note
**Funding Information:**	National Natural Science Foundation of China (31621062) — Dr. Wen Wang<br>Chinese Academy of Sciences (XDB13000000) — Dr. Wen Wang<br>CAS "Light of West China" — Dr. Xueyan Li
**Abstract:**	Background<br>Papilio bianor Cramer, 1777 (i.e. Chinese peacock) (Insecta, Lepidoptera, Papilionidae) is a widely distributed swallowtail butterfly with a large number of geographic populations from the Southeast of Russia to China, Japan, India, Vietnam, Myanmar and Thailand. Its wing color consists of both pigmentary colored scales (black, reddish) and structural colored scales (iridescent blue or green dust). A high-quality reference genome of P. bianor is thus important for investigating iridescent color evolution, phylogeography, and evolution of swallowtail butterflies.<br>Findings Here, we obtained a chromosome-level de novo genome assembly of the high heterozygous Chinese peacock (Papilio bianor) (1.81%) using long Pacific Biosciences (PacBio) sequencing reads (43.19 Gb) and high-throughput chromosome conformation capture (Hi-C) technology. The final assembly is 402.00 Mb on 30 chromosomes (29 autosomes and 1 sex chromosomes W) with 5.50 Mb contig N50 and 12.51 Mb scaffold N50. Totally 15,375 protein-coding genes and 222.29 Mb (55.30%) of repetitive sequences were identified. The phylogenetic trees of representative species of butterflies and moths constructed using one to one single-copy orthologous genes indicate that the Chinese peacock was separated from a common ancestor of swallowtails about 23.69-36.04 million years ago (mya). Demographic history inferred using the Pairwise Sequentially Markovian Coalescence (PSMC) analysis suggested that the population expansion of this species from the last interglacial period to the last glacial maximum possibly resulted from its decreased natural enemies and its adaptation to climate diversity during glacial period.<br>Conclusions<br>We present a high-quality chromosome-level reference genome of the Chinese peacock (Papilio bianor) using long-read single-molecule sequencing and Hi-C-based chromatin interaction maps. Our results lay the foundation for exploring genetic basis of special biological features of the Chinese peacock butterfly, and also provide a useful datasource for comparative genomics and phylogenomics among butterflies and moths.
**Corresponding Author:**	Xueyan Li, Ph.D<br><br>CHINA
**Corresponding Author Secondary Information:**	
**Corresponding Author's Institution:**	
**Corresponding Author's Secondary Institution:**	
**First Author:**	Sihan Lu, Ph.D
**First Author Secondary Information:**	
**Order of Authors:**	Sihan Lu, Ph.D

Powered by Editorial Manager® and ProduXion Manager® from Aries Systems Corporation

	Xueyan Li, Ph.D
	Jie Yang
	Xuelei Dai
	Feiang Xie
	Jinwu He
	Zhiwei Dong
	Junlai Mao
	Guichun Liu
	Zhou Chang
	Ruoping Zhao
	Wenting Wan
	Ru Zhang
	Yuan Li
	Wen Wang
**Order of Authors Secondary Information:**	
**Response to Reviewers:**	Dear Prof. Zhou,<br><br>We are submitting our revised manuscript entitled "Chromosomal-level reference genome of Chinese peacock butterfly (Papilio bianor) based on third-generation DNA sequencing and Hi-C analysis" (Manuscript ID: GIGA-D-19-00120). We appreciate all the positive comments and valuable suggestions from you and reviewers.<br><br>Based on reviewers' comments and editor's instructions, we have endeavored to improve our manuscript mainly as follows: (1) we have performed reanalysis or extension of the methods used for genome assembly and annotation, evolutionary analyses including syntenic relationships between gemomes of P. bianor and P. xuthus, phylogenetic relationships of Papilionoidea and demographic history of P. bianor as reviewer#1 suggested and added methodological details in revised MS; (2) we have confirmed the high repeat content in P. bianor genome using the methods in the reference recommended by reviewer#2; (3) we have checked the whole manuscript carefully, and improved the language and writing as reviewer#2 suggested; (4) we have added the RRID (Research Resource Identification Initiative ID) number in our revised manuscript as editor suggested. We also carefully checked the entire manuscript and corrected any errors. All the changes were highlighted in yellow.<br><br>We provide detailed point-by-point responses to the reviewers' comments as follows .<br><br>Thank you very much for your consideration.<br><br>With best regards,<br>Sincerely yours<br><br>Xueyan Li, Ph.D<br>State Key Laboratory of Genetic Resources and Evolution<br>Kunming Institute of Zoology, Chinese Academy of Sciences (CAS), Kunming, Yunnan 650223, China<br>Email: lixy@mail.kiz.ac.cn, Tel: 86-871-68125339, Fax: 86-871-68125338<br><br>Wen Wang, Ph.D<br>State Key Laboratory of Genetic Resources and Evolution<br>Kunming Institute of Zoology, Chinese Academy of Sciences (CAS), Kunming, Yunnan 650223, China<br>Center for Ecological and Environmental Sciences, Northwestern Polytechnical University, Xi'an 710072, China<br>Email: wwang@mail.kiz.ac.cn

Powered by Editorial Manager® and ProduXion Manager® from Aries Systems Corporation

Point-by-Point Responses

Revierer #1: Dear Dr. Hongling Zhou,
Sihan Lu, Jie Yang, Xuelei Dai, Feiang Xie, et al assembled a high-quality reference genome of the Chinese Peacock Swallowtail, Papilio bianor. It was then used to carry out phylogenetic and demographic inference along with molecular dating.

In my opinion this paper is suitable for publication in GigaScience and will be of broad interest as: 1) Chromosome level assembly of a heterozygous insect genome is carried out, 2) shows an interesting expansion of repetitive elements and gene families in the P. bianor lineage, and 3) provides a good resource for phylogenetics and comparative genomics within the Lepidoptera. The manuscript is well laid out, however, proper expression is lacking in many places and the manuscript would be improved greatly if copy-edited.
R: We thank the Reviewer for the positive comments on this work.

The methods used for the assembly, annotation and evolutionary analyses are suitable for the results presented, but are generally too vague to allow for one to repeat the analysis.

I have some concerns with the manuscript that should be addressed before the paper would be suitable for publication. Most concerns relate to the text itself but some may involve reanalysis or extension of the analyses performed. Please see attached specific comments.
R: We thank the reviewer for suggestive and valuable comments. As suggested, we performed reanalysis or extension of analyses including syntenic relationships between gemomes of P. bianor and P. xuthus, phylogenetic relationships of Papilionoidea and demographic history of P. bianor.

1) Abstract: "The final assembly is 402.00 Mb on 30 chromosomes (29 autosomes and 1 sex chromosomes W)".
As males were sequenced, only Z chromosome sequence is expected as female Lepidopterans are ZW.
R: Thank you for the comment. We have corrected it in the revised manuscript.

2) Line 159 - 162: Finally, we obtained the chromosomal-level high-quality assembly of P. bianor with total length of ~402.00 161 Mb and the longest scaffold N50 (12.51 M) among the published butterfly genomes (Table 1 & Table S4).
It is unclear to me where the value of 402Mb comes from. Table S3 reports 381.5Mb and 421.5Mb as the chromosome-assigned and total scaffold length respectively. Table S4 reports 421,524,737bp with an N50 of 13,120,256 whereas Table 1 reports a genome size of 402 Mb with an N50 of 12813kb. Was ~20Mb of the assembly removed during the reduction of heterozygous contigs?
R: Thank you for these comments. In previous MS, we made unit conversion of genome size based on the unit conversion of storage capacity (1 Tb=1024 Gb, 1 Gb=1024 Mb, 1 Mb=1024 Kb, 1 Kb=1024 bp), resulting in 421,524,737 bp to 402 Mb (421,524,737 bp/1024 ≈ 411,645 Kb, 411,645 Kb/1024 ≈ 402 Mb), and N50 of 13,120,256 bp to 12813 Kb. After careful checking, we agree decimal system (i.e. 1 kb = 1000 bp, 1 Mb = 1000 kb) should be adopted in measuring genome size. Thus, we have corrected our description in the revised MS.

3) Line 174 – 176: Thirdly, we compared syntenic relationships between gemomes of P. bianor and P. xuthus (Fig. 1c) and found that 94.96% of the P. bianor assembled genome sequences can be aligned (1:1) to the P. xuthus reference genome.
As P. bianor has a genome ~2x the size of P. xuthus ~95% cannot be aligned 1:1.
R: Thank you for the valuable comment. It was our mistake to generate the illogical ratio in the previous MS, because we included the whole length of those chromosomes in case they can be aligned 1:1 with high-confidence (-m 0.01) (i.e. (aligned regions + unaligned regions) of a chromosome). Here, we performed the reanalysis as the followings: we aligned P. bianor base-pair to at most one P. xuthus base-pair, and then swapped the sequences and ran last-split with high-confidence (-m 0.01) to get 1-to-1 alignments (that is to say, only aligned regions were included). Finally, we found that

Powered by Editorial Manager® and ProduXion Manager® from Aries Systems Corporation

61,082,412 bp of the P. bianor assembled genome sequences can be aligned (1:1) to the P. xuthus reference genome. We have modified it to a more appropriate way. We have made the following changes in the text accordingly: “Thirdly, we compared syntenic relationships of P. bianor genome with that of P. xuthus, which is the only chromosomal-level assembly (by linkage map methods) [9] among all Papilio reference genomes and thus was considered to the best-assembled one (Fig. 1c). We found that 61,082,412 bp of the P. bianor assembled genome sequences can be aligned (1:1) with high-confidence (-m 0.01) to the P. xuthus reference genome.” (Page 9, Line 183-188)

4) Line 251: 1378 one to one single orthologs
It is not clear how these were identified, were they from the BUSCO analysis?
R: Thank you for the comment. We have added related methods in our revised manuscript to make it more explicit: “1378 one-to-one single copy orthologs which contain only one protein for each species were collected and clustered by OrthoMCL (version 2.0.9; OrthoMCL DB: Ortholog Groups of Protein Sequences, RRID:SCR_007839) [83] from these 16 species and their nucleic acid sequences were aligned using PRANK (version 3.8.31; PRANK, RRID:SCR_017228) [85].” (Page 12-13, Line 276-280)

5) Line 253 – 254: construct the phylogenetic trees using RAxML (version 7.2.8; RAxML, RRID:SCR 006086) [83] by choosing the GTR+G+I model.
Was a concatenated alignment of all the genes used or were partitions set? If no partitions were set it would be worth setting gene partitions or using PartitionFinder to determine partitions. Alternatively, the authors should consider constructing gene trees for each of the single copy orthologs and inferring the species tree from these using an ILS aware algorithm such as ASTRAL (https://github.com/smirarab/ASTRAL). This would remove the need for partitioning and reanalysis of the entire supermatrix using RAxML.
R: Thank you for valuable suggestion. We have added the following sentences to make this part more readable: “Furthermore, in order to make our result more clarified, we also have constructed the gene trees for each of the orthologs with RAxML software (version 7.2.8; RAxML, RRID:SCR_006086) [84] by choosing the GTR+G+I model and inferred the species tree from these with ASTRAL software (version 5.6.3) [87] (Fig. S4). As expected, the results are consistent with each other.” (Page 13, Line 282-287).

We also give some explanations here.
As shown in Figure 2c, a concatenated alignment of all the genes (i.e. 1378 one to one single orthologs previously identified) was used for constructing the phylogenetic trees with RAxML software (version 7.2.8; RAxML, RRID:SCR_006086) [1] by choosing the GTR+G+I model (more details see discussion of Fig. 2c in our manuscript). Furthermore, in order to make our manuscript more clarified, we also have constructed the gene trees for each of the 1378 one to one single copy orthologs with RAxML software (version 7.2.8; RAxML, RRID:SCR_006086) [1] by choosing the GTR+G+I model and inferred the species tree from these with ASTRAL software (version 5.6.3) [2]. The related tree was added as Fig. S6. As expected, both results are consistent with each other.

Figure 2c. Maximum Likelihood (ML) phylogenetic tree of Papilionoidea constructed by the concatenated alignment of 1378 one to one single copy orthologue genes. The numbers in the square brackets on the nodes are the 95% confidence intervals of divergence time. The red dots are fossil evidence downloaded from the TIMETREE website and the black dots are inferred time obtained from the TIMETREE website. Both of them were used to calibrate divergent time.

Figure S6. Maximum Likelihood (ML) phylogenetic tree of Papilionoidea constructed by the merging each of the single copy orthologs.

6) Line 254 – 256: The phylogeny was further analyzed by PAML MCMCtree (version 4.5; PAML, RRID:SCR 014932) program [84], and calibrated with published timings for the divergence of difference species [85].
This is insufficient information to reproduce the analysis. Include clock type,

substitution model, and if default parameters were used for alpha rate, number of categories, and other priors. Also report how many chains were run and if the model reached convergence.
Were all internal nodes informed using TimeTree? This may cause over parameterization, i.e. Do calibration times match well with the estimated split times of your nodes?
It would also be good to include the dates used as TimeTree ranges for calibration nodes may be updated in the future.
R: Thank you for the insightful comments. We have added the following sentence accordingly: “To further investigate the divergence time of these species, the phylogeny was further analyzed by MCMCtree in PAML (version 4.5; PAML, RRID:SCR_014932) software [88] with default parameters, and calibrated with published divergent times of some nodes estimated from fossil evidence or obtained from TIMETREE website [89].” (Page 13, Line 287-291). We also marked the nodes for calibrating the time in the revised figure (Fig. 2c), and improved the legend of Fig. 2c accordingly (Line 636-641).

We give some explanations here.
All related parameters were adopted the default parameters, including the clock type (correlated rates), substitution model (HKY85), alpha rate (0.5), number of categories, and other priors. 100,500,000 (burnin=500,000, sampfreq=5,000, nsample=20,000) chains were run and the model reached coveragence in the analysis.
The red dots are fossil evidence downloaded from the TIMETREE website and the black dots are inferred time obtained from the TIMETREE website. Both of them were used to calibrate the results (as shown in Fig. 2c & Fig. R1). The fossil evidences reveal that Bombyx mori diverged from the common ancestor of Helocoverpa armigera and Papilionoidea 52-177 million years ago (mya) while the split of Papilionoidea was 76-146 mya. The inferred times obtained from the TIMETREE website show that the divergence times between Bicyclus anynana and Heliconius melpomene, between Pieris rapae and Phoebis sennae, between Papilio polytes and Papilio memnon, between Papilio xuthus and Papilio machaon are 68-100 mya, 50-82 mya, 9.7-15.1 mya, and 17.6-33.6 mya, respectively. The inferred times also indicate that Papilio glaucus diverged with the common ancestor of other species in Papilio 18-61 million years ago while Pieridae diverged from the common ancestor of Nymphalidae, Lycaenidae, and Riodinidae 79-118 mya.
The results show that the estimated split times of these two results (inferred using orthologous genes and inferred using four-fold degenerate sites) are similar, which are consistent with the timetree results.

Figure 2c. Maximum Likelihood (ML) phylogenetic tree of Papilionoidea constructed by the concatenated alignment of 1378 one to one single copy orthologue genes. The numbers in the square brackets on the nodes are the 95% confidence intervals of divergence time. The red dots are fossil evidence downloaded from the TIMETREE website and the black dots are inferred time obtained from the TIMETREE website. Both of them were used to calibrate divergent time.

Figure R1. Maximum Likelihood (ML) phylogenetic tree of Papilionoidea constructed by the concatenated alignment of four-fold degenerate sites (4dTV). The numbers in the square brackets on the nodes are the 95% confidence intervals of divergence time. The red dots are fossil evidence downloaded from the TIMETREE website and the black dots are inferred time obtained from the TIMETREE website. Both of them were used to calibrate the results.

7) Line 258 – 259: all Papilio species is a monophyly, and diverged from other butterflies approximately 41.07-56.86 mya
Consider rephrasing to: “all species of Papilio analysed are in monophyly with a crown node age of approximately 41.07-56.86 mya”
R: Thanks for Reviewer’s suggestion. We improved as suggested. (Page 13, Line 293/4)

8) Line 264 – 267: We also inferred demographic histories of P. bianor by SNP calling of Illumina short reads against assembled genome using the Pairwise Sequentially Markovian Coalescence (PSMC) analysis [89] (0.1 × 10-8 mutations per site per

generation calculated by r8s [90]; three or four generations per year [47]).
Specify what tools were used for variant calling, was SAMtools mpileup used (as recommended on Heng Li's github https://github.com/lh3/psmc)?
Please also specify time intervals used and I recommend bootstrapping the analysis, a detailed example of how to do this can be found on Heng Li's github page for PSMC. Using different time intervals may also increase resolution in the recent history.
R: Thank you for the valuable suggestions. As suggested, we improve this part as the followings: "We also inferred the demographic histories of P. bianor applying the Pairwise Sequentially Markovian Coalescence (PSMC; PSMC, RRID:SCR_017229; with -p 64*1 parameters) analysis [93] (3.56 × 10-3 mutations per site per generation calculated by r8s [94]; three or four generations per year [48]), which was carried out by mapping Illumina short reads to the assembled genome with BWA (version 0.7.12-r1039; BWA, RRID:SCR_010910) [58] and calling variants with SAMtools (version 1.3.1; SAMTOOLS, RRID:SCR_002105; with samtools mepileup -C50 –uf parameters) [95]." (Page 13-14, Line 299-306).

Here, we also provide some explanation.
We used the BWA-mem to map short reads to the reference genome, and then called variants to get the bcf format file using samtools mepileup (samtools mepileup -C50 -uf). Bcftools (bcftools call –c) was used to convert the bcf format into the vcf format for the further PSMC analysis.
We have specified the time intervals and have changed the time intervals from "-p 28*2+3+5" to "-p 64*1" to increase resolution in the recent history (as shown in Fig. 2d). We also have run 100 bootstrap replicates using PSMC software to test the robust variations (Fig. 2d).

Figure 2d. The dynamic changes of the effective population size are plotted using PSMC software, with 100 bootstrap replicates to test the robust variations. The parameter "g" represents the generation time in years, and the parameter "μ" means the per generation mutation rate.

Since the species is three or four generations a year, we have chosen two extreme values (g=0.25, Fig. R2; g=0.4, Fig. R3) to ensure the availability of information.

Figure R2. Demographic history of P. bianor are plotted using PSMC software. The parameter "g=0.25" indicates generation time in years, and "μ=0.89*10-9" indicates genomic substitution rate.

Figure R3. Demographic history of P. bianor are plotted using PSMC software. The parameter "g=0.4" indicates generation time in years, and "μ=0.14*10-8" indicates genomic substitution rate.

Based on the above results, we have reedited Fig. 2d accordingly. We also have changed the wrong date "0.1 × 10-8 mutations" to the right one "3.56 × 10-3 mutations" in the revised manuscript because we confused the result calculated by r8s with parameter "μ" in our previous manuscript.

9) Line 268 -270: Our result suggested that the effective population size increased significantly from the last interglacial period (LIG, approximately 0.1 million years before present) to its maximum at the last glacial maximum (LGM, approximately 0.01 million years before present).
Does this match the demographic history of any other Papilio species?
R: Thank you for this important comment. Our result on the trend of the demographic history of P. bianor is in good agreement with those of other five Papilio species (P. ambrax, P. phestus, P. polytes alphenor, P. protenor, and P. polytes polytes) reported previously [3] (Fig. R4). We have changed the sentence and cited the corresponding reference accordingly as the followings: "Our result suggested that the effective population size increased significantly corresponded to the transition phase from the last interglacial period (LIG, approximately 0.14-0.12 million years before present) to the last glacial maximum (LGM, approximately 0.021-0.018 million years before present) (Fig. 2d), which is in good agreement with other five Papilio species [96]." (Page 14, Line 306-310)

Powered by Editorial Manager® and ProduXion Manager® from Aries Systems Corporation

Figure R4. Historical effective population sizes were inferred from eight individual genomes using PSMC, assuming a mutation rate of μ=3 × 10-9 and an average generation time of g=0.25 year. (quoted from: Zhang W et al (2017) doi: Artn 1269 10.1038/S41467-017-01370-1)

10) Other comments:
Heterozygosity is relatively high (1.8%) but samples were collected from the wild. Many genome studies use inbred strains to reduce polymorphism. Could there be any bias in these statistics?
Data source (references) for Table 1.
R: Thanks you for the insightful comments. Yes, as Reviewer pointed out, wild animals (especially insect) usually exhibit high heterozygosity. To cultivate inbred strains is a reliable method to reduce polymorphism, which, however, is not feasible in all cases, especially for wild insects. Both above mentioned points are just the bottlenecks to impede the dissection of reference genomes from more insects, especially only using Next-generation sequencing. PacBio SMRT sequencing technology break the bottlenecks for dissecting reference genomes with high heterozygosity. In this work, heterozygosity was calculated based on k-mer distribution analysis, which is a commonly used method to estimate heterozygosity of genomes (e.g. P. machaon and P. xuthus in butterflies [4]). As suggested, to avoid confusion, we have removed the percentage heterozygosity in the Abstract. We also have provided the algorithm for different species in the interpretation of Table 1: “The heterozygosity of P. bianor, P. machaon and P. xuthus were calculated based on k-mer distribution analysis. The heterozygosity values of others (P. glaucus, A. lyciades, L. accius, M. ursus violae, P. rapae, P. sennae, D. plexippus, C. nemesis, C. virginiensis, C. cecrops) were estimated using the Genome Analysis Toolkit (GATK).” (Line 619-621)
Reviewer #2: Review of Lu et al submission: Chromosomal-level reference genome of Chinese peacock butterfly (Papilio bianor) based on third-generation DNA sequencing and Hi-C analysis

Lu et al describe the construction of a chromosomal-level reference genome assembly for Papilio bianor and set this in the context of other butterfly genome assemblies. This manuscript was a pleasure to review. The genome assembly project has been carefully conducted and a good level of supporting data is included. The manuscript requires some editing for language (almost all of my minor comments address language issues) but the intentions and the analyses that have been carried out are nevertheless clear and this assembly will be an important resource for the butterfly research community and beyond.
R: We thank reviewer for positive comments on our work. We also appreciate Reviewer for careful and kind checking on our MS and all valuable comments. As suggested, we improved this MS.

Major comments
Regarding repeat content, to what extent does your observation of such extremely high repeat content reflect your methods of ascertainment and methods used in other studies? For example, see Platt et al (2016) doi: 10.1093/gbe/evw009. How confident are you that this repeat expansion is genuine and not (partly or wholly) an artefact of ascertainment? Whilst this expansion sits neatly with the increased genome size, I would like to see some discussion of the limitations of making these broad comparisons between assemblies generated and analysed with different methodologies.
R: Thanks you for the valuable comments. As suggested, we used the method in the recommended reference to annotate the repetitive sequences of P. bianor genome. And the related repetitive sequences content is consistent with the previously annotated. We have added the following sentence accordingly: “To confirm the reliability of high repetitive sequences in P. bianor, which is much higher than those (<35%) of other butterflies (Table 1), we also used other de novo annotation method reported by Platt II et al. and Lavoie et al [69, 70] to annotate the repetitive sequences of P. bianor genome. Based on this method, P. bianor genome possesses 53% repeat elements, similar to the previous annotated results (Table 1 & Table S8), thus confirming high repetitive sequences in P. bianor genome.” (Page 10, Line 214-220).

Here, we also provide some explanation.
In this research, the repeat content we calculated was up to 55%, in which 51% was

from the de novo repeatmasker (Table S8). The library of de novo repeatmasker was produced by RepeatModeler software (version 1.0.4; RepeatModeler, RRID:SCR_015027) [5]. According to the reviewer's advice, we have referred to the method reported by Lavoie et al (2013) doi: 10.1186/1759-8753-4-21 [6], which was also recommended by Platt et al (2016) doi: 10.1093/gbe/evw009 [7]. In details, firstly, we blasted (-evalue 1e-5) the RepeatModeler outfile to the whole genome and then extended the resulting sequence by flanking 1000 bp to get the potential repeat seed sequence. Secondly, we used MUSCLE (version 3.8.31; MUSCLE, RRID:SCR_011812) [8] to align the extracted sequence, TRIMAL (version 1.2rev59; with -gt 0.6 -cons 60) [9] and EMBOSS (version 6.6.0.0; EMBOSS, RRID:SCR_008493; with cons -identity 3) [10] to generate the 50% majority rule consensus sequence. Finally, the consensus sequence was used as the new library to the repeatmasker program. In the modified method, we got 53% repeat elements, of which 48% was annotated from the consensus library. So the results obtained were not significantly different from the previous results. In addition, the result of k-mer evaluation of repeat content was 58.3%, which confirmed the high repeat ratio in this species.

In a similar vein, it would also be interesting to see some discussion of how data from different wild-caught individuals was combined to generate the assembly draft. Especially in the context of such high heterozygosity this has the potential to cause difficulties in assembly and might inflate estimates of genome size. This was alluded to in 141-163, when criteria for merging contigs were set out. I think further discussion of whether this had an impact on the assembly process would be of general interest to the GigaScience readership.
R: Thank you for the important comment. Yes, this is a topic of general interest. The amount of DNA extracted from single individual of especially many insects and even other invertebrates is usually not enough to satisfy such different types of sequencing as genome survey (Illumina), PacBio, and Hi-C, which, if possibly, is the best ideal case. We were also asked for our experience on this topic. Following our experience, it is acceptable to use different wild individuals. In our current study, one male adult was sued for Illumina sequencing, another male adult for PacBio sequencing, and a sample mixed from whole body of two male larval individuals (the fifth instar) was used for Hi-C sequencing. The results on quality evaluation of our assembled genomes (original lines 166-178; Revised lines 172-191) provided support to such a method that different individuals were used for different sequencing for de novo genome assembling. As suggested by reviewer, aiming to make this topic more explicit, we also added the coverage distribution of Illumina reads/PacBio reads mapping to the assembled genomes (Fig. S3, Fig. S4), which show a near normal distribution and thus indicate few heterozygous regions in assembled genome. Further, we improved the expression "All these results suggest that the assembled P. bianor genome is of high quality (including completeness, base level contiguity and accuracy) (Table 1)." to "All these results suggest that P. bianor genome, which is assembled based on PacBio reads, Illumina reads and Hi-C data sequenced from different wild individuals, is of high quality (including completeness, base level contiguity and accuracy) (Table 1)". (Page 9, Line 188-191)

Figure S3. The coverage distribution of Illumina reads mapping to Papilio bianor genome. The histogram follows a normal distribution, indicating few heterozygous regions in assembled genome.

Figure S4. The coverage distribution of PacBio reads mapping to Papilio bianor genome. The histogram follows a normal distribution, indicating few heterozygous regions in assembled genome.

It would be interesting to know why the authors selected P. xuthus for comparisons. Is there a particular biological justification? Table 1 suggests it may be considered the best-assembled of the other Papilio genomes. Perhaps include a statement in the text explaining why this pairwise comparison was selected?
R: Thanks you for the valuable suggestion. As suggested, we improved the description in the revised manuscript as the followings: "Thirdly, we compared syntenic relationships of P. bianor genome with that of P. xuthus, which is the only

Powered by Editorial Manager® and ProduXion Manager® from Aries Systems Corporation

	chromosomal-level assembly (by linkage map methods) [9] among all Papilio reference genomes and thus was considered to the best-assembled one (Fig. 1c). We found that 61,082,412 bp of the P. bianor assembled genome can be aligned (1:1) with high-confidence (-m 0.01) to the P. xuthus reference genome." (Page 9, Line 183-188)<br><br>Fig1b is an excellent overview but the detail of the heatmap is hard to see on this scale. Perhaps consider releasing per-chromosome plots as part of supplementary material.<br>R: Thank you. We have added the per-chromosome plots as Fig. S2 in the supplementary material. (Line 660/1)<br><br>Figure S2. Heatmap of per-chromosomal interactions. Each scaffold is framed with green block.<br><br>In general, I dislike the statements of priority (e.g. in line 103- this is the 4th chromosomal-level reference genome) as these are prone to become quickly out of date and also are somewhat irrelevant.<br>R: Thank you. As suggested, we have removed the information about priority in our manuscript. (Page 6, Line 105/6)<br><br>Minor comments:<br>1) 26: change "i.e. Chinese peacock" to "commonly known as the Chinese peacock butterfly"<br>R: Thank you. We modified it as suggested. (Page 2, Line 27/8)<br><br>2) 28: southeast<br>R: Thank you. We corrected it as suggested. (Page 2, Line 30)<br><br>3) 31: consider changing "is thus important" to "is an important foundation for"<br>R: Thank you. We improved it as suggested. (Page 2, Line 33)<br><br>4) 32: and the evolution of<br>R: Thank you. We corrected it as suggested. (Page 2, Line 34)<br><br>5) 33: highly<br>R: Thank you. We corrected it as suggested. (Page 2, Line 35)<br><br>6) 34: consider removing the percentage heterozygosity from the abstract as it needs further explanation.<br>R: Thank you. As suggested, we have removed the "percentage heterozygosity" from the abstract. (Revisions is made in Page 2, Line 36 in the revised manuscript)<br><br>7) 36/7: (29 autosomes and 1 W sex chromosome)<br>R: Thank you. We corrected it as suggested. (Page 2, Line 38)<br><br>8) 37: In total, 15,375…<br>R: Thank you. We improved it as suggested. (Page 2, Line 39)<br><br>9) 38: Phylogenetic analyses indicated that…<br>R: Thank you. We improved it as suggested. (Page 2, Line 40)<br><br>10) 43: What exactly do you mean by climate diversity? Climate change?<br>R: We thank the reviewer for bringing out this valuable point. Yes, what we want to expreess is that population of this species has expanded owing to the decrease of its natural enemies and its adaptation to climate change. We changed "climate diversity" to "climate change". (Page 2, Line 44)<br><br>11) 46: Our results lay the foundation for exploring the genetic basis…<br>R: Thank you. We corrected it as suggested. (Page 2, Line 47)<br><br>12) 54: I am not keen on this statement "Butterflies are one of most charming animals". I would prefer "Butterflies are widely considered….". In a similar vein, I am not convinced by the use of "inventor" to describe Bate's contribution to mimicry theory (line 61).

R: Thank you for the good suggestions. As suggested, we have changed “Butterflies are one of most charming animals” (line 55) to “Butterflies are widely considered as one of most charming animals”; we have changed the word “inventor” (line 62) to word “pioneer”.

13) 58: change to “have been regarded as important model organisms in …”
R: Thank you. We corrected it as suggested. (Page 4, Line 59)

14) 63: I think this sentence needs a bit more development. Presumably the argument is that technology development has rendered possible direct analysis (and even manipulation) of the genomes of individuals sampled from natural habitats without the need to inbreed to reduce heterozygosity or to develop lab lines etc.
R: Thank you for the suggestive comments. We have rewritten the following sentences accordingly: “With the feasibility to dissect the heterozygous genomes of such wild insects like butterflies and to perform genetic manipulation on them [9-11], butterflies have been becoming a promising system to explore the genetics, evolution, morphological diversification and speciation.” Changed to: “With the technology development, it is possible to conduct direct analysis (and even manipulation) of the genomes of individuals sampled from natural habitats without the need of inbreeding to reduce heterozygosity or to develop lab lines [9-11], so butterflies have been becoming a promising system to explore the genetics, evolution, morphological diversification and speciation.” (Page 4, Line 64-68)

15) 69: have had reference genomes assembled, at the time of writing. [care needed here as this is an active area- there are many others in the process of being assembled]
R: Thank you for the suggestive comments. We have added the statistical date (until May 1, 2019) in the revised manuscript. (Page 4, Line 72)

16) 71: Nymphalidae (or nymphalids)
R: Thank you for careful checking. We changed “nymphids” to “nymphalids”. (Page 4, Line 73)

17) 73: linkage map methods
R: Thank you. We corrected it as suggested. (Page 4, Line 75)

18) 75: “...which may often result from …”
R: Thank you. We corrected it as suggested. (Page 4, Line 77)

19) 76-77 “will provide a unique opportunity to promote the evolutionary biological studies on the famous butterfly system.” This sentence remains a bit unclear- what exactly do you mean by “famous butterfly system”?
R: Thank you for the suggestive comments. We changed “famous butterfly system” to “butterflies as an important model system”. (Page 5, Line 79)

20) 80: “paved the way for the dissection of…”
R: Thank you. We improved it as suggested. (Page 5, Line 81/2)

21) 81: high-throughput
R: Thank you. We corrected it as suggested. (Page 5, Line 83)

22) 82: “which was developed to identify…. and is now also ….”
R: Thank you. We corrected it as suggested. (Page 5, Line 84/5)

23) 87: has been reported
R: Thank you. We corrected is as suggested. (Page 5, Line 89-90)

24) 90: Known as the Chinese.. or the Chinese peacock,”
R: Thank you. We corrected is as suggested. (Page 5, Line 93)

25) 92: southeast
R: Thank you. We corrected is as suggested. (Page 5, Line 95)

26) 93: Its larvae mainly feed on plants of the Rutaceae family, such as … and it

completes its life cycle in 40-50 days.
R: Thank you. We corrected is as suggested. (Page 5, Line 96-98)

27) 96: perhaps “structurally-coloured scales”?
R: Thank you. We corrected is as suggested. (Page 5, Line 99)

28) 99: prothoracicotropic
R: Thank you. We corrected is as suggested. (Page 5, Line 102)

29) 100: species delimitation
R: Thank you. We corrected is as suggested. (Page 6, Line 103)

30) 101: PacBio SMRT
R: Thank you. We corrected is as suggested.(Page 6, Line 104)

31) 134: A sample. Does the reference in 35 also detail DNA extraction method? If not please include details.
R: Thank you. We corrected “The sample” to “A sample”. Yes, the changed reference in 36 details the DNA extraction method. (Page 7, Line 137)

32) 147: “…using Illumina reads as follows: “
R: Thank you. We corrected is as suggested. (Page 7, Line 150)

33) 154: as the previously reported
R: Thank you. We corrected is as suggested. (Page 8, Line 159)

34) 157: 3D de novo assembly [37] softwares to improve the assembly
R: Thank you. We corrected is as suggested. (Page 8, Line 162)

35) 158: change “quite possibly” to “likely”
R: Thank you. We corrected is as suggested. (Page 8, Line 164)

36) 168: Please specify which BUSCO database you are using to assess completeness
R: Thank you for the good suggestion. We used the insecta_odb9 database as the BUSCO database. We have added it in the revised manuscript. (Page 8, Line 174/5)

37) 178: suggest contiguity not continuity
R: Thank you for careful checking. We corrected is as suggested. (Page 9, Line 191)

38) 225: “also searched all predicted gene sequences against GenBank”
R: Thank you. We corrected is as suggested. (Page 11, Line 250)

39) 244: “to reveal the phylogenetic position”
R: Thank you. We have added the word “the” in the revised manuscript. (Page 12, Line 269)

40) 251:“one-to-one single copy orthologs”
R: Thank you. We corrected is as suggested. (Page 12, Line 276)

41) 252: Suggested edits: their nucleic acid sequences were aligned using PRANK (version 3.8.31) [82]. Gene alignments were concatenated and phylogenetic trees were constructed using RAxML (version 7.2.8; RAxML, RRID:SCR 254 006086) [83] with the GTR+G+I model. The phylogeny was further analyzed PAML MCMCtree (version 4.5; PAML, RRID:SCR 014932) program [84], and calibrated with published timings for the divergence of different species [85].
R: Thank you for the good suggestion. We corrected is as suggested. (Page 13, Line 279-291)

42) 258: Xuthus
R: Thank you. Here, “xuthus” is a species name (P. xuthus). In original MS, “P.” and “xuthus” were separated in different line, which produced a confusement.

43) 258: suggest “The Papilio genus is monophyletic”

Powered by Editorial Manager® and ProduXion Manager® from Aries Systems Corporation

R: Thank you. We corrected is as suggested. (Page 13, Line 293/4)

44) 264: suggest “We also inferred the demographic history of P. bianor by mapping Illumina short reads to the assembled genome and applying PSMC analysis.”
R: We do appreciate reviewer’s professional indication. We have revised it accordingly: “We also inferred the demographic histories of P. bianor applying the Pairwise Sequentially Markovian Coalescence (PSMC; PSMC, RRID:SCR_017229; with -p 64*1 parameters) analysis [93] (3.56 × 10-3 mutations per site per generation calculated by r8s [94]; three or four generations per year [48]), which was carried out by mapping Illumina short reads to the assembled genome with BWA (version 0.7.12-r1039; BWA, RRID:SCR_010910) [58] and calling variants with SAMtools (version 1.3.1; SAMTOOLS, RRID:SCR_002105; with samtools mepileup -C50 –uf parameters) [95].” (Page 13-14, Line 299-306)

45) 279: “The P. bianor diverged..”
R: Thank you. We corrected it as suggested. (Page 14, Line 319)

46) 594: “Obtained from Chinese group”- please provide appropriate citation
R: Thank you. We added the citation ([9]). (Line 630)

47) 599: Consider changing “segments” to “functional classes” or equivalent, more informative term
R: Thank you. We changed “segments” to “functional classes” (Line 635)

48) 602: Note plot is based on PSMC in legend
R: Thank you for the suggestive comments. We redescribed the legend of (d) as follows: (d) The dynamic changes of the effective population size are plotted using PSMC software, with 100 bootstrap replicates to test the robust variations. The parameter “g” represents the generation time in years, and the parameter “μ” means the per generation mutation rate. (Line 641-645)

49) 631: Suggest changing to: The Hi-C data were filtered by HiC-Pro software. In total, 6,690,421 pairs of reads, corresponding 68.04% of the total Hi-C dataset, were used in downstream analyses.
R: Thank you. We improved it as suggested. (Line 687/8)

50) Table S4: I suspect “Contiguity” not continuity is the term you mean.
R: Thank you. We corrected it as suggested. (Line 690)

51) Table S5: please state which BUSCO library you are using for comparison
R: Thank you. We improved it as suggested. (Line 693)

References

1.Stamatakis A. RAxML version 8: a tool for phylogenetic analysis and post-analysis of large phylogenies. Bioinformatics. 2014;30 9:1312-3. doi:10.1093/bioinformatics/btu033.
2.Mirarab S, Reaz R, Bayzid MS, Zimmermann T, Swenson MS and Warnow T. ASTRAL: genome-scale coalescent-based species tree estimation. Bioinformatics. 2014;30 17:I541-I8. doi:10.1093/bioinformatics/btu462.
3.Zhang W, Westerman E, Nitzany E, Palmer S and Kronforst MR. Tracing the origin and evolution of supergene mimicry in butterflies. Nature communications. 2017;8 doi:10.1038/S41467-017-01370-1.
4.Li XY, Fan DD, Zhang W, Liu GC, Zhang L, Zhao L, et al. Outbred genome sequencing and CRISPR/Cas9 gene editing in butterflies. Nature communications. 2015;6 doi:10.1038/Ncomms9212.
5.Smith A, Hubley R and Green P. RepeatMasker Open-4.0.(2013-2015). 2016.
6.Lavoie CA, Platt RN, Novick PA, Counterman BA and Ray DA. Transposable element evolution in Heliconius suggests genome diversity within Lepidoptera. Mobile DNA. 2013;4 1:21.
7.Platt RN, Blanco-Berdugo L and Ray DA. Accurate transposable element annotation is vital when analyzing new genome assemblies. Genome biology and evolution. 2016;8 2:403-10.
8.Edgar RC. MUSCLE: multiple sequence alignment with high accuracy and high throughput. Nucleic acids research. 2004;32 5:1792-7. doi:10.1093/nar/gkh340.
9.Capella-Gutierrez S, Silla-Martinez JM and Gabaldon T. trimAl: a tool for automated

	alignment trimming in large-scale phylogenetic analyses. Bioinformatics. 2009;25 15:1972-3. doi:10.1093/bioinformatics/btp348.<br>10.Rice P, Longden I and Bleasby A. EMBOSS: The European molecular biology open software suite. Trends In Genetics. 2000;16 6:276-7. doi:10.1016/S0168-9525(00)02024-2.
Additional Information:	
Question	Response
Are you submitting this manuscript to a special series or article collection?	No
**Experimental design and statistics**<br><br>Full details of the experimental design and statistical methods used should be given in the Methods section, as detailed in our Minimum Standards Reporting Checklist. Information essential to interpreting the data presented should be made available in the figure legends.<br><br>Have you included all the information requested in your manuscript?	Yes
**Resources**<br><br>A description of all resources used, including antibodies, cell lines, animals and software tools, with enough information to allow them to be uniquely identified, should be included in the Methods section. Authors are strongly encouraged to cite Research Resource Identifiers (RRIDs) for antibodies, model organisms and tools, where possible.<br><br>Have you included the information requested as detailed in our Minimum Standards Reporting Checklist?	Yes
**Availability of data and materials**<br><br>All datasets and code on which the conclusions of the paper rely must be either included in your submission or deposited in publicly available repositories (where available and ethically appropriate), referencing such data using	Yes

Powered by Editorial Manager® and ProduXion Manager® from Aries Systems Corporation

a unique identifier in the references and in the “Availability of Data and Materials” section of your manuscript.<br><br>Have you have met the above requirement as detailed in our Minimum Standards Reporting Checklist?	

Powered by Editorial Manager® and ProduXion Manager® from Aries Systems Corporation

Manuscript

Click here to access/download;Manuscript;GIGA-D-19-00120_Pb-Genome_MS_20190807-HL.docx

Click here to view linked References

Chromosomal-level reference genome of Chinese peacock butterfly (*Papilio bianor*) based on third-generation DNA sequencing and Hi-C analysis

Sihan Lu[1,2,†], Jie Yang[1,†], Xuelei Dai[3,†], Feiang Xie[4,†], Jinwu He[1], Zhiwei Dong[2], Junlai Mao[4], Guichun Liu[1,2], Zhou Chang[2], Ruoping Zhao[2], Wenting Wan[1], Ru Zhang[1], Yuan Li[5], Wen Wang[2,6,*,#], Xueyan Li[2,*]

[1] Center for Ecological and Environmental Sciences, Northwestern Polytechnical University, Xi'an, Shaanxi 710072, China.

[2] State Key Laboratory of Genetic Resources and Evolution, Kunming Institute of Zoology, Chinese Academy of Sciences, Kunming, Yunnan 650223, China.

[3] Key Laboratory of Animal Genetics, Breeding and Reproduction of Shaanxi Province, College of Animal Science and Technology, Northwest A&F University, Yangling 712100, China

[4] School of Marine Science and Technology, Zhejiang Ocean University, Zhoushan, Zhejiang 316022, China

[5] Nextomics Biosciences Institute, Wuhan, Hubei 430000, China

[6] Center for Excellence in Animal Evolution and Genetics, Kunming, Yunnan 650223, China

[†]These authors contributed equally to this work.

[*]Correspondence should be addressed to L.X.Y (lixy@mail.kiz.ac.cn), W.W (wwang@mail.kiz.ac.cn).

[#]Current address: Center for Ecological and Environmental Sciences, Northwestern Polytechnical University, Xi'an, Shaanxi 710072, China

ORCIDs:

Sihan Lu, 0000-0003-2354-7407;

Jie Yang, 0000-0002-1731-5266;

Xuelei Dai, 0000-0002-7681-7338;

Feiang Xie, 0000-0003-3947-8645;

Jinwu He, 0000-0003-1681-9769;

Junlai Mao, 0000-0001-5157-0917;

Guichun Liu, 0000-0002-0899-7808;

Ruoping Zhao, 0000-0002-2196-973X;

Ru Zhang, 0000-0003-3216-2787;

Yuan Li, 0000-0002-4802-0305;

Wen Wang, 0000-0002-7801-2066;

Xueyan Li, 0000-0003-0457-7846.

Abstract

Background: *Papilio bianor* Cramer, 1777 (commonly known as the Chinese peacock butterfly) (Insecta, Lepidoptera, Papilionidae) is a widely distributed swallowtail butterfly with a wide number of geographic populations ranging from the Southeast of Russia to China, Japan, India, Vietnam, Myanmar and Thailand. Its wing color consists of both pigmentary colored scales (black, reddish) and structural colored scales (iridescent blue or green dust). A high-quality reference genome of *P. bianor* is an important foundation for investigating iridescent color evolution, phylogeography, and the evolution of swallowtail butterflies.

Findings: We obtained a chromosome-level *de novo* genome assembly of the highly heterozygous *P. bianor* using long Pacific Biosciences (PacBio) sequencing reads and high-throughput chromosome conformation capture technology. The final assembly is 421.52 Mb on 30 chromosomes (29 autosomes and 1 Z sex chromosomes) with 13.12 Mb scaffold N50. In total, 15,375 protein-coding genes and 233.09 Mb of repetitive sequences were identified. Phylogenetic analyses indicated that *P. bianor* was separated from a common ancestor of swallowtails about 23.69-36.04 million years ago. Demographic history suggested that the population expansion of this species from the last interglacial period to the last glacial maximum possibly resulted from its decreased natural enemies and its adaptation to climate change during glacial period.

Conclusions: We present a high-quality chromosome-level reference genome of *P. bianor* using long-read single-molecule sequencing and Hi-C-based chromatin interaction maps. Our results lay the foundation for exploring the genetic basis of special biological features of *P. bianor*, and also provide a useful datasource for comparative genomics and phylogenomics among butterflies and moths.

Keywords: *Papilio bianor*; single-molecule real-time (SMRT) sequencing; High-through chromosome conformation capture map; chromosome-level reference genome; Butterfly.

Background information

Butterflies are widely considered as one of most aesthetically appealing and popular animals due to their extraordinarily diverse wing patterns among species, populations, sexes, and seasonal forms [1-3]. They also have many other intriguing traits such as complex life cycles, diverse larval morphology and habits, and high species diversity [4]. In light of this interest, butterflies have been regarded as important model organisms in different fields from morphology, physiology, ecology, development, genetics to evolutionary biology [4-6] since Darwin proposed his theory of natural selection in 1859 [7]. Back in 1864, Bates, the famous pioneer of mimicry theory, predicted that *"the study of butterflies…will someday be valued as one of the most important branches of Biological science."* [8]. With recent technological advances, it is possible to conduct direct analysis (and even manipulation) of the genomes of individuals sampled from natural habitats without the need of inbreeding to reduce heterozygosity or to develop lab lines [9-11]. Meaning butterflies have been becoming a promising system to explore the genetics and evolution of morphological diversification and speciation.

Compared with butterfly diversity of more than 18,000 described species [12], only 37 butterfly species in 6 families including five swallowtails (Papilionidae) have their reference genomes dissected (until May 1, 2019) [9, 13-31]. Among them, chromosomal-level reference genomes have been assembled only for two nymphalids (*Heliconius melpomene* and *Melitaea cinxia*) and one swallowtail (*Papilio xuthus*) [9, 24, 25] using linkage map methods. Chromosomal-level reference genomes for more butterflies are not only indispensable to identify subtle genetic variations underpinning morphological traits which may often result from small mutations in

regulatory elements [32, 33], but also will provide a unique opportunity to promote the evolutionary biological studies on butterflies as an important model system.

The development of third generation single molecule technology has paved the way for the dissection of complex genomes of different kinds of wild organisms including butterflies [25, 28, 30, 34, 35]. Combined with high-throughput chromosome conformation capture technology, which was developed to identify chromatin interactions across the entire genome and is now also used as a powerful tool to assist genome assembly [36], chromosomal-level reference genomes have been obtained for many organisms including such insects as fruit fly [37], mosquitoes [38], and moths [39, 40]. Despite this, up to now there have been no such examples combining single molecule sequencing and Hi-C technologies to assemble chromosomal-level reference genomes reported for butterflies.

Papilio bianor Cramer, 1777 (NCBI: txid76199) (Papilionidae, Papilioninae, Papilionini) (**Fig. 1a**), also known as the Chinese peacock black swallowtail emerald or the Chinese peacock, is a widely distributed swallowtail butterfly with a large range of geographic populations ranging from the Southeast of Russia to China, Japan, India, Vietnam, Myanmar and Thailand [41-43]. Its larvae mainly feed on plants of the family Rutaceae, such as *Citrus reticulate*, *Euodia meliifolia* and *Zanthoxylum bungeanum* [41, 44, 45], and its complete life cycle lasts 40-50 days. Its wing colors consist of both pigmentary colored scales (black, reddish) and structurally-colored scales (iridescent blue or green dust) [45], which makes it a promising model to explore the origin and evolution of combined colors in insects. Scientific interests in *P. bianor* have long existed, for examples in its

prothoracicotropic hormones (PTTHs) [46], oviposition behavior [44, 47, 48], phylogenetic position and species delimitation [49-53], chromosome numbers [54] or mitochondrial genome [50, 55]. Here, combining PacBio SMRT and Hi-C technologies, we constructed the chromosome-level reference genome of *P. bianor* (30 chromosomes).

Data Description

Insect collection and breeding

Wild eggs of *P. bianor* were collected in a Northern surburb of Kunming City (Yunnan, China), and then reared under the conditions of 26 ℃, 80% relative humidity with 16h/8h light/darkness. The hatched larvae were fed with Rutaceous plant *Zanthxylum piperitum* under the same conditions. Two 5th instar larvae were collected for Hi-C sequencing. Pupa were reared under the same conditions as the eggs until their eclosion. Adults were collected for a genome survey using the IIlumina sequencing platform and for *de novo* genome sequencing using PacBio platform.

Genome survey using Illumina sequencing technology

Genomic DNA was isolated from the thorax and abdomen of single male adult using a Gentra Puregene Blood kit (Qiagen, Germany) following manual instructions. Paired-end libraries of two different insertion sizes (150 bp and 500 bp) were constructed and sequenced on an Illumina HiSeq2000 platform at BGI (Shenzhen, China). The total number of sequencing reads was approximately 16.45 Gb for PE150 and 28.42 Gb for PE500 (**Table S1**). We estimated genome size using Illumina short reads (PE150 and PE500), by *k-mer* distribution analysis with k = 17, using the formula: G = k-

mer_number/k-mer_depth [56]. Our data indicate that *P. bianor* has an estimated genome size of 496.05 Mb and a high heterozygosity of 1.81% (**Fig. S1** & **Table S2**).

Library construction and sequencing using SMRT and Hi-C technologies

Genomic DNA was extracted from the thorax and abdomen of another male adult and used to construct one 20-kb library for the PacBio platform according to the manufacturers' protocols (NextOmics, China). With ten single-molecular real-time (SMRT) cells in the PacBio RSII platform, we generated 43.19 Gb subreads with a average read length of 16.4 kb after removing adaptor sequences within sequences (**Table S1**). The long subreads were used fo*r de novo* genome assembly of *P. bianor*.

A sample mixed from the whole bodies of two male larval individuals (fifth instar) was used for library construction for Hi-C sequencing according to the methods reporting in the previous study [36]. A 400-700 bp library was sequenced on the Illumina HiSeq X Ten platform with 150 paired-end mode, resulted in ~75.11 Gb raw reads (**Table S1**).

Chromosomal-level genome assembly

Considering the high heterozygosity of *P. bianor* (1.81%: **Fig. S1** & **Table S2**), we firstly performed a PacBio-only assembly using Wtdbg (version 1.2.8; Wtdbg, RRID:SCR_017225; with --tidy-reads 5000 -k 0 -p 17 -S 1) [57], which is a *de novo* sequence assembler for noisy long reads produced by PacBio or Oxford Nanopore Technologies and is based on the fuzzy Bruijn graph (FBG) algorithm. Secondly, to eliminate the high error rate of the PacBio long reads, we further polished the PacBio-only assembled sequences using Illumina reads as follows: all the Illumina reads were

mapped to the PacBio-only assembly with BWA (version 0.7.12-r1039; BWA, RRID:SCR_010910) [58], which was further corrected with 2-round Pilon (version 1.21; Pilon, RRID:SCR_014731) correction [59, 60]. Thirdly, because the polished assembly still contained a number of shorter contigs with significantly lower coverage, which perhaps represents the high heterozygous regions that were not merged to equivalent segments in the homologous chromosomes, we used a looser cutoff for identity (> 90%) to merge the contigs with lower coverage and smaller size (size < 1000 bp and coverage < 50 or size < 10000 bp and coverage < 35) into the longer contigs as previously reported [14]. Fourthly, the raw reads generated from the Hi-C sequencing were mapped to the polished assembled genome using Juicer (version 1.5; Juicer, RRID:SCR_017226) [61] and 3D *de novo* assembly (version 180114; 3D *de novo* assembly, RRID:SCR_017227) [38] to improve the assembly. Approximately 90.50% of contigs were anchored onto 30 super-scaffolds (**Fig. 1b** & **Table S3,** more details see **Fig. S2**), which likely correspond to the 30 chromosomes as reported by cytogenetic karyotype [54]. Finally, we obtained the chromosomal-level high-quality assembly of *P. bianor* with a total length of ~421.52 Mb and the longest scaffold N50 (13.12 Mb) of any published butterfly genome to date (**Table 1** & **Table S4**). The assembled genome accounts for 85% of the estimated genome size (496.05 Mb) by the *k-mer* distribution analysis (**Table S2**).

Quality evaluation of assembled genome

The assembled genome quality was evaluated using three methods. Firstly, the completeness of the assembly was evaluated by Benchmarking Universal Single-Copy Orthologs (BUSCO) (version 2.0; BUSCO, RRID:SCR_015008) [62] with the insecta_odb9 BUSCO set. The BUSCO data showed that *P. bianor* assembly covered

96.90% of the core genes with 96.30% covered genes complete (**Table S5**), which are similar to those of other published high quality butterfly genomes (**Table 1**). We also checked the mapping rates of Illumina and PacBio reads to the *P. bianor* assembly by BWA (version 0.7.12-r1039; BWA, RRID:SCR_010910) [58] and BLASR (BLASR; RRID:SCR_000764) [63]. Our result indicate 96.31% Illumina reads mapped to the assembled genome with few heterozygous regions (**Fig. S3 & Table S6);** 96.86% PacBio reads also mapped to the assembled genome with few heterozygous regions (**Fig. S4 & Table S7**). Thirdly, we compared the syntenic relationships of *P. bianor* genome with that of *P. xuthus*, which is the only chromosomal-level assembly (by linkage map methods) [9] among all *Papilio* reference genomes released to date, and thus was considered to the best-assembled one (**Fig. 1c**). We found that 61,082,412 bp of the *P. bianor* assembled genome could be aligned (1:1) with high-confidence (-m 0.01) to the *P. xuthus* reference genome. All these results suggest that the *P. bianor* genome, which is assembled based on PacBio reads, Illumina reads and Hi-C data sequenced from different wild individuals, is of high quality (including completeness, base level contiguity and accuracy) (**Table 1**).

Genome annotation

Repetitive sequences including tandem repeats and transposable elements (TEs) were searched for in the *P. bianor* assembled genome. To do this, we firstly used Tandem Repeat Finder (version 4.07b; Tandem Repeats Database, RRID:SCR_005659; with 2 7 7 80 10 50 2000 -d -h parameters) [64] to annotate the tandem repeats. Then, TEs were identified using a combination of *de novo* and homology-based approaches at both the DNA and protein levels. At the DNA level, we used RepeatModeler (version 1.0.4; RepeatModeler, RRID:SCR_015027) [65] to construct a *de novo* repeat library,

which built a repeat consensus database with classification information, and then we adopted RepeatMasker (version 4.0.5; RepeatMasker, RRID:SCR_012954) [66] to search similar TEs against the known Repbase TE library (version 16.02) [67] and *de novo* repeat library. We also used LTR_FINDER (LTR Finder, RRID:SCR_015247) [68] to find long terminal repeats. At the protein level, software RepeatProteinMask (version 3.3.0, a package in RepeatMasker) [66] was used to search the assembled *P. bianor* genome against the TE protein database using the WU-BLASTX engine. Finally, we identified and masked 55.30% of the *P. bianor* assembly as repeat regions (**Table S8**), which is the highest in published butterfly genomes (**Table 1**). Among all TEs, the most abundant class of repetitive elements are long interspersed nuclear elements (LINEs, 14.22%), and the next are DNA transposons (8.81%) (**Table S9**). Compared with the reference genomes of other swallowtail butterflies, LINEs, DNA transposons and long terminal repeats (LTRs) of repeats have expanded in the *P. bianor* genome (**Fig. 2a**). To confirm the reliability of the high repetitive sequences seen in *P. bianor*, which is much higher than those (<35%) of other butterflies (**Table 1**), we also used other *de novo* annotation methods reported by Platt II et al. and Lavoie et al [69, 70] to annotate the repetitive sequences of *P. bianor* genome. Based on these methods, *P. bianor* genome possesses 53% repeat elements, similar to the previous annotated results (**Table 1 & Table S8**), thus confirming high repetitive sequences in *P. bianor* genome.

To annotate protein-coding genes of *P. bianor*, we used both *de novo* and homology-based gene prediction approaches. For *de novo* gene prediction, the repeat-masked genome was analyzed by SNAP (version 2006-07-28; SNAP, RRID:SCR_002127) [71], GENSCAN (version 1.0; GENSCAN, RRID:SCR_012902) [72], glimmerHMM

(version 3.0.3; glimmerHMM, RRID:SCR_002654) [73], and AUGUSTUS (version 2.5.5; Augustus, RRID:SCR_008417) [74]. For homology-based predictions, the protein sequences from eight insects including beetle *Tribolium castaneum* [75], fruit fly *Drosophila melanogaster* [76], silkworm *Bombyx mori* [77], moth *Helicoverpa armigera* [78], and four butterflies *Papilio polytes* [23], *Papilio xuthus* [9], *Heliconius melpomene* [24] and *Danaus plexippus* [20], were used as templates for homology-based gene prediction. Then we used TBLASTN (version 2.2.26; TBLASTN, RRID:SCR_011822) [79] with an E-value cut-off of 1e-5 to align the protein sequences of the reference gene set to *P. bianor* genome, and GeneWise (v2.2.0; GeneWise, RRID:SCR_015054) [80] to perform more precise alignment. Gene sequences with length < 150 bp or percent identity < 25% were removed. EvidenceModeler software (EVM, version 1.1.1; EvidenceModeler, RRID:SCR_014659) [81] was used to integrate the genes predicted by the homology and *de novo* approaches and generate a comprehensive, non-redundant gene set. Finally, 15,375 protein-coding genes were annotated in the assembled *P. bianor* genome (**Table S10**), which is similar to those published reference genomes of other swallowtail butterflies (**Fig. S3**).

The KEGG, TrEMBL, SwissProt and Cog databases were searched for best matches to *P. bianor* the protein sequences yielded by EVM software (version 1.1.1; EvidenceModeler, RRID:SCR_014659), using BLASTP (version 2.2.26; BLASTP, RRID:SCR_001010) with an (E)-value cutoff of 1e-5, and Pfam, PRINTS, ProDom and SMART databases were searched for known motifs and domains in our sequences using InterProScan software (version 5.18-57.0; InterProScan, RRID:SCR_005829) [82]. We also searched all predicted gene sequences against GenBank nonredundant

protein (nr) using BLASTN (BLASTN, RRID:SCR_001598) with a maximal e-value of 1e-5. In sum, 13,343 genes were annotated with at least 1 related function, which accounts for about 86.78% of the *P. bianor* annotated genes (**Table S11**).

Gene family identification and phylogenetic analysis

We use OrthoMCL (version 2.0.9; OrthoMCL DB: Ortholog Groups of Protein Sequences, RRID:SCR_007839) [83] to cluster the *P. bianor* annotated genes with an (E)-value cutoff of 1 e-5, and Markov Chain Clustering with default inflation parameter in an all-to-all BLASTP analysis of entries for the reference genomes of six swallowtail butterflies including *P. bianor* in this study and other five published so far (*P. polytes*, *P. xuthus*, *P. machaon*, *P. glaucus*, and *P. memnon*). The result showed that 293 gene families were specific to *P. bianor* (**Fig. 2b**). Using Computational Analysis of gene Family Evolution (CAFE; version 4.0.1) [84], we also identified 375 expanded gene families and 1863 contracted gene families in *P. bianor*. The *P. bianor* expanded gene families were enriched in 17 GO categories and the contracted gene families were enriched in 14 GO categories, most of which are related to oxygen metabolism (**Table S12** & **Table S13**).

To reveal the phylogenetic position of *P. bianor* among Papilionoidea, we selected 14 butterfly species in five families (Papilionidae (6): *Papilio xuthus*, *Papilio polytes, Papilio machaon, Papilio glaucus, Papilio memnon*; Hesperiidae (1): *Lerema accius*; Pieridae (2): *Phoebis sennae, Pieris rapae*; Nymphalidae (2): *Bicyclus anynana, Heliconius melpomene*; Riodinidae (2): *Calephelis nemesis, Calephelis virginiensis*; Lycaenidae (1): *Calycopis cecrops*) [9, 13-15, 17, 21, 23, 24, 26-28] with 2 moths (*Bombyx mori* [77], *Helicoverpa armigera* [78]) as outgroups for phylogenetic

analysis. 1378 one-to-one single copy orthologs which contain only one protein for each species were collected and clustered by OrthoMCL (version 2.0.9; OrthoMCL DB: Ortholog Groups of Protein Sequences, RRID:SCR_007839) [83] from these 16 species and their nucleic acid sequences were aligned using PRANK (version 3.8.31; PRANK, RRID:SCR_017228) [85]. Gene alignments were concatenated and phylogenetic trees were constructed using RAxML (version 7.2.8; RAxML, RRID:SCR_006086) [86] with the GTR+G+I model. Furthermore, in order to make our result more clarified, we also have constructed the gene trees for each of the orthologs with RAxML software (version 7.2.8; RAxML, RRID:SCR_006086) [84] by choosing the GTR+G+I model and inferred the species tree from these with ASTRAL software (version 5.6.3) [87] (**Fig. S4**). As expected, the results are consistent with each other. To further investigate the divergence time of these species, the phylogeny was further analyzed by MCMCtree in PAML (version 4.5; PAML, RRID:SCR_014932) software [88] using default parameters, and calibrated with published divergent times of some nodes estimated from fossil evidence or obtained from the TIMETREE website [89]. Our phylogenetic tree showed that *P. bianor* clusters at the base of *P. machaon* and *P. xuhtus*, and diverged from them 23 million years ago (mya); the *Papilio* genus was monophyletic with a crown node age of approximately 41.07-56.86 mya (**Fig. 2c**). This tree is largely consistent with those constructed from cytochrome oxidases I (COI), cytochrome oxidases II (COII) and elongation factor 1α (EF-1α) [90, 91], and from 425 loci from two outgroups and 173 species of butterflies[92].

We also inferred the demographic histories of *P. bianor* applying the Pairwise Sequentially Markovian Coalescence (PSMC; PSMC, RRID:SCR_017229; with -p

64*1 parameters) analysis [93] (3.56×10^{-3} mutations per site per generation calculated by r8s [94]; three or four generations per year [48]), which was carried out by mapping Illumina short reads to the assembled genome with BWA (version 0.7.12-r1039; BWA, RRID:SCR_010910) [58] and calling variants with SAMtools (version 1.3.1; SAMTOOLS, RRID:SCR_002105; with samtools mepileup -C50 –uf parameters) [95]. Our result suggested that the effective population size increased significantly corresponded to the transition phase from the last interglacial period (LIG, approximately 0.14-0.12 million years before present) to the last glacial maximum (LGM, approximately 0.021-0.018 million years before present) (**Fig. 2d**), which is in good agreement with the other five published *Papilio* species [96]. We hypothesize that the population expansion of this species possibly results from the decrease of its natural enemies (e.g. birds or lizards) and from its adaptation to climate change during LIG and LGM.

Conclusion

We present the chromosomal-level genome assembly of *P. bianor* with a contig and scaffold N50 of 5.50 Mb and 12.51 Mb, respectively. The assembled genome included 15,375 protein-coding genes, 293 species-specific gene families, 375 expanded gene families and 1863 contracted gene families. *P. bianor* diverged from other *Papilio* approximately 23.69-36.04 mya. Our results also show that the effective population size of *P. bianor* increased significantly during the glacial period. Our results lay the foundation for exploring the special biological features of the Chinese peacock butterfly, and also provide a useful data source for comparative genomics and phylogenomics among butterflies and Lepidopterans.

Availability of supporting data

The raw reads have been deposited at NCBI in the sequence read archive (SRA) under BioProject Number: PRJNA530186. The chromosome-level genome, annotation, and other supporting data are also available via the *GigaScience* database, *Giga*DB [97].

Abbreviations

bp: base pair; kb: kilo base; Mb: mega base; Gb: giga base; PE: paired-end; BUSCO: Benchmarking Universal Single-Copy Orthologs; FBG: fuzzy Bruijn graph; TE: transposable element; GO: gene ontology; KEGG: Kyoto Encyclopedia of Genes and Genomes.

Competing interests

The authors declare that there have no competing interests.

Author contributions

X.L., W.W conceived and supervised the study. J.H., Z.D., Z.C., G.L. fed and collected the samples. G.L., J.H. extracted the genomic DNA. Y.L took charge of Hi-C sequencing. S.L., X.D. assembled the genome. S.L., J.Y., F.X. carried out the quality assessment, repeat annotation, and gene annotation. J.Y., F.X., J.M. carried out evolutionary analyses. S.L. uploaded the raw read data, genome assembly, and annotation in the GenBank and *GigaScience* (GigaDB) databases. S.L., X.L., W.W. wrote the manuscript. All authors read and approved the final manuscript.

Acknowledgements

This work was supported by grants from the National Natural Science Foundation of China (No. 31621062) (to WW), the Chinese Academy of Sciences (XDB13000000 (to WW), and CAS “Light of West China” (to LXY).

References

1. Boggs CL, Watt WB and Ehrlich PR. Butterflies: ecology and evolution taking flight. University of Chicago Press; 2003.
2. Joron M and Mallet JLB. Diversity in mimicry: paradox or paradigm? Trends in ecology & evolution. 1998;13 11:461-6. doi:Doi 10.1016/S0169-5347(98)01483-9.
3. Nijhout HF. The development and evolution of butterfly wing patterns. Smithson Inst. 1991;293.
4. Heikkila M, Kaila L, Mutanen M, Pena C and Wahlberg N. Cretaceous origin and repeated tertiary diversification of the redefined butterflies. Proceedings Biological sciences. 2012;279 1731:1093-9. doi:10.1098/rspb.2011.1430.
5. Kawahara AY and Breinholt JW. Phylogenomics provides strong evidence for relationships of butterflies and moths. Proceedings Biological sciences. 2014;281 1788:20140970. doi:10.1098/rspb.2014.0970.
6. Mitter C, Davis DR and Cummings MP. Phylogeny and Evolution of Lepidoptera. Annual review of entomology. 2017;62:265-83. doi:10.1146/annurev-ento-031616-035125.
7. Darwin C. The Origin of Species; And, the Descent of Man. Modern library; 1859.
8. Bates H. New species of butterflies from Guatemala and Panama, collected by Osbert Salvin and F. du Cane Godman, Esqs. Entomologist's monthly Magazine. 1864;1 1/7:1-164.
9. Li X, Fan D, Zhang W, Liu G, Zhang L, Zhao L, et al. Outbred genome sequencing and CRISPR/Cas9 gene editing in butterflies. Nature Communications. 2015;6:8212. doi:10.1038/ncomms9212.
10. Zhang LL and Reed RD. Genome editing in butterflies reveals that spalt promotes and Distal-less represses eyespot colour patterns. Nature Communications. 2016;7 doi:10.1038/Ncomms11769.
11. Markert MJ, Zhang Y, Enuameh MS, Reppert SM, Wolfe SA and Merlin C. Genomic Access to Monarch Migration Using TALEN and CRISPR/Cas9-Mediated Targeted Mutagenesis. G3-Genes Genom Genet. 2016;6 4:905-15. doi:10.1534/g3.116.027029.
12. van Nieukerken EJ, Kaila L, Kitching IJ, Kristensen NP, Lees D, Minet J, et al. Order Lepidoptera Linnaeus, 1758. Zootaxa. 2011;3148:212-21.
13. Cong Q, Borek D, Otwinowski Z and Grishin NV. Skipper genome sheds light on unique phenotypic traits and phylogeny. BMC Genomics. 2015;16:639. doi:10.1186/s12864-015-1846-0.
14. Cong Q, Borek D, Otwinowski Z and Grishin NV. Tiger Swallowtail Genome Reveals Mechanisms for Speciation and Caterpillar Chemical Defense. Cell reports. 2015;10 6:910-9. doi:10.1016/j.celrep.2015.01.026.
15. Shen J, Cong Q, Kinch LN, Borek D, Otwinowski Z and Grishin NV. Complete genome of *Pieris rapae*, a resilient alien, a cabbage pest, and a source of anti-cancer proteins. F1000Res. 2016;5:2631. doi:10.12688/f1000research.9765.1.
16. Cong Q, Li W, Borek D, Otwinowski Z and Grishin NV. The Bear Giant-Skipper genome suggests genetic adaptations to living inside yucca roots. Molecular genetics and genomics : MGG. 2018; doi:10.1007/s00438-018-1494-6.
17. Iijima T, Kajitani R, Komata S, Lin CP, Sota T, Itoh T, et al. Parallel evolution of Batesian mimicry supergene in two *Papilio* butterflies, *P. polytes* and *P. memnon*. Science Advances. 2018;4 4 doi:10.1126/sciadv.aao5416.
18. Zhan S, Merlin C, Boore JL and Reppert SM. The monarch butterfly genome yields insights into long-distance migration. Cell. 2011;147 5:1171-85. doi:10.1016/j.cell.2011.09.052.
19. Hill JA, Neethiraj R, Rastas P, Clark N, Morehouse N, de la Paz Celorio-Mancera M, et al. Unprecedented reorganization of holocentric chromosomes provides insights into the enigma of lepidopteran chromosome evolution. Sci Adv. 2019 Jun 12;5(6):eaau3648. doi: 10.1126/sciadv.aau3648.

20. Zhan S, Zhang W, Niitepold K, Hsu J, Haeger JF, Zalucki MP, et al. The genetics of monarch butterfly migration and warning colouration. Nature. 2014;514 7522:317-21. doi:10.1038/nature13812.
21. Cong Q, Shen JH, Warren AD, Borek D, Otwinowski Z and Grishin NV. Speciation in Cloudless Sulphurs Gleaned from Complete Genomes. Genome Biology and Evolution. 2016;8 3:915-31. doi:10.1093/gbe/evw045.
22. Talla V, Suh A, Kalsoom F, Dinca V, Vila R, Friberg M, et al. Rapid Increase in Genome Size as a Consequence of Transposable Element Hyperactivity in Wood-White (Leptidea) Butterflies. Genome Biology and Evolution. 2017;9 10:2491-505. doi:10.1093/gbe/evx163.
23. Nishikawa H, Iijima T, Kajitani R, Yamaguchi J, Ando T, Suzuki Y, et al. A genetic mechanism for female-limited Batesian mimicry in *Papilio* butterfly. Nature Genetics. 2015;47 4:405-U169. doi:10.1038/ng.3241.
24. Dasmahapatra KK, Walters JR, Briscoe AD, Davey JW, Whibley A, Nadeau NJ, et al. Butterfly genome reveals promiscuous exchange of mimicry adaptations among species. Nature. 2012;487 7405:94-8. doi:10.1038/nature11041.
25. Ahola V, Lehtonen R, Somervuo P, Salmela L, Koskinen P, Rastas P, et al. The Glanville fritillary genome retains an ancient karyotype and reveals selective chromosomal fusions in Lepidoptera. Nature Communications. 2014;5 doi:10.1038/Ncomms5737.
26. Cong Q, Shen JH, Borek D, Robbins RK, Otwinowski Z and Grishin NV. Complete genomes of Hairstreak butterflies, their speciation, and nucleo-mitochondrial incongruence. Scientific Reports. 2016;6 doi:10.1038/Srep24863.
27. Cong Q, Shen JH, Li WL, Borek D, Otwinowski Z and Grishin NV. The first complete genomes of Metalmarks and the classification of butterfly families. Genomics. 2017;109 5-6:485-93. doi:10.1016/j.ygeno.2017.07.006.
28. Nowell RW, Elsworth B, Oostra V, Zwaan BJ, Wheat CW, Saastamoinen M, et al. A high-coverage draft genome of the mycalesine butterfly *Bicyclus anynana*. GigaScience. 2017;6 7 doi:10.1093/gigascience/gix035.
29. Mallet J. New genomes clarify mimicry evolution. Nature genetics. 2015;47 4:306-7. doi:10.1038/ng.3260.
30. Davey JW, Chouteau M, Barker SL, Maroja L, Baxter SW, Simpson F, et al. Major Improvements to the *Heliconius melpomene* Genome Assembly Used to Confirm 10 Chromosome Fusion Events in 6 Million Years of Butterfly Evolution. G3-Genes Genom Genet. 2016;6 3:695-708. doi:10.1534/g3.115.023655.
31. Shen J, Cong Q, Borek D, Otwinowski Z and V Grishin N. Complete genome of *Achalarus lyciades*, the first representative of the Eudaminae subfamily of skippers. Current Genomics. 2017;18 4:366-74.
32. Loehlin DW and Carroll SB. EVOLUTIONARY BIOLOGY Sex, lies and butterflies. Nature. 2014;507 7491:172-3. doi:Doi 10.1038/Nature13066.
33. Brunetti CR, Selegue JE, Monteiro A, French V, Brakefield PM and Carroll SB. The generation and diversification of butterfly eyespot color patterns. Current Biology. 2001;11 20:1578-85. doi:Doi 10.1016/S0960-9822(01)00502-4.
34. VanBuren R, Bryant D, Edger PP, Tang HB, Burgess D, Challabathula D, et al. Single-molecule sequencing of the desiccation-tolerant grass *Oropetium thomaeum*. Nature. 2015;527 7579:508-U209. doi:10.1038/nature15714.
35. Andere AA, Ii RNP, Ray DA and Picard CJ. Genome sequence of *Phormia regina* Meigen (Diptera: Calliphoridae): implications for medical, veterinary and forensic research. BMC Genomics. 2016;17 doi:10.1186/s12864-016-3187-z.
36. Belaghzal H, Dekker J and Gibcus JH. Hi-C 2.0: An optimized Hi-C procedure for high-resolution genome-wide mapping of chromosome conformation. Methods. 2017;123:56-65. doi:10.1016/j.ymeth.2017.04.004.
37. Chakraborty M, VanKuren NW, Zhao R, Zhang XW, Kalsow S and Emerson JJ. Hidden genetic variation shapes the structure of functional elements in *Drosophila*. Nature Genetics. 2018;50 1:20-+. doi:10.1038/s41588-017-0010-y.

38. Dudchenko O, Batra SS, Omer AD, Nyquist SK, Hoeger M, Durand NC, et al. De novo assembly of the *Aedes aegypti* genome using Hi-C yields chromosome-length scaffolds. Science. 2017;356 6333:92-5. doi:10.1126/science.aal3327.
39. Chen WB, Yang XW, Tetreau G, Song XZ, Coutu C, Hegedus D, et al. A high-quality chromosome-level genome assembly of a generalist herbivore, *Trichoplusia ni*. Molecular ecology resources. 2019;19 2:485-96. doi:10.1111/1755-0998.12966.
40. Xiang H, Liu XJ, Li MW, Zhu YN, Wang LZ, Cui Y, et al. The evolutionary road from wild moth to domestic silkworm. Nature ecology & evolution. 2018;2 8:1268-79. doi:10.1038/s41559-018-0593-4.
41. Wu C. Fauna Sinica Insect Vol. 25 Lepidoptera Papilionidae. Beijing: Science Press, 2001.
42. Sinev SY. Catalogue of the Lepidoptera of Russia. Ed. SY Sinev. KMK, Saint-Petersburg-Moscow, 2008.
43. Chou I. Monograph of Chinese butterflies. Zhengzhou: Henan Scientific and Technological Publishing House. 1994:1-854.
44. Ono H, Nishida R and Kuwahara Y. Oviposition stimulant for a Rutaceae-feeding swallowtail butterfly, *Papilio bianor* (Lepidoptera: Papilionidae): Hydroxycinnamic acid derivative from *Orixa japonica*. Applied Entomology and Zoology. 2000;35 1:119-23.
45. Perveen F, Khan A and Sikander. Characteristics of butterfly (Lepidoptera) fauna from Kabal, Swat, Pakistan. Journal of Entomology and Zoology Studies. 2014;2 1:56-69.
46. Yokoyama I, Endo K, Yamanaka A and Kumagai K. Species-specificity in the action of big and small prothoracicotropic hormones (PTTHs) of the swallowtail butterflies, *Papilio xuthus, P-machaon, P-bianor* and *P-helenus*. Zoological Science. 1996;13 3:449-54. doi:Doi 10.2108/Zsj.13.449.
47. Ono H, Nishida R and Kuwahara Y. A dihydroxy-gamma-lactone as an oviposition stimulant for the swallotail butterfly, *Papilio bianor*, from the Rutaceous plant, *Orixa japonica*. Biosci Biotech Bioch. 2000;64 9:1970-3. doi:10.1271/Bbb.64.1970.
48. Dongsheng L. A Preliminary Observation on the Artificial Rearing of Xinyang *Papilio bianor*. JOURNAL OF XINYANG TEACHERS COLLEGE (NATURAL SCIENCE EDITION). 1997;2.
49. Lixin Z, Xiaobing W, Chunsheng W and Banghe Y. Phylogenetic evaluation of *Papilio bianor* and *P. polyctor* (Lepidoptera: Papilionidae). Oriental Insects. 2009;43 1:25-32.
50. Hou LX, Ying S, Yang XW, Yu Z, Li HM and Qin XM. The complete mitochondrial genome of *Papilio bianor* (Lepidoptera: Papilionidae), and its phylogenetic position within Papilionidae. Mitochondrial DNA Part A. 2016;27 1:102-3. doi:10.3109/19401736.2013.873923.
51. Ae S. Some problems in hybrids between *Papilio bianor* and *P. maackii*. Academia (Nanzan Univ). 1962;33:21-8.
52. CHANG Y-J. A study on hybridization of two subspecies of *Papilio bianor* (Lepidoptera, Papilionidae) in Taiwan. Lepidoptera Science. 1990;41 1:1-6.
53. Yamada A. A study of interspecific hybrids between *Papilio bianor* and *P. maackii*. The nature and insects. 1977;12:27-8.
54. Maeki K and Makino S. Chromosome numbers of some Japanese Rhopalocera. Lepid news. 1953;7:36-8.
55. Dong Y, Zhu L-X, Wu Y-f and Wu X-B. The complete mitochondrial genome of the Chinese peacock, *Papilio bianor* (Insecta: Lepidoptera: Papilionidae). Mitochondrial DNA. 2013;24 6:636-8.
56. Li R, Fan W, Tian G, Zhu H, He L, Cai J, et al. The sequence and de novo assembly of the giant panda genome. Nature. 2010;463 7279:311-7. doi:10.1038/nature08696.
57. Ruan J and Li H. Fast and accurate long-read assembly with wtdbg2. BioRxiv. 2019:530972.

58. Li H. Aligning sequence reads, clone sequences and assembly contigs with BWA-MEM. arXiv preprint arXiv:13033997. 2013.
59. Walker BJ, Abeel T, Shea T, Priest M, Abouelliel A, Sakthikumar S, et al. Pilon: An Integrated Tool for Comprehensive Microbial Variant Detection and Genome Assembly Improvement. PloS One. 2014;9 11 doi:10.1371/journal.pone.0112963.
60. Vaser R, Sovic I, Nagarajan N and Sikic M. Fast and accurate de novo genome assembly from long uncorrected reads. Genome Research. 2017;27 5:737-46. doi:10.1101/gr.214270.116.
61. Durand NC, Shamim MS, Machol I, Rao SSP, Huntley MH, Lander ES, et al. Juicer Provides a One-Click System for Analyzing Loop-Resolution Hi-C Experiments. Cell Syst. 2016;3 1:95-8. doi:10.1016/j.cels.2016.07.002.
62. Simao FA, Waterhouse RM, Ioannidis P, Kriventseva EV and Zdobnov EM. BUSCO: assessing genome assembly and annotation completeness with single-copy orthologs. Bioinformatics. 2015;31 19:3210-2. doi:10.1093/bioinformatics/btv351.
63. Chaisson MJ and Tesler G. Mapping single molecule sequencing reads using basic local alignment with successive refinement (BLASR): application and theory. BMC bioinformatics. 2012;13 doi:10.1186/1471-2105-13-238.
64. Benson G. Tandem repeats finder: a program to analyze DNA sequences. Nucleic acids research. 1999;27 2:573-80. doi:Doi 10.1093/Nar/27.2.573.
65. Smith A, Hubley R and Green P. RepeatMasker Open-4.0.(2013-2015). 2016.
66. Chen N. Using RepeatMasker to identify repetitive elements in genomic sequences. Current protocols in bioinformatics. 2004;5 1:4.10. 1-4.. 4.
67. Bao WD, Kojima KK and Kohany O. Repbase Update, a database of repetitive elements in eukaryotic genomes. Mobile DNA-Uk. 2015;6 doi:10.1186/s13100-015-0041-9.
68. Xu Z and Wang H. LTR_FINDER: an efficient tool for the prediction of full-length LTR retrotransposons. Nucleic acids research. 2007;35:W265-W8. doi:10.1093/nar/gkm286.
69. Lavoie CA, Platt RN, Novick PA, Counterman BA and Ray DA. Transposable element evolution in *Heliconius* suggests genome diversity within Lepidoptera. Mobile DNA-Uk. 2013;4 1:21.
70. Platt RN, Blanco-Berdugo L and Ray DA. Accurate transposable element annotation is vital when analyzing new genome assemblies. Genome Biology and Evolution. 2016;8 2:403-10.
71. Korf I. Gene finding in novel genomes. BMC Bioinformatics. 2004;5 doi:Doi 10.1186/1471-2105-5-59.
72. Burge C and Karlin S. Prediction of complete gene structures in human genomic DNA. J Mol Biol. 1997;268 1:78-94. doi:10.1006/jmbi.1997.0951.
73. Majoros WH, Pertea M and Salzberg SL. TigrScan and GlimmerHMM: two open source ab initio eukaryotic gene-finders. Bioinformatics. 2004;20 16:2878-9. doi:10.1093/bioinformatics/bth315.
74. Stanke M, Keller O, Gunduz I, Hayes A, Waack S and Morgenstern B. AUGUSTUS: ab initio prediction of alternative transcripts. Nucleic acids research. 2006;34:W435-W9. doi:10.1093/nar/gkl200.
75. Tribolium Genome Sequencing C, Richards S, Gibbs RA, Weinstock GM, Brown SJ, Denell R, et al. The genome of the model beetle and pest *Tribolium castaneum*. Nature. 2008;452 7190:949-55. doi:10.1038/nature06784.
76. Adams MD, Celniker SE, Holt RA, Evans CA, Gocayne JD, Amanatides PG, et al. The genome sequence of *Drosophila melanogaster*. Science. 2000;287 5461:2185-95.
77. Duan J, Li R, Cheng D, Fan W, Zha X, Cheng T, et al. SilkDB v2.0: a platform for silkworm (*Bombyx mori*) genome biology. Nucleic acids research. 2010;38 Database issue:D453-6. doi:10.1093/nar/gkp801.
78. Pearce SL, Clarke DF, East PD, Elfekih S, Gordon KHJ, Jermiin LS, et al. Genomic innovations, transcriptional plasticity and gene loss underlying the evolution and

divergence of two highly polyphagous and invasive *Helicoverpa* pest species. BMC biology. 2017;15 1:63. doi:10.1186/s12915-017-0402-6.

79. Altschul SF, Madden TL, Schaffer AA, Zhang JH, Zhang Z, Miller W, et al. Gapped BLAST and PSI-BLAST: a new generation of protein database search programs. Nucleic acids research. 1997;25 17:3389-402. doi:10.1093/nar/25.17.3389.

80. Birney E, Clamp M and Durbin R. GeneWise and genomewise. Genome research. 2004;14 5:988-95. doi:10.1101/gr.1865504.

81. Haas BJ, Salzberg SL, Zhu W, Pertea M, Allen JE, Orvis J, et al. Automated eukaryotic gene structure annotation using EVidenceModeler and the program to assemble spliced alignments. Genome Biology. 2008;9 1 doi:10.1186/Gb-2008-9-1-R7.

82. Jones P, Binns D, Chang H-Y, Fraser M, Li W, McAnulla C, et al. InterProScan 5: genome-scale protein function classification. Bioinformatics. 2014;30 9:1236-40.

83. Li L, Stoeckert CJ and Roos DS. OrthoMCL: identification of ortholog groups for eukaryotic genomes. Genome Research. 2003;13 9:2178-89.

84. De Bie T, Cristianini N, Demuth JP and Hahn MW. CAFE: a computational tool for the study of gene family evolution. Bioinformatics. 2006;22 10:1269-71.

85. Loytynoja A and Goldman N. An algorithm for progressive multiple alignment of sequences with insertions. Proceedings of the National Academy of Sciences of the United States of America. 2005;102 30:10557-62. doi:10.1073/pnas.0409137102.

86. Stamatakis A. RAxML version 8: a tool for phylogenetic analysis and post-analysis of large phylogenies. Bioinformatics. 2014;30 9:1312-3. doi:10.1093/bioinformatics/btu033.

87. Mirarab S, Reaz R, Bayzid MS, Zimmermann T, Swenson MS and Warnow T. ASTRAL: genome-scale coalescent-based species tree estimation. Bioinformatics. 2014;30 17:I541-I8. doi:10.1093/bioinformatics/btu462.

88. Yang ZH. PAML 4: Phylogenetic analysis by maximum likelihood. Molecular Biology and Evolution. 2007;24 8:1586-91. doi:10.1093/molbev/msm088.

89. Kumar S, Stecher G, Suleski M and Hedges SB. TimeTree: A Resource for Timelines, Timetrees, and Divergence Times. Molecular biology and evolution. 2017;34 7:1812-9. doi:10.1093/molbev/msx116.

90. Zakharov EV, Caterino MS and Sperling FA. Molecular phylogeny, historical biogeography, and divergence time estimates for swallowtail butterflies of the genus *Papilio* (Lepidoptera: Papilionidae). Systematic biology. 2004;53 2:278-98.

91. Dupuis JR and Sperling FA. Repeated reticulate evolution in North American *Papilio machaon* group swallowtail butterflies. PloS one. 2015;10 10:e0141882.

92. Espeland M, Breinholt J, Willmott KR, Warren AD, Vila R, Toussaint EFA, et al. A Comprehensive and Dated Phylogenomic Analysis of Butterflies. Current Biology. 2018;28 5:770-+. doi:10.1016/j.cub.2018.01.061.

93. Li H and Durbin R. Inference of human population history from individual whole-genome sequences. Nature. 2011;475 7357:493-U84. doi:10.1038/nature10231.

94. Sanderson MJ. r8s: inferring absolute rates of molecular evolution and divergence times in the absence of a molecular clock. Bioinformatics. 2003;19 2:301-2. doi:DOI 10.1093/bioinformatics/19.2.301.

95. Li H, Handsaker B, Wysoker A, Fennell T, Ruan J, Homer N, et al. The Sequence Alignment/Map format and SAMtools. Bioinformatics. 2009;25 16:2078-9. doi:10.1093/bioinformatics/btp352.

96. Zhang W, Westerman E, Nitzany E, Palmer S and Kronforst MR. Tracing the origin and evolution of supergene mimicry in butterflies. Nature Communications. 2017;8 1:1269. doi:10.1038/s41467-017-01370-1.

97. Lu S; Yang J; Dai X; Xie F; He J; Dong Z; Mao J; Liu G; Chang Z; Zhao R; Wan W; Zhang R; Li Y; Wang W; Li X (2019): Supporting data for "Chromosomal-level reference genome of Chinese peacock butterfly (*Papilio bianor*) based on third-

generation DNA sequencing and Hi-C analysis" GigaScience Database. http://dx.doi.org/10.5524/100653

Table 1: Comparison of quality and composition of different butterfly genomes.

Family	Species	Genome size (Mb)	Genome size without gap (Mb)	Heterozygosity [a] (%)	Scaffold N50 (kb)	BUSCO [b] (%)	*De novo* assembled transcripts [a] (%)	GC content (%)	Repeat (%)	Exon (%)	Intron (%)	Number of proteins (k)
Papilionidae	*Papilio bianor*	**421**	**421**	**1.8**	**13120**	**96.3**	NA	**36.6**	**55.3**	**5.05**	**27.44**	**15.4**
	Papilio xuthus	244	238	1.0	6199	97.6	NA	33.8	22.4	8.59	45.50	13.1
	Papilio machaon	281	266	1.2	1150	95.5	98	32.3	22.3	7.37	30.36	15.5
	Papilio polytes	227	218	NA	3672	91.8	NA	34.0	23.8	12.97	48.58	12.2
	Papilio memnon	233	219	NA	5457	96.6	NA	32.8	22.5	11.31	43.17	12.4
	Papilio glaucus	375	361	2.3	231	95.5	98	35.4	22.0	5.07	25.60	15.7
Hesperiidae	*Achalarus lyciades*	567	536	1.5	558	97.3	98	35.3	25.0	3.57	28.40	15.9
	Lerema accius	298	290	1.5	525	95.1	98	34.4	15.5	6.96	31.60	17.4
	Megathymus ursus violae	429	427	0.1	4153	98.3	99	34.7	25.8	4.59	30.90	14.1
Pieridae	*Pieris rapae*	246	243	1.5	617	98.0	99	32.7	22.7	7.91	33.30	13.2
	Phoebis sennae	406	347	1.2	257	97.7	97	39.0	17.2	6.20	25.50	16.5
Nymphalidae	*Danaus plexippus*	249	242	0.6	716	98.0	96	31.6	16.3	8.40	28.10	15.1
	Heliconius melpomene	274	270	NA	194	95.6	NA	32.8	24.9	6.38	25.40	12.8
	Melitaea cinxia	390	361	NA	119	83.0	97	32.6	27.5	4.34	31.20	16.7
	Bicyclus anynana	475	470	NA	638	97.6	NA	36.5	25.8	4.73	38.36	22.6
Riodinidae	*Calephelis nemesis*	809	783	0.5	206	95.6	99	34.9	34.8	2.25	19.60	15.4
	Calephelis virginiensis	855	824	1.3	175	93.9	99	35.0	38.8	2.17	20.50	15.6
Lycaenidae	*Calycopis cecrops*	729	689	1.2	233	95.5	96	37.1	34.0	3.11	24.00	16.5

[a] The heterozygosity of *P. bianor*, *P. machaon* and *P. xuthus* were calculated based on *k-mer* distribution analysis. The heterozygosity values of others (*P. glaucus*, *A. lyciades*, *L. accius*, *M. ursus violae*, *P. rapae*, *P. sennae*, *D. plexippus*, *C. nemesis*, *C. virginiensis*, *C. cecrops*) were estimated using the Genome Analysis Toolkit (GATK).

[b] BUSCO is calculated in this study.

NA: not available in the referenced citation.

Figure legends

Figure 1. Characterization of *Papilio bianor*. (a) Female adult of *P. bianor.* Shown from left to right are: (1) dorsal view, (2) ventral view. (scales = 20.0 mm; Photo by Zhiwei Dong) **(b)** Heatmap of chromosomal interactions. Each chromosome is framed with blue block, and each scaffold is framed with green block. **(c)** Circos plot of *P. bianor* chromosome-level reference genome with the previously released *Papilio xuthus* genome (obtained from Chinese group) [9]. Shown from the outermost to innermost are: (1) gene density, (2) repeat element density, (3) GC content, and (4) syntenic regions with *P. xuthus* (left).

Figure 2. Genomic analysis of *Papilio bianor*. (a) Breakdown of the whole-genome assemblies into different functional classes in *Papilio*. **(b)** Venn diagram of the shared gene families of *Papilio.* **(c)** Maximum Likelihood (ML) phylogenetic tree of Papilionoidea constructed by the concatenated alignment of 1378 one to one single copy orthologue genes. The numbers in the square brackets on the nodes are the 95% confidence intervals of divergence time. The red dots are fossil evidence downloaded from the TIMETREE website and the black dots are inferred time obtained from the TIMETREE website. Both of them were used to calibrate divergent time. **(d)** The dynamic changes of the effective population size are plotted using PSMC software, with 100 bootstrap replicates to test the robust variations. The parameter “g” represents the generation time in years, and the parameter “μ” means the per generation mutation rate. Pb: *Papilio bianor*; Pgl: *Papilio glaucus*; Pma: *Papilio machaon*; Pme: *Papilio memnon*; Ppol: *Papilio polytes*; Pxu: *Papilio xuthus*.

Figure 1

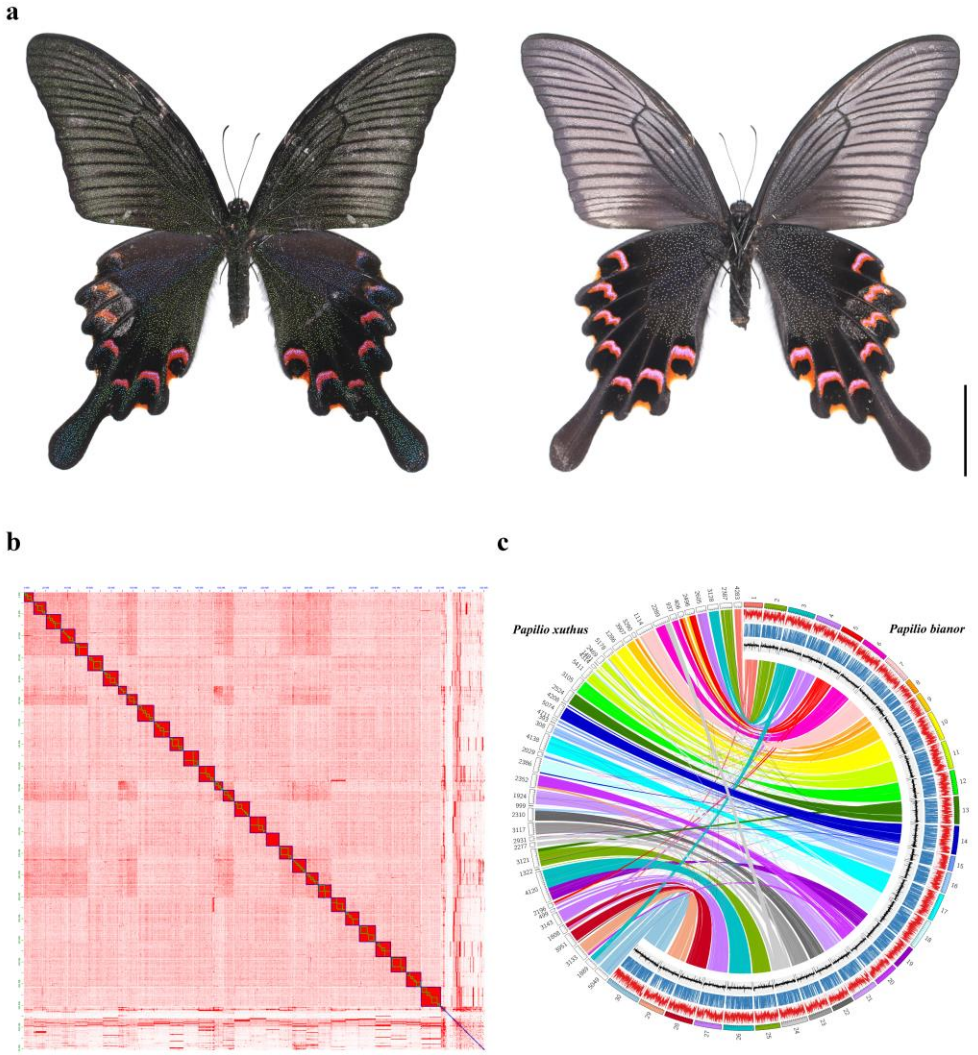

Figure 2

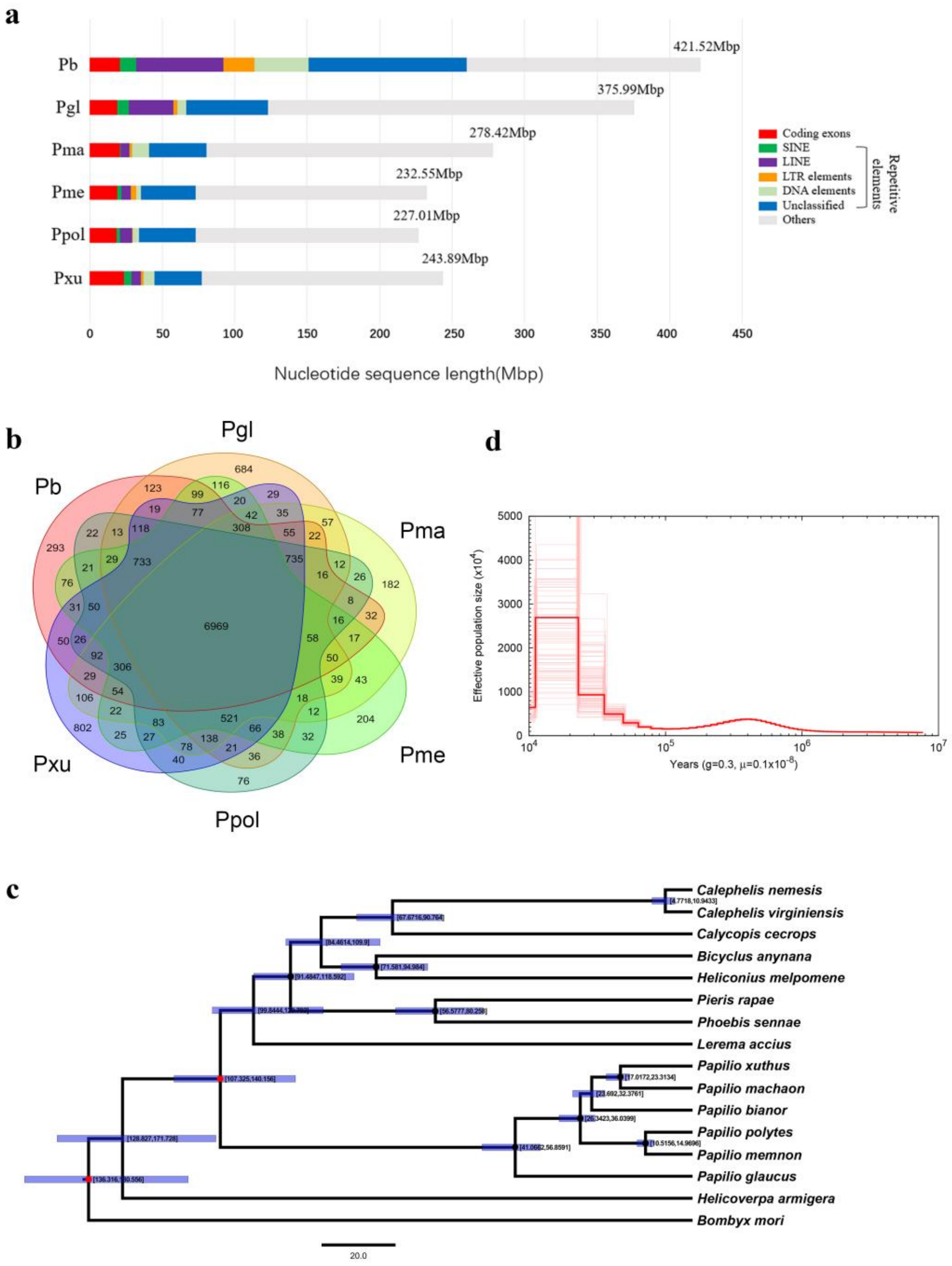
a
Pb
Pgl
Pma
Pme
Ppol
Pxu
421.52Mbp
375.99Mbp
278.42Mbp
232.55Mbp
227.01Mbp
243.89Mbp
Coding exons
SINE
LINE
LTR elements
DNA elements
Unclassified
Others
Repetitive elements
0
50
100
150
200
250
300
350
400
450
Nucleotide sequence length(Mbp)
b
Pgl
Pb
Pma
Pxu
Pme
Ppol
6969
d
Effective population size (x10⁴)
Years (g=0.3, μ=0.1x10⁻⁸)
c
Calephelis nemesis
Calephelis virginiensis
Calycopis cecrops
Bicyclus anynana
Heliconius melpomene
Pieris rapae
Phoebis sennae
Lerema accius
Papilio xuthus
Papilio machaon
Papilio bianor
Papilio polytes
Papilio memnon
Papilio glaucus
Helicoverpa armigera
Bombyx mori
20.0

Additional files

Figure S1: *K-mer* (k=17) distribution in *Papilio bianor* genome. The first peak (depth=26) is a heterozygous peak, which is higher than the main peak (depth=53), suggesting the *P. bianor* genome is highly heterozygous. The x-axis is depth (×); the y-axis is the proportion which represents the frequency at that depth divide by the total frequency of all the depth.

Figure S2: Heatmap of per-chromosomal interactions. Each scaffold is framed with green block.

Figure S3: The coverage distribution of Illumina reads mapping to *Papilio bianor* genome. The histogram follows a normal distribution, indicating few heterozygous regions in assembled genome.

Figure S4: The coverage distribution of PacBio reads mapping to *Papilio bianor* genome. The histogram follows a normal distribution, indicating few heterozygous regions in assembled genome.

Figure S5: The statistics of annotated protein-coding genes of *Papilio.* (a) mRNA length, (b) coding sequence (CDS) length, (c) exon length, (d) intron length, (e) exon number. The x-axis represents length or number and the y-axis represents the density of genes. Pb: *Papilio bianor*; Pgl: *Papilio glaucus*; Pma: *Papilio machaon*; Pme: *Papilio memnon*; Ppol: *Papilio polytes*; Pxu: *Papilio xuthus*.

Figure S6. Maximum Likelihood (ML) phylogenetic tree of Papilionoidea constructed by merging each of the single copy orthologs.

Table S1: The statistics of sequencing data generated for *Papilio bianor* genome. The sequencing depth was calculated by the assembled genome size.

Table S2: Genome size estimation of *Papilio bianor* with *k-mer* distribution analysis using k=17.

Table S3: The statistics of assembled chromosome-level genome of *Papilio bianor*. The Hi-C data were filtered by HiC-Pro software. In total, 6,690,421 pairs of reads, accounting 68.04% of the total Hi-C data, were used in downstream analysis.

Table S4: The contiguity assessment of genome assembly of *Papilio bianor*.

Table S5: The quality evaluation of assembled genome of *Papilio bianor* by BUSCO software with insecta_odb9.

Table S6: The statistics of mapping ratio of Illumina reads to *Papilio bianor* assembled genome.

Table S7: The statistics of mapping ratio of PacBio reads to *Papilio bianor* assembled genome.

Table S8: The statistics of the annotated repeat sequences in *Papilio bianor* genome.

Table S9: The statistics of the TE contents in *Papilio bianor* genome.

Table S10: The statistics of predicted protein-coding genes in *Papilio bianor* genome.

Table S11: The statistics of gene function annotation in *Papilio bianor* genome.

Table S12: The GO term enrichment of expanded gene families in *Papilio bianor* genome.

Table S13: The GO term enrichment of contracted gene families in *Papilio bianor* genome.

Figure 2

Click here to access/download;Figure;GIGA-D-19-00120_Figure 2.jpg

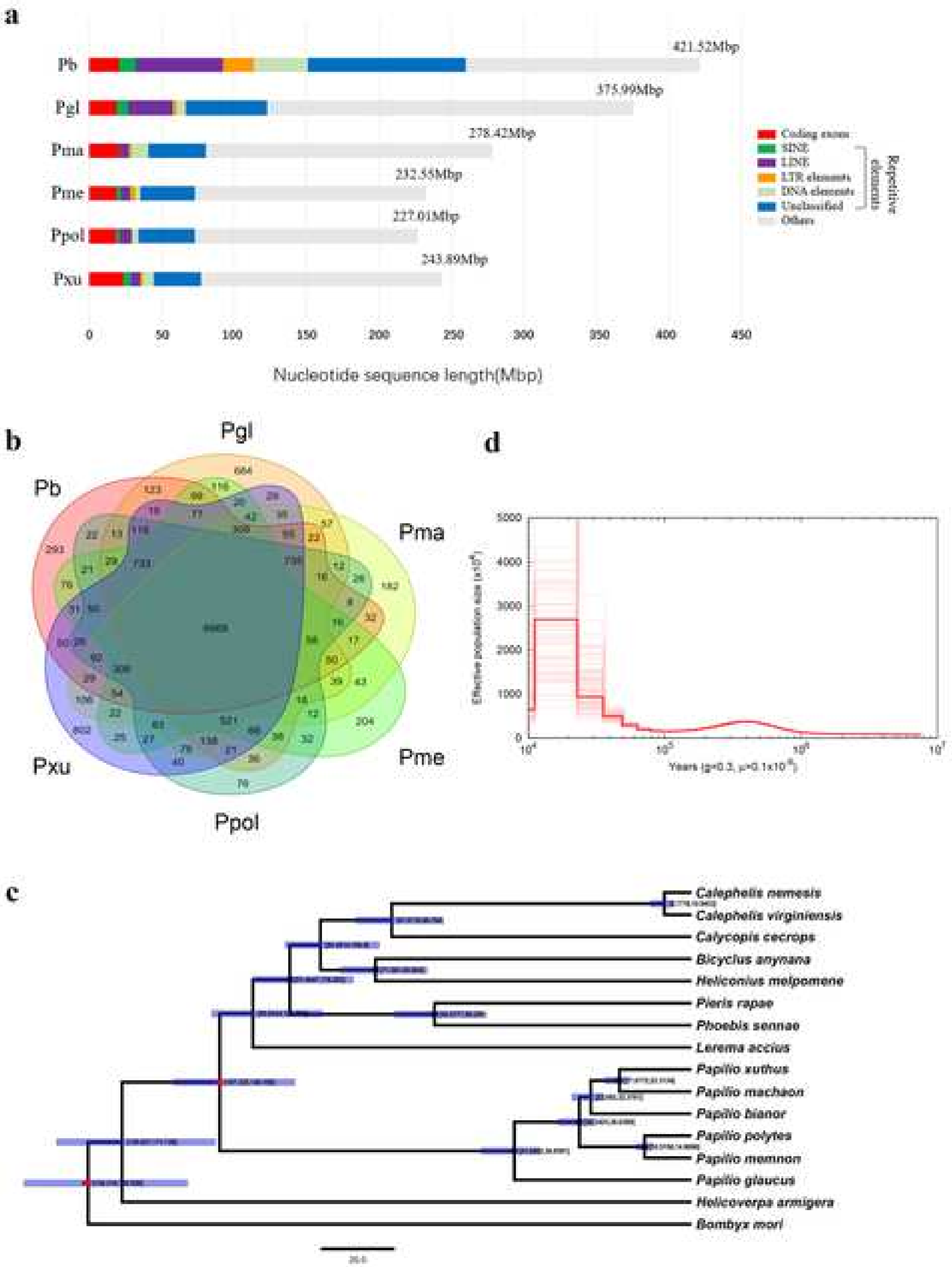

Figure 1

Click here to access/download;Figure;GIGA-D-19-00120_Figure 1.jpg

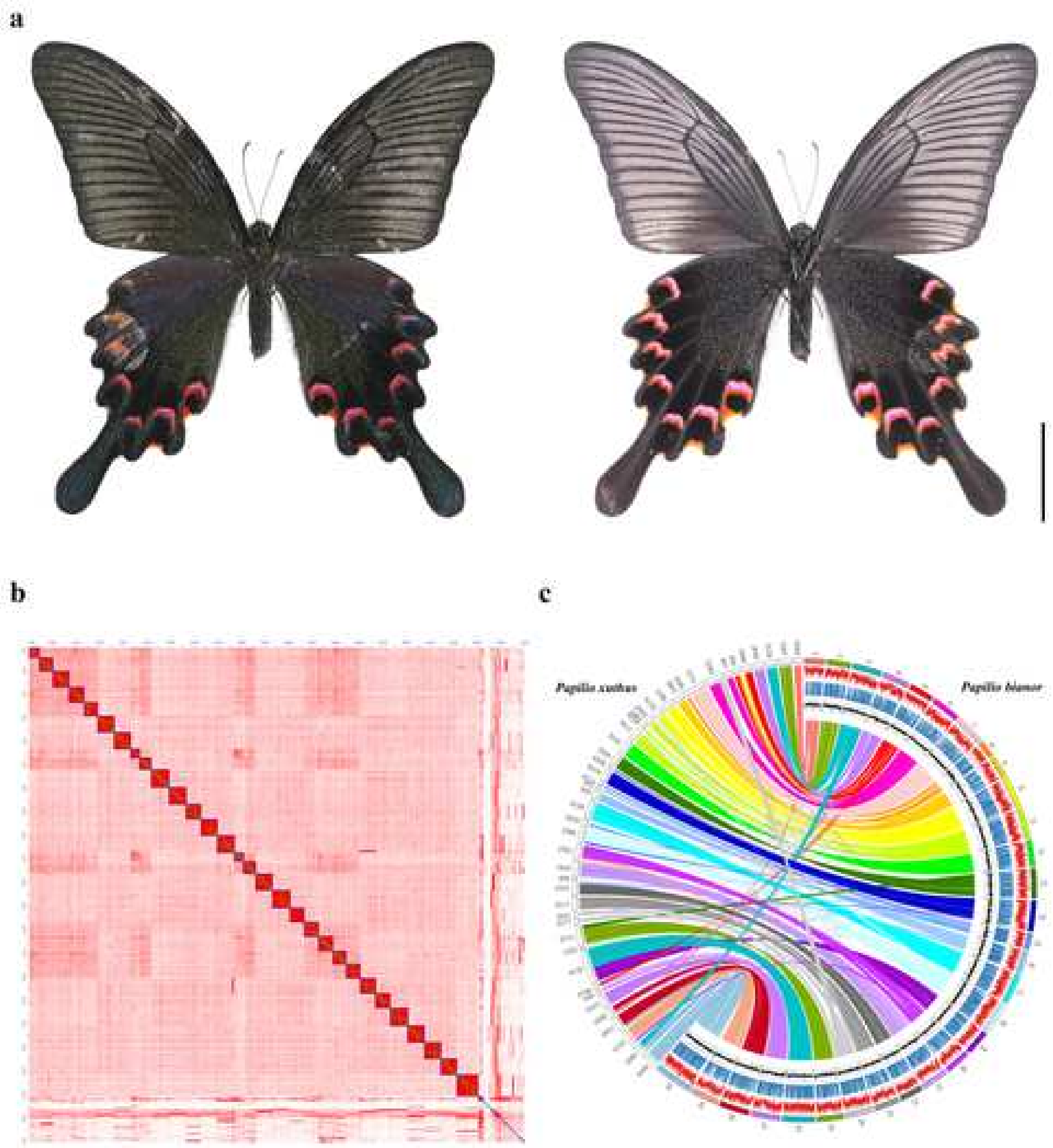

Click here to access/download
Supplementary Material
GIGA-D-19-00120_Pb-Genome_Suplementary files_20190807.docx

Supplementary Figure 1

Click here to access/download
Supplementary Material
GIGA-D-19-00120_Figure S1.jpg

Supplementary Figure 2

Click here to access/download
Supplementary Material
GIGA-D-19-00120_Figure S2.jpg

Supplementary Figure 3

Click here to access/download

Supplementary Material

GIGA-D-19-00120_Figure S3.png

Supplementary Figure 4

Click here to access/download

Supplementary Material

GIGA-D-19-00120_Figure S4.png

Supplementary Figure 5

Click here to access/download
Supplementary Material
GIGA-D-19-00120_Figure S5.jpg

Supplementary Figure 6

Click here to access/download
Supplementary Material
GIGA-D-19-00120_Figure S6.jpg

Response to Reviewers letter

Click here to access/download

Supplementary Material

GIGA-D-19-00120_Response to Reviewers letter_20190807.docx
