# Supplementary material for: Chromosomal-level reference genome of Chinese peacock butterfly (Papilio bianor) based on third-generation DNA sequencing and Hi-C analysis
Source: Gigascience. 2019 Nov 4;8(11):giz128. doi: 10.1093/gigascience/giz128 (PMC6827417; doi:10.1093/gigascience/giz128)
Supplement: giz128_Supplemental_Files [file giz128_supplemental_files.zip › supplement data/GIGA-D-19-00120_Pb-Genome_Suplementary files_20190807.docx]

**Additional files**


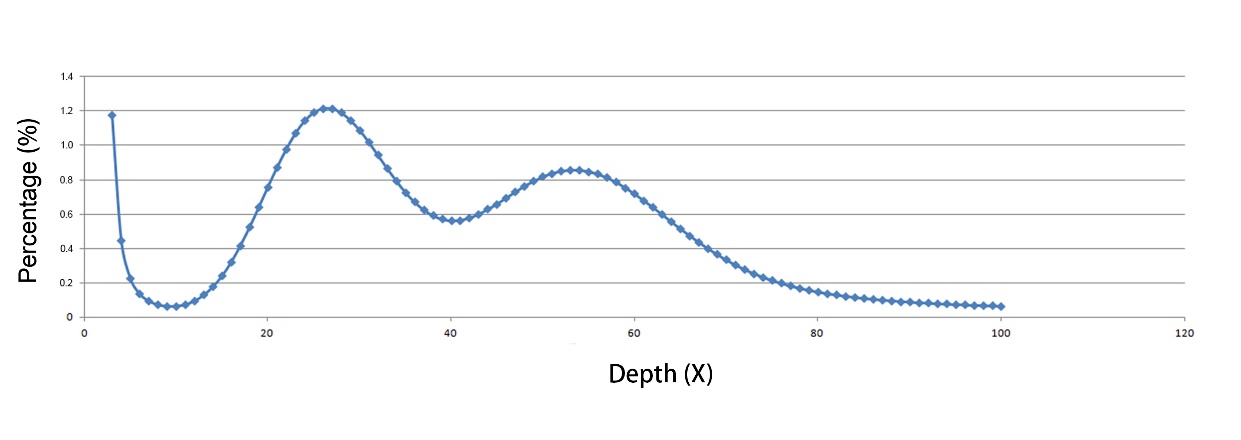


**Figure S1. *K-mer* (k=17) distribution in *Papilio*** ***bianor* genome.** The first peak (depth=26) is a heterozygous peak, which is higher than the main peak (depth=53), suggesting the *P. bianor* genome is highly heterozygous. The x-axis is depth (×); the y-axis is the proportion which represents the frequency at that depth divide by the total frequency of all the depth.


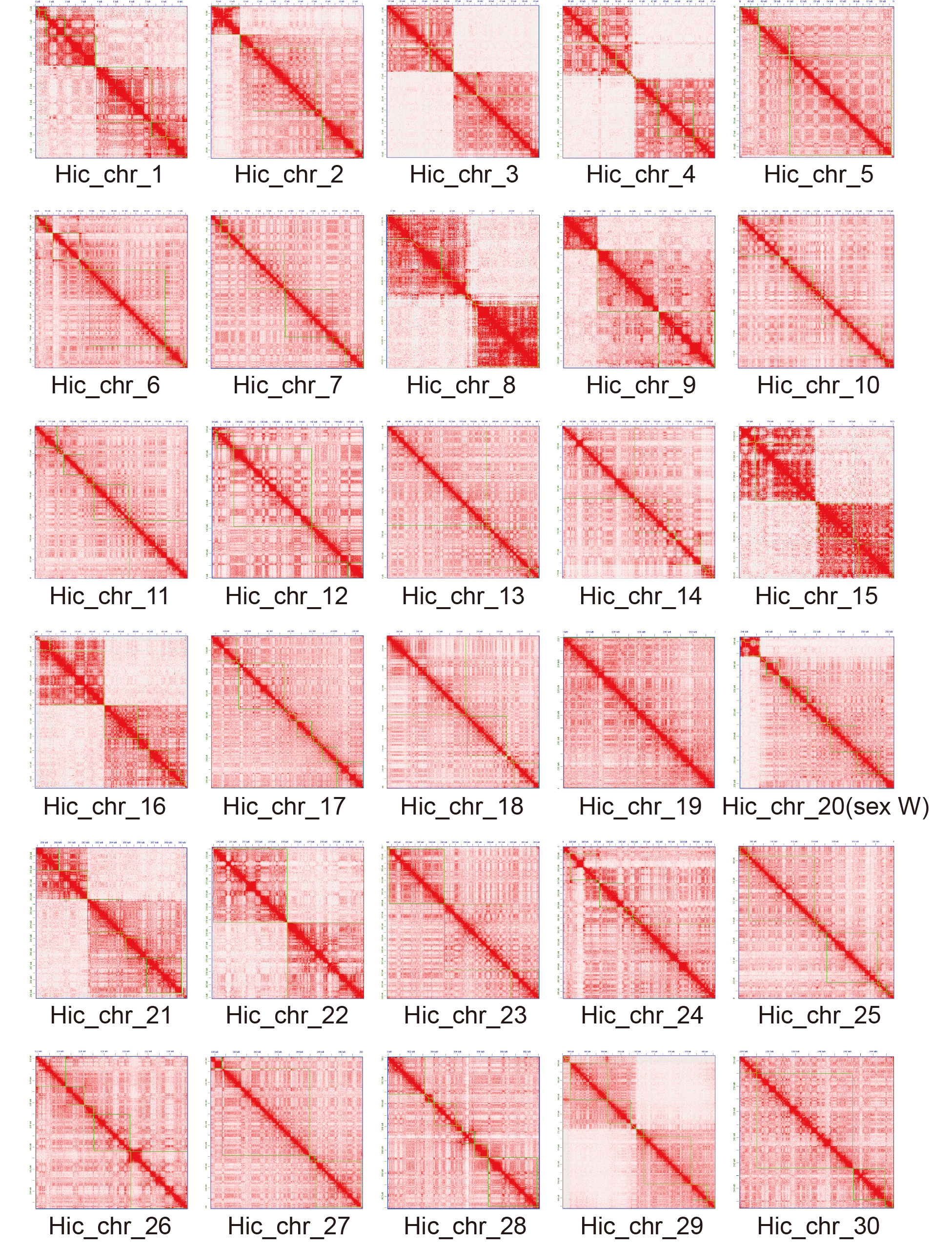


**Figure S2.** Heatmap of per-chromosomal interactions. Each scaffold is framed with green block.


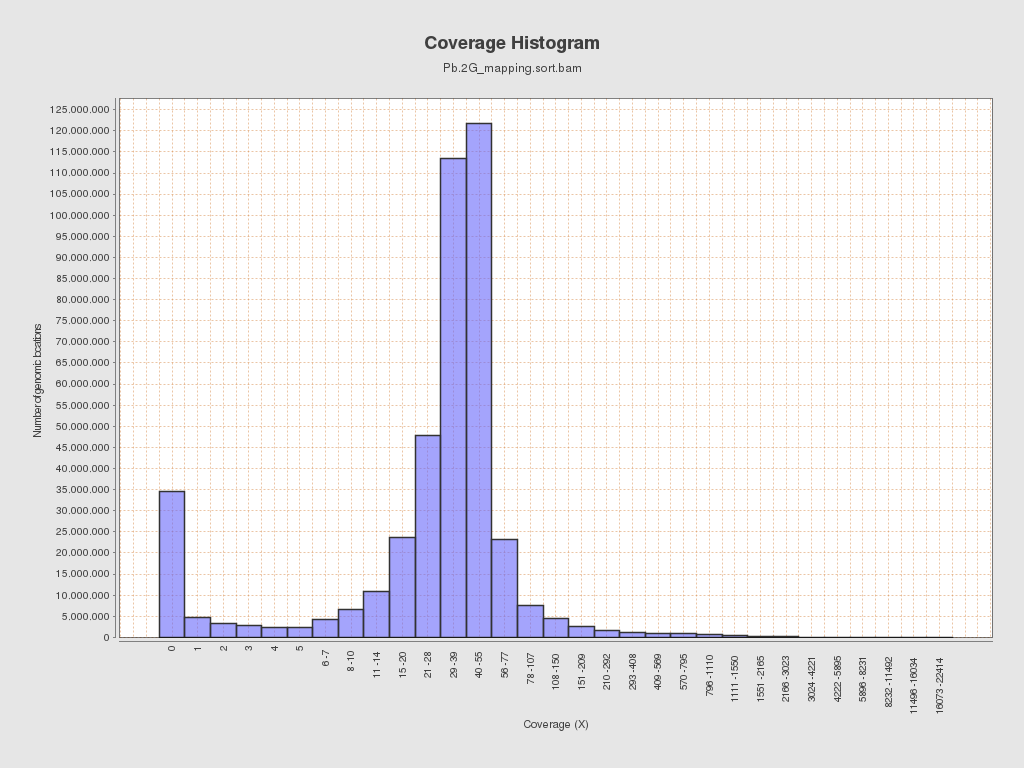


**Figure S3. The coverage distribution of Illumina reads mapping to *Papilio bianor* genome.** The histogram follows a normal distribution, indicating few heterozygous regions in assembled genome.


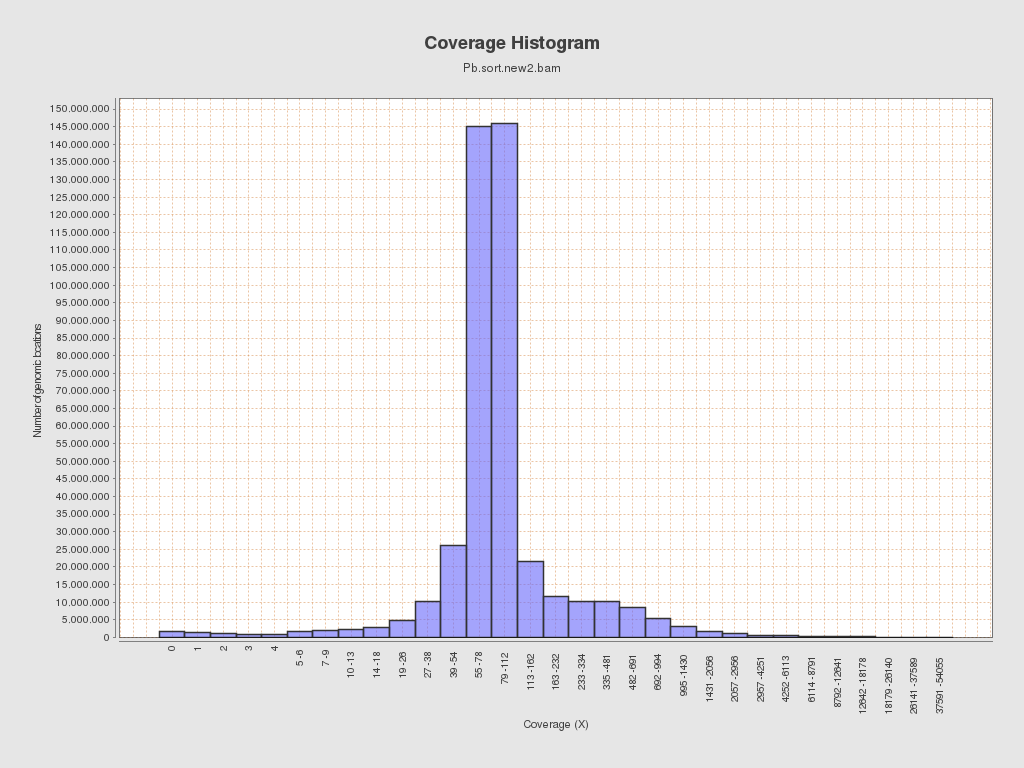


**Figure S4. The coverage distribution of PacBio reads mapping to *Papilio bianor* genome.** The histogram follows a normal distribution, indicating few heterozygous regions in assembled genome.


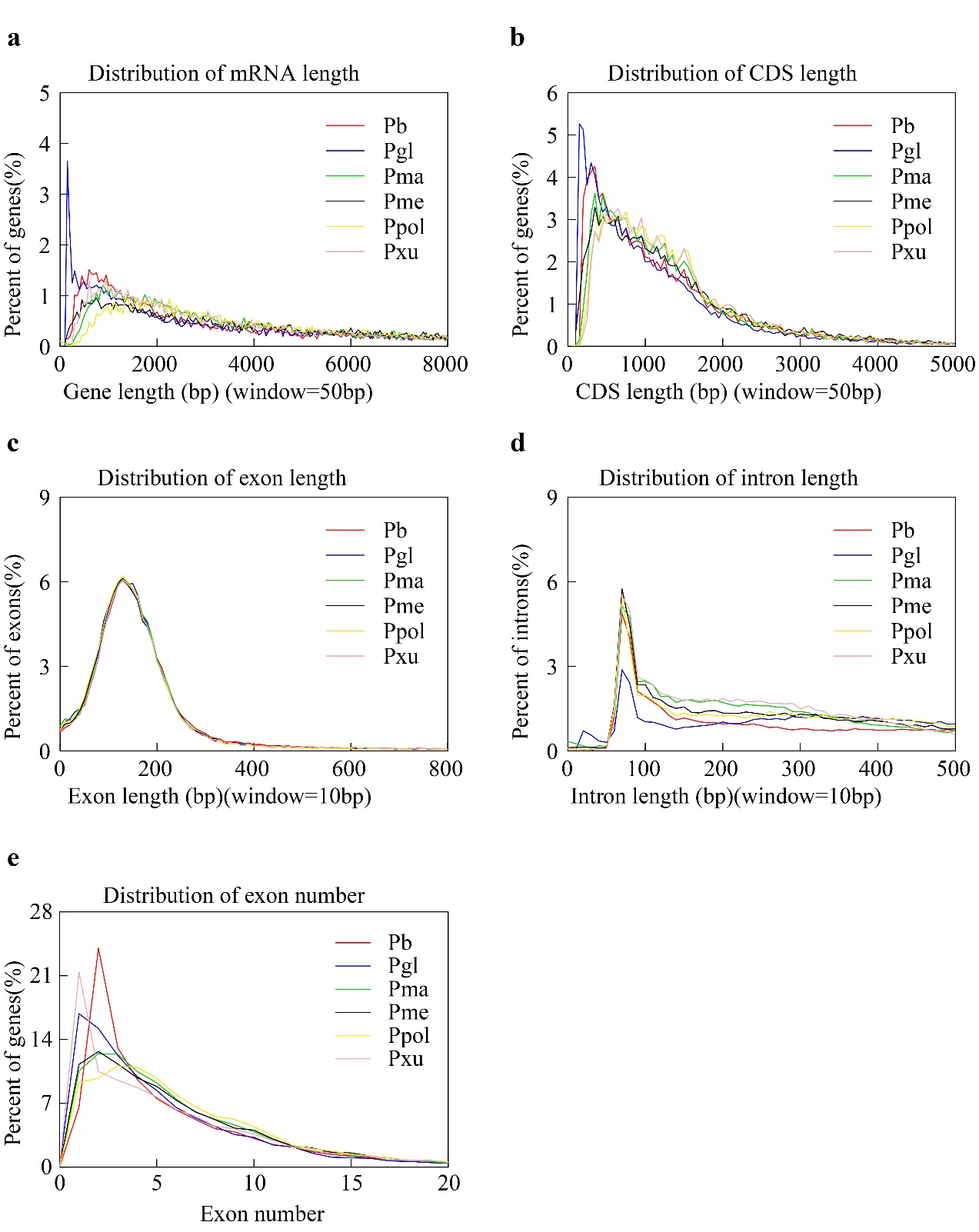


**Figure S5. The statistics of annotated protein-coding genes of *Papilio*.** (a) mRNA length, (b) coding sequence (CDS) length, (c) exon length, (d) intron length, (e) exon number. The x-axis represents length or number and the y-axis represents the density of genes. Pb: *Papilio bianor*; Pgl: *Papilio glaucus*; Pma: *Papilio machaon*; Pme: *Papilio memnon*; Ppol: *Papilio polytes*; Pxu: *Papilio xuthus*.


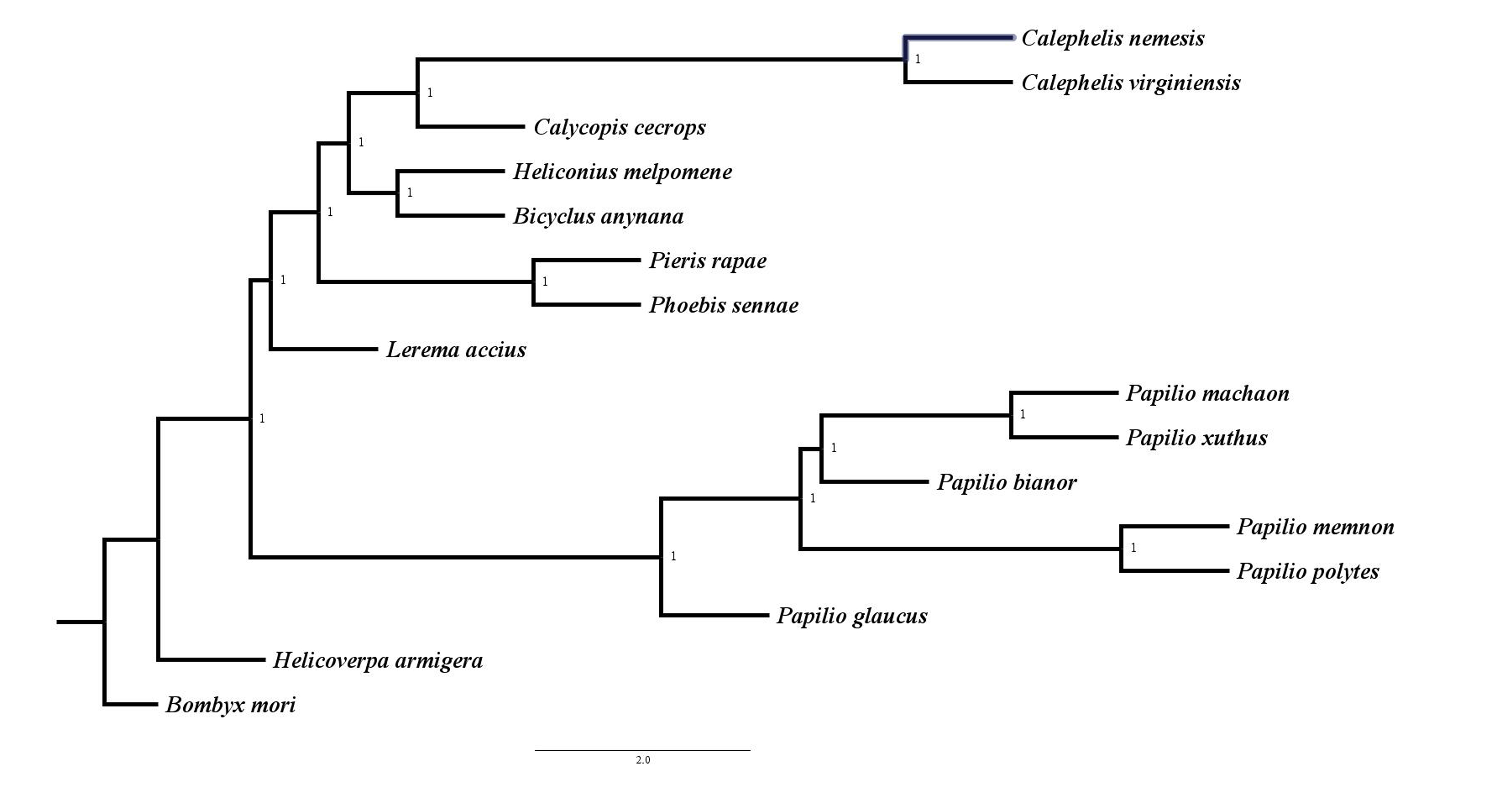


**Figure S6. Maximum Likelihood (ML) phylogenetic tree of Papilionoidea constructed by merging each of the single copy orthologs.**

**Table S1. The statistics of sequencing data generated for *Papilio bianor* genome.**

| **Platform** | **Library size (bp)** | **Data size (Gb)** | **depth (×)** | **Application** |
| --- | --- | --- | --- | --- |
| Illumina HiSeq2000 | 150 | 16.45 | 39 | Genome survey and genomic base correction |
| Illumina HiSeq2000 | 500 | 28.42 | 68 | Genome survey and genomic base correction |
| PacBio RSII | 20,000 | 43.19 | 103 | Genome assembly |
| Illumina HiSeq X Ten | 150 | 63.05 | 150 | Chromosome construction |
| Illumina HiSeq X Ten | 150 | 12.06 | 29 | Chromosome construction |

The sequencing depth was calculated by the assembled genome size.

**Table S2. Genome size estimation of *Papilio bianor* with *k-mer* distribution analysis using k=17.**

| **k** | **K-mer_Num** | **Peak_Depth** | **Repeat** | **Heterozygosity** | **Genome size (bp)** | **Used bases (bp)** | **Used reads** | **×** |
| --- | --- | --- | --- | --- | --- | --- | --- | --- |
| 17 | 26,657,355,060 | 53 | 0.583 | 0.018 | 496,050,178 | 31,734,946,500 | 317,349,465 | 31 |

**Table S3. The statistics of assembled chromosome-level genome of *Papilio bianor*.**

| **Super scaffold** | **Length (bp)/ Percentage (%)** | **Contig number/ Percentage (%)** |
| --- | --- | --- |
| Hic_chr_1 | 9,469,303 | 5 |
| Hic_chr_2 | 11,161,103 | 9 |
| Hic_chr_3 | 13,703,452 | 7 |
| Hic_chr_4 | 12,865,593 | 19 |
| Hic_chr_5 | 11,694,500 | 8 |
| Hic_chr_6 | 13,832,328 | 3 |
| Hic_chr_7 | 14,008,696 | 6 |
| Hic_chr_8 | 7,736,502 | 8 |
| Hic_chr_9 | 10,074,838 | 4 |
| Hic_chr_10 | 14,936,136 | 6 |
| Hic_chr_11 | 14,416,367 | 2 |
| Hic_chr_12 | 12,260,247 | 5 |
| Hic_chr_13 | 13,999,980 | 2 |
| Hic_chr_14 | 14,450,437 | 11 |
| Hic_chr_15 | 7,580,000 | 7 |
| Hic_chr_16 | 10,568,879 | 15 |
| Hic_chr_17 | 14,134,073 | 4 |
| Hic_chr_18 | 15,206,575 | 2 |
| Hic_chr_19 | 11,737,462 | 2 |
| Hic_chr_20 | 11,935,061 | 5 |
| Hic_chr_21 | 12,796,568 | 10 |
| Hic_chr_22 | 10,994,142 | 11 |
| Hic_chr_23 | 11,737,423 | 4 |
| Hic_chr_24 | 13,120,256 | 4 |
| Hic_chr_25 | 12,323,442 | 1 |
| Hic_chr_26 | 15,238,437 | 6 |
| Hic_chr_27 | 13,812,743 | 5 |
| Hic_chr_28 | 14,381,968 | 6 |
| Hic_chr_29 | 13,127,372 | 8 |
| Hic_chr_30 | 18,214,045 | 13 |
| Unanchored | 40,006,809 | 1486 |
| Length of Scaffold assembled into chromosomes | 381,517,928 | -- |
| Total length of assembled genome | 421,524,737 | -- |
| Chromosome/total | 90.51% | 11.76% |

The Hi-C data were filtered by HiC-Pro software. In total, 6,690,421 pairs of reads, accounting 68.04% of the total Hi-C data, were used in downstream analysis.

**Table S4. The contiguity assessment of genome assembly of *Papilio bianor*.**

| **Type** | **Contig** | **Scaffold** |
| --- | --- | --- |
| Total number | 1,684 | 1,710 |
| Total length (bp) | 421,234,737 | 421,524,737 |
| N10 length (bp) | 12,993,832 | 15,206,575 |
| N20 length (bp) | 10,621,897 | 14,416,367 |
| N30 length (bp) | 8,966,101 | 14,008,696 |
| N40 length (bp) | 6,893,074 | 13,812,743 |
| N50 length (bp) | 5,762,537 | 13,120,256 |
| N60 length (bp) | 4,111,982 | 12,323,442 |
| N70 length (bp) | 2,777,716 | 11,737,423 |
| N80 length (bp) | 1,914,696 | 10,568,879 |
| N90 length (bp) | 330,040 | 7,580,000 |
| Max length (bp) | 15,785,465 | 18,214,045 |
| GC content | 36.58% | 36.56% |

**Table S5. The quality evaluation of assembled genome of *Papilio bianor* by BUSCO software with** **insecta_odb9.**

| **Types of BUSCOs** | **Count** | **Ratio (%)** |
| --- | --- | --- |
| Complete BUSCOs | 1,597 | 96.30 |
| Complete and single-copy BUSCOs | 1,585 | 95.60 |
| Complete and duplicated BUSCOs | 12 | 0.70 |
| Fragmented BUSCOs | 10 | 0.60 |
| Missing BUSCOs | 51 | 3.10 |

**Table S6. The statistics of mapping ratio of Illumina reads to *Papilio bianor* assembled genome.**

| **Library Type** | **Total reads** | **Mapped reads** | **Mapped reads ratio** | **Properly paired** | **Properly paired ratio** |
| --- | --- | --- | --- | --- | --- |
| Illumina HiSeq2000 | 211,033,840 | 203,250,923 | 96.31% | 179,935,948 | 86.11% |

**Table S7. The statistics of mapping ratio of PacBio reads to *Papilio bianor* assembled genome.**

| **Term** | **Reads number** | **Percentage (%)** |
| --- | --- | --- |
| MQ>=0 | 3,747,145 | 96.86 |
| MQ>=5 | 3,691,420 | 95.41 |
| MQ>=10 | 3,671,677 | 94.90 |
| MQ>=30 | 3,629,307 | 93.81 |
| MQ>=60 | 3,609,601 | 93.30 |
| **Total** | 3,868,813 | -- |

**Table S8. The statistics of the annotated repeat sequences in *Papilio bianor* genome.**

| **Type** | **Repeat Size (bp)** | **% of genome** |
| --- | --- | --- |
| Trf | 27,742,655 | 6.58 |
| Repeatmasker | 15,457,063 | 3.67 |
| Proteinmask | 43,856,806 | 10.40 |
| De novo | 215,640,344 | 51.16 |
| **Total** | 233,088,461 | 55.30 |

**Table S9. The statistics of the TE contents in *Papilio bianor* genome.**

|  | Repbase TEs | | TE proteins | | *De novo* | | Combined TEs | |
| --- | --- | --- | --- | --- | --- | --- | --- | --- |
|  | Length (bp) | % in genome | Length (bp) | % in genome | Length (bp) | % in genome | Length (bp) | % in genome |
| DNA | 6,419,163 | 1.52 | 5,721,326 | 1.36 | 35,064,597 | 8.32 | 37,138,138 | 8.81 |
| LINE | 5,185,080 | 1.23 | 24,655,425 | 5.85 | 53,379,878 | 12.66 | 59,921,241 | 14.22 |
| SINE | 113,554 | 0.03 | 0 | 0.00 | 11,191,343 | 2.65 | 11,253,476 | 2.67 |
| LTR | 4,760,315 | 1.13 | 13,548,792 | 3.21 | 15,118,936 | 3.59 | 21,464,046 | 5.09 |
| Other | 0 | 0.00 | 0 | 0.00 | 0 | 0.00 | 0 | 0.00 |
| Unknown | 0 | 0.00 | 0 | 0.00 | 109,180,121 | 25.90 | 109,180,121 | 25.90 |
| **Total** | 15,457,063 | 3.67 | 43,856,806 | 10.40 | 215,045,545 | 51.02 | 221,781,601 | 52.61 |

**Table S10. The statistics of predicted protein-coding genes in *Papilio bianor* genome.**

| **Gene Set** | | **Number** | **Average Gene Length (bp)** | **Average CDS Length (bp)** | **Average Exon Number per Gene** | **Average Exon Length (bp)** | **Average Intron Length (bp)** |
| --- | --- | --- | --- | --- | --- | --- | --- |
| *De novo* | AUGUSTUS | 17120 | 5080.62 | 1233.69 | 5.82 | 211.85 | 797.56 |
|  | GENSCAN | 18450 | 15056.04 | 1203.64 | 5.81 | 207.06 | 2878.18 |
|  | GLIMMERHMM | 44812 | 8225.12 | 639.77 | 4.00 | 159.77 | 2524.75 |
|  | SNAP | 18450 | 4119.06 | 601.82 | 4.21 | 143.03 | 1096.56 |
| Homolog | *Tribolium castaneum* | 10147 | 7218.81 | 1232.74 | 5.52 | 223.32 | 1324.36 |
|  | *Drosophila melanogaster* | 8770 | 6758.41 | 1169.01 | 5.40 | 216.45 | 1270.09 |
|  | *Bombyx mori* | 14424 | 7975.82 | 1313.55 | 5.77 | 227.65 | 1396.71 |
|  | *Helicoverpa armigera* | 13541 | 8684.09 | 1438.84 | 6.16 | 233.74 | 1405.30 |
|  | *Papilio polytes* | 13347 | 9141.23 | 1401.51 | 6.28 | 223.33 | 1467.09 |
|  | *Papilio xuthus* | 15088 | 8985.28 | 1532.53 | 6.53 | 234.77 | 1348.21 |
|  | *Heliconius melpomene* | 18713 | 5785.07 | 1170.01 | 4.87 | 240.16 | 1191.99 |
|  | *Danaus plexippus* | 14708 | 6221.09 | 1195.98 | 5.33 | 224.37 | 1160.42 |
| **Final** | -- | 15375 | 8906.70 | 1383.46 | 6.39 | 216.54 | 1396.03 |

**Table S11. The statistics of gene function annotation in *Papilio bianor* genome.**

| **Database** | **Number of annotated genes** | **Percent to all genes (%)** |
| --- | --- | --- |
| InterPro | 10,171 | 66.15 |
| GO | 7,532 | 48.99 |
| KEGG | 8,480 | 55.15 |
| Swissprot | 9,868 | 64.19 |
| TrEMBL | 11,381 | 74.02 |
| NR | 10,072 | 65.51 |
| Cog | 4,871 | 31.68 |
| Annotated | 13,343 | 86.78 |
| Unanotated | 2,032 | 13.22 |
| **Total** | 15,375 | -- |

**Table S12. The GO term enrichment of expanded gene families in *Papilio bianor* genome.**

| **GO ID** | **GO Term** | **GO Class** | **Adjusted Pvalue** | **Gene number** |
| --- | --- | --- | --- | --- |
| GO:0000786 | nucleosome | CC | 3.84E-02 | 13 |
| GO:0003777 | microtubule motor activity | MF | 7.60E-03 | 13 |
| GO:0004553 | hydrolase activity, hydrolyzing O-glycosyl compounds | MF | 5.08E-05 | 23 |
| GO:0005213 | structural constituent of chorion | MF | 0 | 44 |
| GO:0005506 | iron ion binding | MF | 0 | 65 |
| GO:0005576 | extracellular region | CC | 1.98E-03 | 27 |
| GO:0006334 | nucleosome assembly | BP | 4.44E-02 | 9 |
| GO:0007018 | microtubule-based movement | BP | 7.60E-03 | 13 |
| GO:0007275 | multicellular organism development | BP | 0 | 44 |
| GO:0007304 | chorion-containing eggshell formation | BP | 0 | 44 |
| GO:0007601 | visual perception | BP | 4.06E-02 | 6 |
| GO:0007602 | phototransduction | BP | 4.06E-02 | 6 |
| GO:0016705 | oxidoreductase activity, acting on paired donors, with incorporation or reduction of molecular oxygen | MF | 0 | 65 |
| GO:0020037 | heme binding | MF | 0 | 65 |
| GO:0030286 | dynein complex | CC | 9.66E-09 | 13 |
| GO:0042600 | chorion | CC | 0 | 44 |
| GO:0055114 | oxidation-reduction process | BP | 2.16E-09 | 87 |

**Table S13. The GO term enrichment of contracted gene families in *Papilio bianor* genome.**

| **GO ID** | **GO Term** | **GO Class** | **Adjusted Pvalue** | **Gene number** |
| --- | --- | --- | --- | --- |
| GO:0003924 | GTPase activity | MF | 3.58E-07 | 12 |
| GO:0005200 | structural constituent of cytoskeleton | MF | 0 | 12 |
| GO:0005874 | microtubule | CC | 0 | 12 |
| GO:0007017 | microtubule-based process | BP | 3.05E-13 | 12 |
| GO:0009055 | electron carrier activity | MF | 9.06E-06 | 9 |
| GO:0016491 | oxidoreductase activity | MF | 1.35E-05 | 23 |
| GO:0016614 | oxidoreductase activity, acting on CH-OH group of donors | MF | 0 | 20 |
| GO:0016758 | transferase activity, transferring hexosyl groups | MF | 4.91E-04 | 9 |
| GO:0022857 | transmembrane transporter activity | MF | 4.42E-03 | 13 |
| GO:0045211 | postsynaptic membrane | CC | 1.07E-02 | 4 |
| GO:0050660 | flavin adenine dinucleotide binding | MF | 7.63E-13 | 20 |
| GO:0051536 | iron-sulfur cluster binding | MF | 3.02E-05 | 9 |
| GO:0055085 | transmembrane transport | BP | 3.72E-02 | 23 |
| GO:0055114 | oxidation-reduction process | BP | 2.78E-02 | 29 |
