# Supplementary figures and images for: Chromosomal-level reference genome of Chinese peacock butterfly (Papilio bianor) based on third-generation DNA sequencing and Hi-C analysis
Source: Gigascience. 2019 Nov 4;8(11):giz128. doi: 10.1093/gigascience/giz128 (PMC6827417; doi:10.1093/gigascience/giz128)

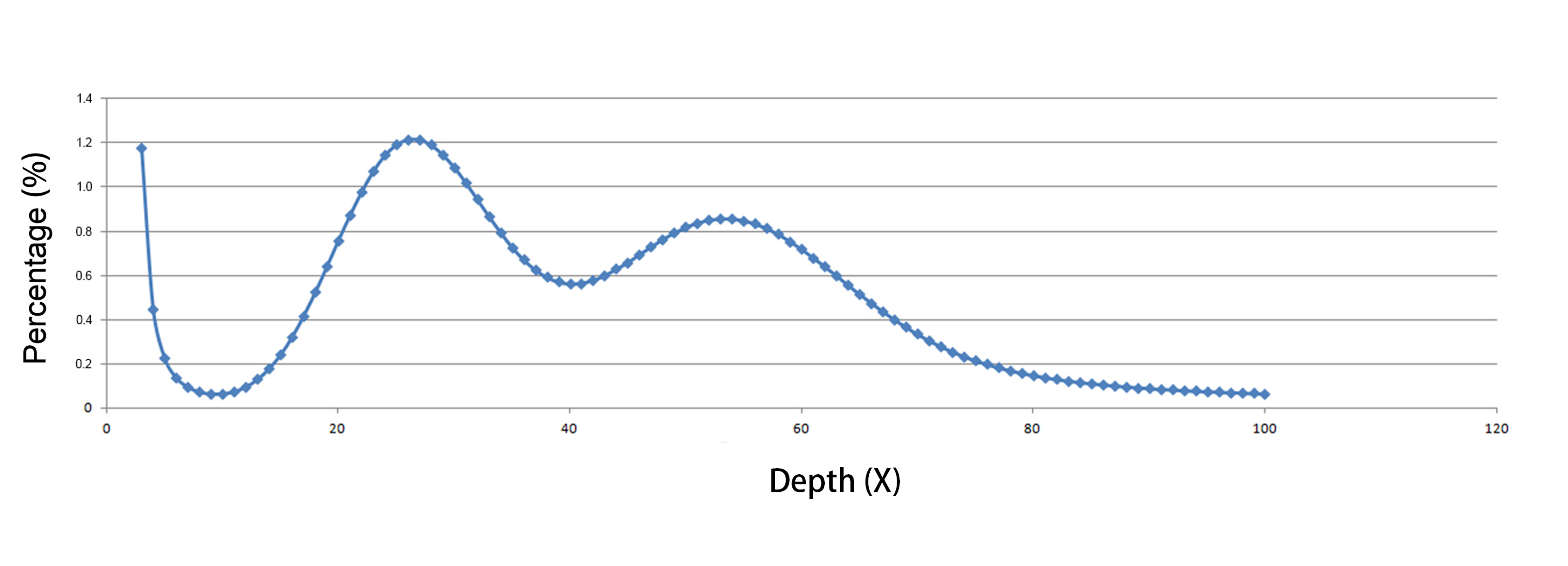

Supplement: giz128_Supplemental_Files [file giz128_supplemental_files.zip › supplement data/GIGA-D-19-00120_Figure S1.jpg]

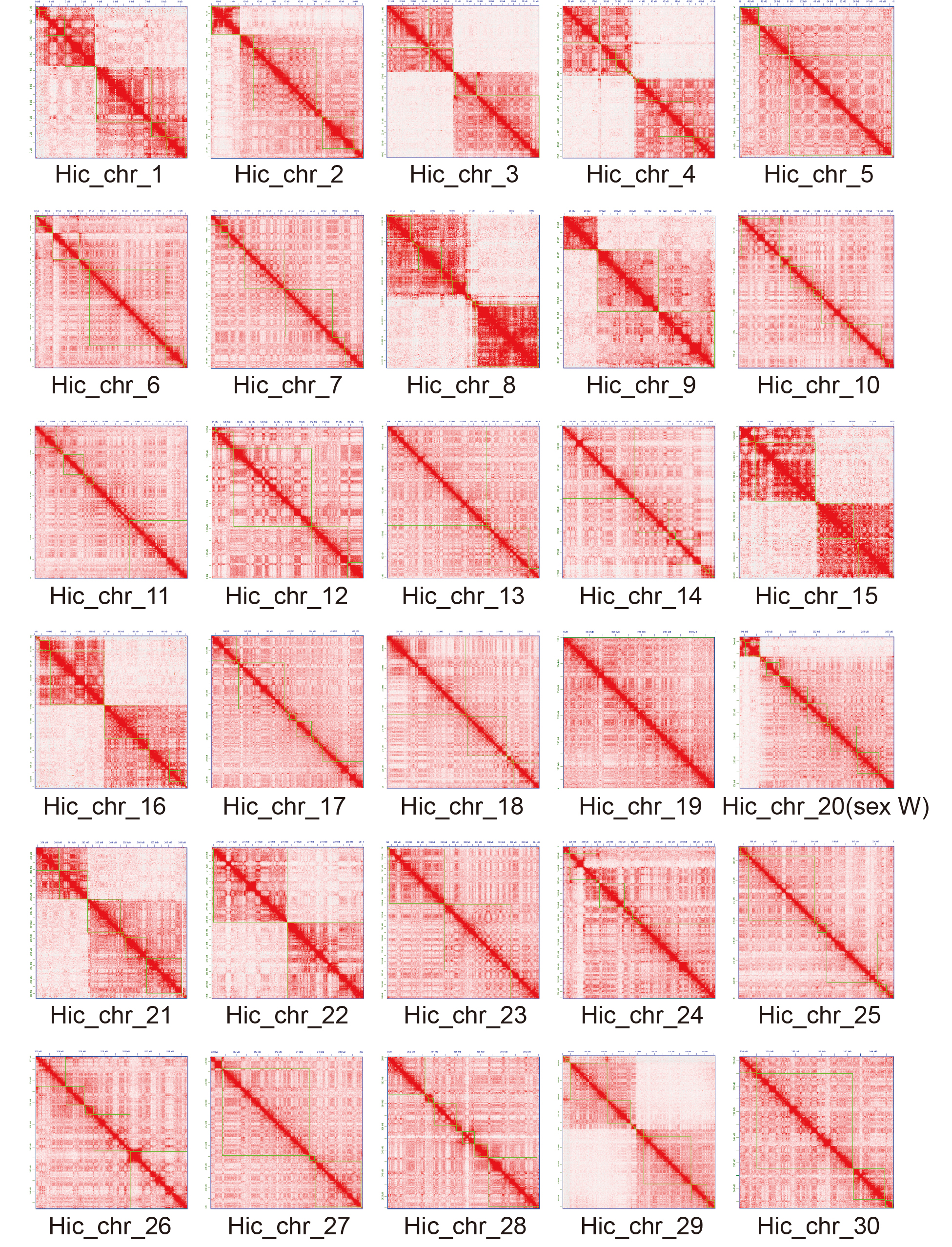

Supplement: giz128_Supplemental_Files [file giz128_supplemental_files.zip › supplement data/GIGA-D-19-00120_Figure S2.jpg]

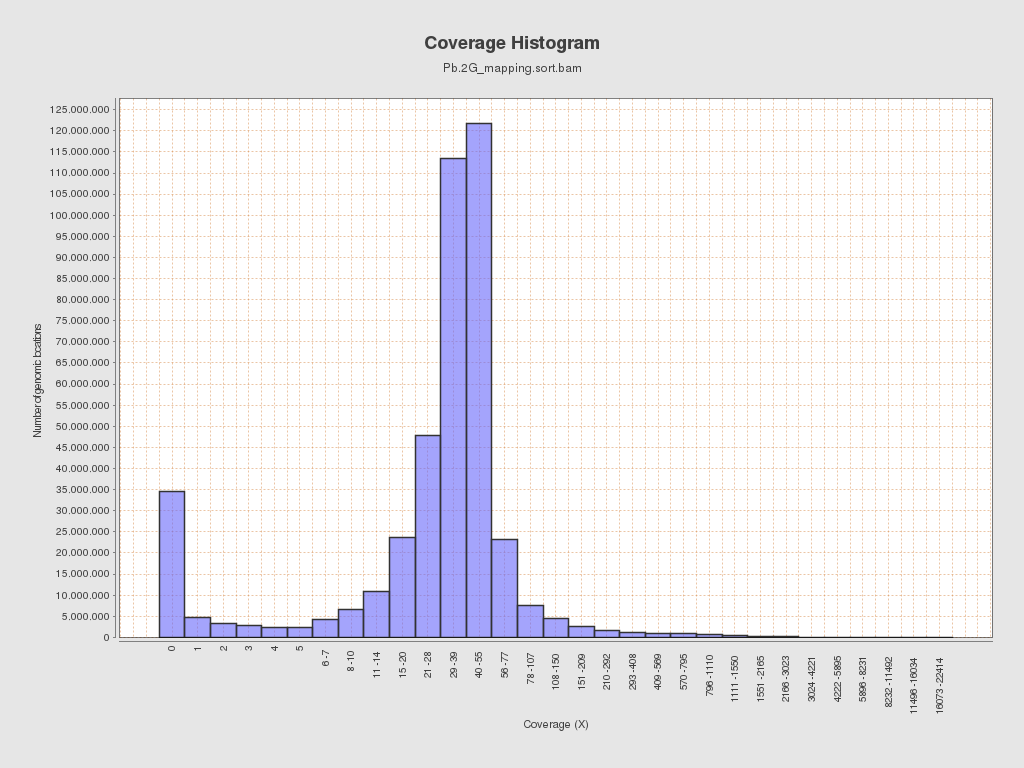

Supplement: giz128_Supplemental_Files [file giz128_supplemental_files.zip › supplement data/GIGA-D-19-00120_Figure S3.png]

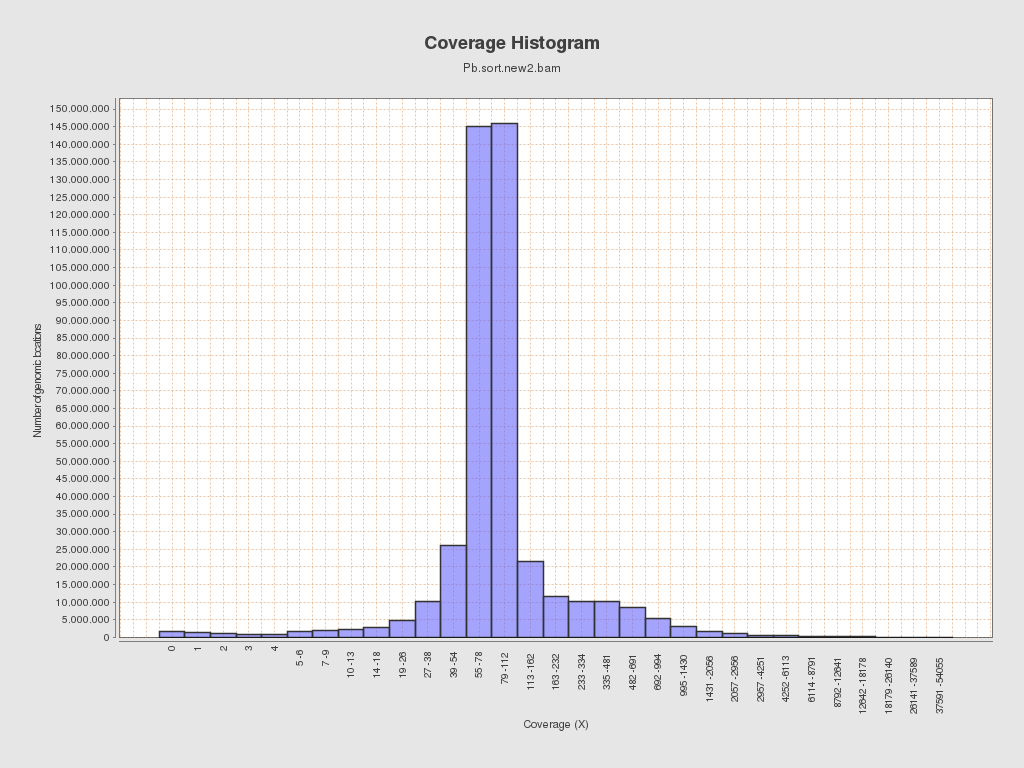

Supplement: giz128_Supplemental_Files [file giz128_supplemental_files.zip › supplement data/GIGA-D-19-00120_Figure S4.png]

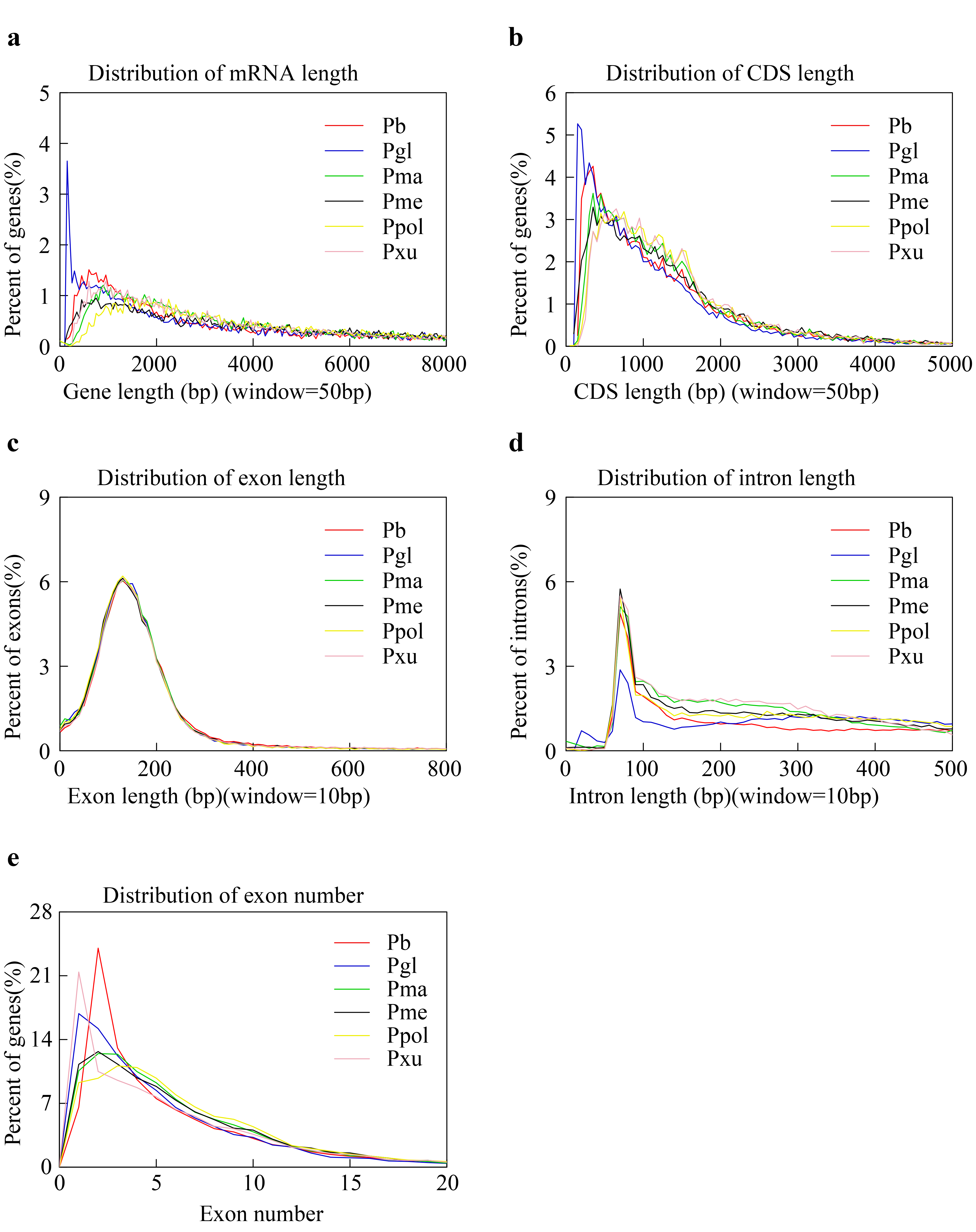

Supplement: giz128_Supplemental_Files [file giz128_supplemental_files.zip › supplement data/GIGA-D-19-00120_Figure S5.jpg]

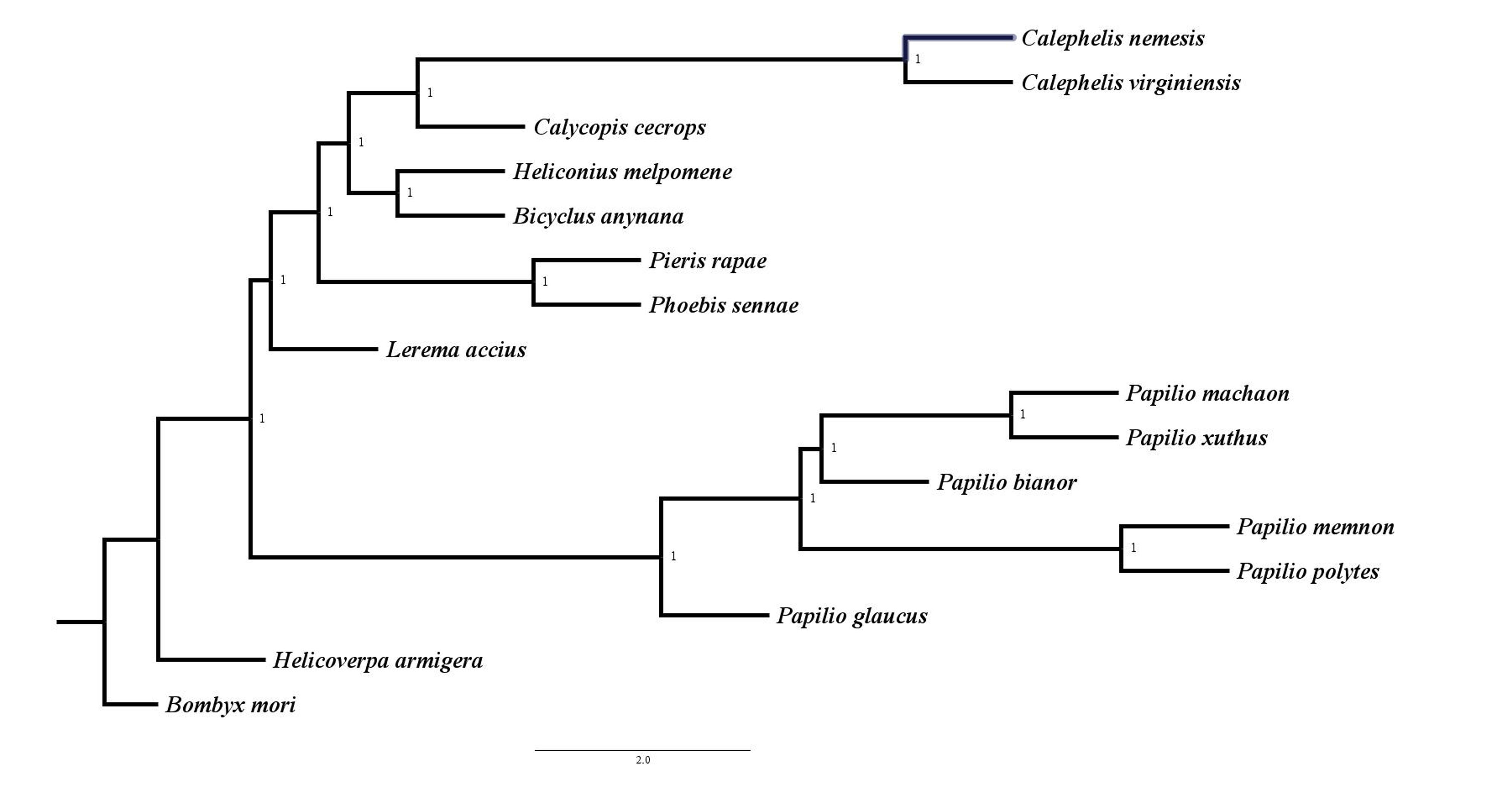

Supplement: giz128_Supplemental_Files [file giz128_supplemental_files.zip › supplement data/GIGA-D-19-00120_Figure S6.jpg]
