# Supplementary material for: Chromosomal-level reference genome of Chinese peacock butterfly (Papilio bianor) based on third-generation DNA sequencing and Hi-C analysis
Source: Gigascience. 2019 Nov 4;8(11):giz128. doi: 10.1093/gigascience/giz128 (PMC6827417; doi:10.1093/gigascience/giz128)
Supplement: giz128_Response_to_Reviewer_Comments_Original_Submission [file giz128_response_to_reviewer_comments_original_submission.pdf]

Author's Response To Reviewer Comments

Close

Dear Prof. Zhou,

We are submitting our revised manuscript entitled “Chromosomal-level reference genome of Chinese peacock butterfly (Papilio bianor) based on third-generation DNA sequencing and Hi-C analysis” (Manuscript ID: GIGA-D-19-00120). We appreciate all the positive comments and valuable suggestions from you and reviewers.

Based on reviewers’ comments and editor’s instructions, we have endeavored to improve our manuscript mainly as follows: (1) we have performed reanalysis or extension of the methods used for genome assembly and annotation, evolutionary analyses including syntenic relationships between gemomes of P. bianor and P. xuthus, phylogenetic relationships of Papilionoidea and demographic history of P. bianor as reviewer#1 suggested and added methodological details in revised MS; (2) we have confirmed the high repeat content in P. bianor genome using the methods in the reference recommended by reviewer#2; (3) we have checked the whole manuscript carefully, and improved the language and writing as reviewer#2 suggested; (4) we have added the RRID (Research Resource Identification Initiative ID) number in our revised manuscript as editor suggested. We also carefully checked the entire manuscript and corrected any errors. All the changes were highlighted in yellow.

We provide detailed point-by-point responses to the reviewers’ comments as follows.

Thank you very much for your consideration.

With best regards,
Sincerely yours

Xueyan Li, Ph.D
State Key Laboratory of Genetic Resources and Evolution
Kunming Institute of Zoology, Chinese Academy of Sciences (CAS), Kunming, Yunnan 650223, China
Email: lixy@mail.kiz.ac.cn, Tel: 86-871-68125339, Fax: 86-871-68125338

Wen Wang, Ph.D
State Key Laboratory of Genetic Resources and Evolution
Kunming Institute of Zoology, Chinese Academy of Sciences (CAS), Kunming, Yunnan 650223, China
Center for Ecological and Environmental Sciences, Northwestern Polytechnical University, Xi’an 710072, China
Email: wwang@mail.kiz.ac.cn

Point-by-Point Responses

Revierer #1: Dear Dr. Hongling Zhou,
Sihan Lu, Jie Yang, Xuelei Dai, Feiang Xie, et al assembled a high-quality reference genome of the Chinese Peacock Swallowtail, Papilio bianor. It was then used to carry out phylogenetic and demographic inference along with molecular dating.

In my opinion this paper is suitable for publication in GigaScience and will be of broad interest as: 1) Chromosome level assembly of a heterozygous insect genome is carried out, 2) shows an interesting expansion of repetitive elements and gene families in the P. bianor lineage, and 3) provides a good resource for phylogenetics and comparative genomics within the Lepidoptera. The manuscript is well laid out, however, proper expression is lacking in many places and the manuscript would be improved greatly if copy-edited.
R: We thank the Reviewer for the positive comments on this work.

The methods used for the assembly, annotation and evolutionary analyses are suitable for the results presented, but are generally too vague to allow for one to repeat the analysis.

I have some concerns with the manuscript that should be addressed before the paper would be suitable for publication. Most concerns relate to the text itself but some may involve reanalysis or extension of the analyses performed. Please see attached specific comments.
R: We thank the reviewer for suggestive and valuable comments. As suggested, we performed reanalysis or extension of analyses including syntenic relationships between gemomes of P. bianor and P. xuthus, phylogenetic relationships of Papilionoidea and demographic history of P. bianor.

1) Abstract: "The final assembly is 402.00 Mb on 30 chromosomes (29 autosomes and 1 sex chromosomes W)".
As males were sequenced, only Z chromosome sequence is expected as female Lepidopterans are ZW.
R: Thank you for the comment. We have corrected it in the revised manuscript.

2) Line 159 - 162: Finally, we obtained the chromosomal-level high-quality assembly of P. bianor with total length of ~402.00 161 Mb and the longest scaffold N50 (12.51 M) among the published butterfly genomes (Table 1 & Table S4).
It is unclear to me where the value of 402Mb comes from. Table S3 reports 381.5Mb and 421.5Mb as the chromosome-assigned and total scaffold length respectively. Table S4 reports 421,524,737bp with an N50 of 13,120,256 whereas Table 1 reports a genome size of 402 Mb with an N50 of 12813kb. Was ~20Mb of the assembly removed during the reduction of heterozygous contigs?
R: Thank you for these comments. In previous MS, we made unit conversion of genome size based on the unit conversion of storage capacity (1 Tb=1024 Gb, 1 Gb=1024 Mb, 1 Mb=1024 Kb, 1 Kb=1024 bp), resulting in 421,524,737 bp to 402 Mb (421,524,737 bp/1024 ≈ 411,645 Kb, 411,645 Kb/1024 ≈ 402 Mb), and N50 of 13,120,256 bp to 12813 Kb. After careful checking, we agree decimal system (i.e. 1 kb = 1000 bp, 1 Mb = 1000 kb) should be adopted in measuring genome size. Thus, we have corrected our description in the revised MS.

3) Line 174 – 176: Thirdly, we compared syntenic relationships between gemomes of P. bianor and P. xuthus (Fig. 1c) and found that 94.96% of the P. bianor assembled genome sequences can be aligned (1:1) to the P. xuthus reference genome.
As P. bianor has a genome ~2x the size of P. xuthus ~95% cannot be aligned 1:1.
R: Thank you for the valuable comment. It was our mistake to generate the illogical ratio in the previous MS, because we included the whole length of those chromosomes in case they can be aligned 1:1 with high-confidence (-m 0.01) (i.e. (aligned regions + unaligned regions) of a chromosome). Here, we performed the reanalysis as the followings: we aligned P. bianor base-pair to at most one P. xuthus base-pair, and then swapped the sequences and ran last-split with high-confidence (-m 0.01) to get 1-to-1 alignments (that is to say, only aligned regions were included). Finally, we found that 61,082,412 bp of the P. bianor assembled genome sequences can be aligned (1:1) to the P. xuthus reference genome. We have modified it to a more appropriate way. We have made the following changes in the text accordingly: "Thirdly, we compared syntenic relationships of P. bianor genome with that of P. xuthus, which is the only chromosomal-level assembly (by linkage map methods) [9] among all Papilio reference genomes and thus was considered to the best-assembled one (Fig. 1c). We found that 61,082,412 bp of the P. bianor assembled genome sequences can be aligned (1:1) with high-confidence (-m 0.01) to the P. xuthus reference genome." (Page 9, Line 183-188)

4) Line 251: 1378 one to one single orthologs
It is not clear how these were identified, were they from the BUSCO analysis?
R: Thank you for the comment. We have added related methods in our revised manuscript to make it more explicit: "1378 one-to-one single copy orthologs which contain only one protein for each species were collected and clustered by OrthoMCL (version 2.0.9; OrthoMCL DB: Ortholog Groups of Protein Sequences, RRID:SCR_007839) [83] from these 16 species and their nucleic acid sequences were aligned using PRANK (version 3.8.31; PRANK, RRID:SCR_017228) [85]." (Page 12-13, Line 276-280)

5) Line 253 – 254: construct the phylogenetic trees using RAxML (version 7.2.8; RAxML, RRID:SCR 006086) [83] by choosing the GTR+G+I model.
Was a concatenated alignment of all the genes used or were partitions set? If no partitions were set it would be worth setting gene partitions or using PartitionFinder to determine partitions. Alternatively, the authors should consider constructing gene trees for each of the single copy orthologs and inferring the species tree from these using an ILS aware algorithm such as ASTRAL (https://github.com/smirarab/ASTRAL). This would remove the need for partitioning and reanalysis of

the entire supermatrix using RAxML.
R: Thank you for valuable suggestion. We have added the following sentences to make this part more readable: "Furthermore, in order to make our result more clarified, we also have constructed the gene trees for each of the orthologs with RAxML software (version 7.2.8; RAxML, RRID:SCR_006086) [84] by choosing the GTR+G+I model and inferred the species tree from these with ASTRAL software (version 5.6.3) [87] (Fig. S4). As expected, the results are consistent with each other." (Page 13, Line 282-287).

We also give some explanations here.
As shown in Figure 2c, a concatenated alignment of all the genes (i.e. 1378 one to one single orthologs previously identified) was used for constructing the phylogenetic trees with RAxML software (version 7.2.8; RAxML, RRID:SCR_006086) [1] by choosing the GTR+G+I model (more details see discussion of Fig. 2c in our manuscript). Furthermore, in order to make our manuscript more clarified, we also have constructed the gene trees for each of the 1378 one to one single copy orthologs with RAxML software (version 7.2.8; RAxML, RRID:SCR_006086) [1] by choosing the GTR+G+I model and inferred the species tree from these with ASTRAL software (version 5.6.3) [2]. The related tree was added as Fig. S6. As expected, both results are consistent with each other.

Figure 2c. Maximum Likelihood (ML) phylogenetic tree of Papilionoidea constructed by the concatenated alignment of 1378 one to one single copy orthologue genes. The numbers in the square brackets on the nodes are the 95% confidence intervals of divergence time. The red dots are fossil evidence downloaded from the TIMETREE website and the black dots are inferred time obtained from the TIMETREE website. Both of them were used to calibrate divergent time.

Figure S6. Maximum Likelihood (ML) phylogenetic tree of Papilionoidea constructed by the merging each of the single copy orthologs.

6) Line 254 – 256: The phylogeny was further analyzed by PAML MCMCtree (version 4.5; PAML, RRID:SCR 014932) program [84], and calibrated with published timings for the divergence of difference species [85].
This is insufficient information to reproduce the analysis. Include clock type, substitution model, and if default parameters were used for alpha rate, number of categories, and other priors. Also report how many chains were run and if the model reached convergence.
Were all internal nodes informed using TimeTree? This may cause over parameterization, i.e. Do calibration times match well with the estimated split times of your nodes?
It would also be good to include the dates used as TimeTree ranges for calibration nodes may be updated in the future.
R: Thank you for the insightful comments. We have added the following sentence accordingly: "To further investigate the divergence time of these species, the phylogeny was further analyzed by MCMCtree in PAML (version 4.5; PAML, RRID:SCR_014932) software [88] with default parameters, and calibrated with published divergent times of some nodes estimated from fossil evidence or obtained from TIMETREE website [89]." (Page 13, Line 287-291). We also marked the nodes for calibrating the time in the revised figure (Fig. 2c), and improved the legend of Fig. 2c accordingly (Line 636-641).

We give some explanations here.
All related parameters were adopted the default parameters, including the clock type (correlated rates), substitution model (HKY85), alpha rate (0.5), number of categories, and other priors. 100,500,000 (burnin=500,000, sampfreq=5,000, nsample=20,000) chains were run and the model reached coveragence in the analysis.
The red dots are fossil evidence downloaded from the TIMETREE website and the black dots are inferred time obtained from the TIMETREE website. Both of them were used to calibrate the results (as shown in Fig. 2c & Fig. R1). The fossil evidences reveal that Bombyx mori diverged from the common ancestor of Helocoverpa armigera and Papilionoidea 52-177 million years ago (mya) while the split of Papilionoidea was 76-146 mya. The inferred times obtained from the TIMETREE website show that the divergence times between Bicyclus anynana and Heliconius melpomene, between Pieris rapae and Phoebis sennae, between Papilio polytes and Papilio memnon, between Papilio xuthus and Papilio machaon are 68-100 mya, 50-82 mya, 9.7-15.1 mya, and 17.6-33.6 mya, respectively. The inferred times also indicate that Papilio glaucus diverged with the common ancestor of other species in Papilio 18-61 million years ago while Pieridae diverged from the common ancestor of Nymphalidae, Lycaenidae, and Riodinidae 79-118 mya.
The results show that the estimated split times of these two results (inferred using orthologous genes and inferred using four-fold degenerate sites) are similar, which are consistent with the timetree results.

Figure 2c. Maximum Likelihood (ML) phylogenetic tree of Papilionoidea constructed by the concatenated alignment of 1378 one to one single copy orthologue genes. The numbers in the square brackets on the nodes are the 95% confidence intervals of divergence time. The red dots are fossil evidence downloaded from the TIMETREE website and the black dots are inferred time obtained from the TIMETREE website. Both of them were used to calibrate divergent time.

Figure R1. Maximum Likelihood (ML) phylogenetic tree of Papilionoidea constructed by the concatenated alignment of four-fold degenerate sites (4dTV). The numbers in the square brackets on the nodes are the 95% confidence intervals of divergence time. The red dots are fossil evidence downloaded from the TIMETREE website and the black dots are inferred time obtained from the TIMETREE website. Both of them were used to calibrate the results.

7) Line 258 – 259: all Papilio species is a monophyly, and diverged from other butterflies approximately 41.07-56.86 mya
Consider rephrasing to: "all species of Papilio analysed are in monophyly with a crown node age of approximately 41.07-56.86 mya"
R: Thanks for Reviewer's suggestion. We improved as suggested. (Page 13, Line 293/4)

8) Line 264 – 267: We also inferred demographic histories of P. bianor by SNP calling of Illumina short reads against assembled genome using the Pairwise Sequentially Markovian Coalescence (PSMC) analysis [89] (0.1 × 10-8 mutations per site per generation calculated by r8s [90]; three or four generations per year [47]).
Specify what tools were used for variant calling, was SAMtools mpileup used (as recommended on Heng Li's github https://github.com/lh3/psmc)?
Please also specify time intervals used and I recommend bootstrapping the analysis, a detailed example of how to do this can be found on Heng Li's github page for PSMC. Using different time intervals may also increase resolution in the recent history.
R: Thank you for the valuable suggestions. As suggested, we improve this part as the followings: "We also inferred the demographic histories of P. bianor applying the Pairwise Sequentially Markovian Coalescence (PSMC; PSMC, RRID:SCR_017229; with -p 64*1 parameters) analysis [93] (3.56 × 10-3 mutations per site per generation calculated by r8s [94]; three or four generations per year [48]), which was carried out by mapping Illumina short reads to the assembled genome with BWA (version 0.7.12-r1039; BWA, RRID:SCR_010910) [58] and calling variants with SAMtools (version 1.3.1; SAMTOOLS, RRID:SCR_002105; with samtools mepileup -C50 –uf parameters) [95]." (Page 13-14, Line 299-306).

Here, we also provide some explanation.
We used the BWA-mem to map short reads to the reference genome, and then called variants to get the bcf format file using samtools mepileup (samtools mepileup -C50 -uf). Bcftools (bcftools call –c) was used to convert the bcf format into the vcf format for the further PSMC analysis.
We have specified the time intervals and have changed the time intervals from "-p 28*2+3+5" to "-p 64*1" to increase resolution in the recent history (as shown in Fig. 2d). We also have run 100 bootstrap replicates using PSMC software to test the robust variations (Fig. 2d).

Figure 2d. The dynamic changes of the effective population size are plotted using PSMC software, with 100 bootstrap replicates to test the robust variations. The parameter "g" represents the generation time in years, and the parameter "μ" means the per generation mutation rate.

Since the species is three or four generations a year, we have chosen two extreme values (g=0.25, Fig. R2; g=0.4, Fig. R3) to ensure the availability of information.

Figure R2. Demographic history of P. bianor are plotted using PSMC software. The parameter "g=0.25" indicates generation time in years, and "μ=0.89*10-9" indicates genomic substitution rate.

Figure R3. Demographic history of P. bianor are plotted using PSMC software. The parameter "g=0.4" indicates generation time in years, and "μ=0.14*10-8" indicates genomic substitution rate.

Based on the above results, we have reedited Fig. 2d accordingly. We also have changed the wrong date "0.1 × 10-8 mutations" to the right one "3.56 × 10-3 mutations" in the revised manuscript because we confused the result calculated by r8s with parameter "μ" in our previous manuscript.

9) Line 268 -270: Our result suggested that the effective population size increased significantly from the last interglacial period (LIG, approximately 0.1 million years before present) to its maximum at the last glacial maximum (LGM, approximately 0.01 million years before present).
Does this match the demographic history of any other Papilio species?
R: Thank you for this important comment. Our result on the trend of the demographic history of P. bianor is in good agreement with those of other five Papilio species (P. ambrax, P. phestus, P. polytes alphenor, P. protenor, and P. polytes polytes) reported previously [3] (Fig. R4). We have changed the sentence and cited the corresponding reference accordingly as the followings: "Our result suggested that the effective population size increased significantly corresponded to the transition phase from the last interglacial period (LIG, approximately 0.14-0.12 million years before present) to the last glacial maximum (LGM, approximately 0.021-0.018 million years before present) (Fig. 2d), which is in good agreement with other five Papilio species [96]." (Page 14, Line 306-310)

Figure R4. Historical effective population sizes were inferred from eight individual genomes using PSMC, assuming a mutation rate of $\mu=3 \times 10-9$ and an average generation time of g=0.25 year. (quoted from: Zhang W et al (2017) doi: Artn 1269 10.1038/S41467-017-01370-1)

10) Other comments:
Heterozygosity is relatively high (1.8%) but samples were collected from the wild. Many genome studies use inbred strains to reduce polymorphism. Could there be any bias in these statistics?
Data source (references) for Table 1.
R: Thanks you for the insightful comments. Yes, as Reviewer pointed out, wild animals (especially insect) usually exhibit high heterozygosity. To cultivate inbred strains is a reliable method to reduce polymorphism, which, however, is not feasible in all cases, especially for wild insects. Both above mentioned points are just the bottlenecks to impede the dissection of reference genomes from more insects, especially only using Next-generation sequencing. PacBio SMRT sequencing technology break the bottlenecks for dissecting reference genomes with high heterozygosity. In this work, heterozygosity was calculated based on k-mer distribution analysis, which is a commonly used method to estimate heterozygosity of genomes (e.g. P. machaon and P. xuthus in butterflies [4]). As suggested, to avoid confusion, we have removed the percentage heterozygosity in the Abstract. We also have provided the algorithm for different species in the interpretation of Table 1: "The heterozygosity of P. bianor, P. machaon and P. xuthus were calculated based on k-mer distribution analysis. The heterozygosity values of others (P. glaucus, A. lyciades, L. accius, M. ursus violae, P. rapae, P. sennae, D. plexippus, C. nemesis, C. virginiensis, C. cecrops) were estimated using the Genome Analysis Toolkit (GATK)." (Line 619-621)
Reviewer #2: Review of Lu et al submission: Chromosomal-level reference genome of Chinese peacock butterfly (Papilio bianor) based on third-generation DNA sequencing and Hi-C analysis

Lu et al describe the construction of a chromosomal-level reference genome assembly for Papilio bianor and set this in the context of other butterfly genome assemblies. This manuscript was a pleasure to review. The genome assembly project has been carefully conducted and a good level of supporting data is included. The manuscript requires some editing for language (almost all of my minor comments address language issues) but the intentions and the analyses that have been carried out are nevertheless clear and this assembly will be an important resource for the butterfly research community and beyond.
R: We thank reviewer for positive comments on our work. We also appreciate Reviewer for careful and kind checking on our MS and all valuable comments. As suggested, we improved this MS.

Major comments
Regarding repeat content, to what extent does your observation of such extremely high repeat content reflect your methods of ascertainment and methods used in other studies? For example, see Platt et al (2016) doi: 10.1093/gbe/evw009. How confident are you that this repeat expansion is genuine and not (partly or wholly) an artefact of ascertainment? Whilst this expansion sits neatly with the increased genome size, I would like to see some discussion of the limitations of making these broad comparisons between assemblies generated and analysed with different methodologies.
R: Thanks you for the valuable comments. As suggested, we used the method in the recommended reference to annotate the repetitive sequences of P. bianor genome. And the related repetitive sequences content is consistent with the previously annotated. We have added the following sentence accordingly: "To confirm the reliability of high repetitive sequences in P. bianor, which is much higher than those (<35%) of other butterflies (Table 1), we also used other de novo annotation method reported by Platt II et al. and Lavoie et al [69, 70] to annotate the repetitive sequences of P. bianor

genome. Based on this method, P. bianor genome possesses 53% repeat elements, similar to the previous annotated results (Table 1 & Table S8), thus confirming high repetitive sequences in P. bianor genome." (Page 10, Line 214-220).

Here, we also provide some explanation.
In this research, the repeat content we calculated was up to 55%, in which 51% was from the de novo repeatmasker (Table S8). The library of de novo repeatmasker was produced by RepeatModeler software (version 1.0.4; RepeatModeler, RRID:SCR_015027) [5]. According to the reviewer's advice, we have referred to the method reported by Lavoie et al (2013) doi: 10.1186/1759-8753-4-21 [6], which was also recommended by Platt et al (2016) doi: 10.1093/gbe/evw009 [7]. In details, firstly, we blasted (-evalue 1e-5) the RepeatModeler outfile to the whole genome and then extended the resulting sequence by flanking 1000 bp to get the potential repeat seed sequence. Secondly, we used MUSCLE (version 3.8.31; MUSCLE, RRID:SCR_011812) [8] to align the extracted sequence, TRIMAL (version 1.2rev59; with -gt 0.6 -cons 60) [9] and EMBOSS (version 6.6.0.0; EMBOSS, RRID:SCR_008493; with cons -identity 3) [10] to generate the 50% majority rule consensus sequence. Finally, the consensus sequence was used as the new library to the repeatmasker program. In the modified method, we got 53% repeat elements, of which 48% was annotated from the consensus library. So the results obtained were not significantly different from the previous results. In addition, the result of k-mer evaluation of repeat content was 58.3%, which confirmed the high repeat ratio in this species.

In a similar vein, it would also be interesting to see some discussion of how data from different wild-caught individuals was combined to generate the assembly draft. Especially in the context of such high heterozygosity this has the potential to cause difficulties in assembly and might inflate estimates of genome size. This was alluded to in 141-163, when criteria for merging contigs were set out. I think further discussion of whether this had an impact on the assembly process would be of general interest to the GigaScience readership.
R: Thank you for the important comment. Yes, this is a topic of general interest. The amount of DNA extracted from single individual of especially many insects and even other invertebrates is usually not enough to satisfy such different types of sequencing as genome survey (Illumina), PacBio, and Hi-C, which, if possibly, is the best ideal case. We were also asked for our experience on this topic. Following our experience, it is acceptable to use different wild individuals. In our current study, one male adult was sued for Illumina sequencing, another male adult for PacBio sequencing, and a sample mixed from whole body of two male larval individuals (the fifth instar) was used for Hi-C sequencing. The results on quality evaluation of our assembled genomes (original lines 166-178; Revised lines 172-191) provided support to such a method that different individuals were used for different sequencing for de novo genome assembling. As suggested by reviewer, aiming to make this topic more explicit, we also added the coverage distribution of Illumina reads/PacBio reads mapping to the assembled genomes (Fig. S3, Fig. S4), which show a near normal distribution and thus indicate few heterozygous regions in assembled genome. Further, we improved the expression "All these results suggest that the assembled P. bianor genome is of high quality (including completeness, base level contiguity and accuracy) (Table 1)." to "All these results suggest that P. bianor genome, which is assembled based on PacBio reads, Illumina reads and Hi-C data sequenced from different wild individuals, is of high quality (including completeness, base level contiguity and accuracy) (Table 1)". (Page 9, Line 188-191)

Figure S3. The coverage distribution of Illumina reads mapping to Papilio bianor genome. The histogram follows a normal distribution, indicating few heterozygous regions in assembled genome.

Figure S4. The coverage distribution of PacBio reads mapping to Papilio bianor genome. The histogram follows a normal distribution, indicating few heterozygous regions in assembled genome.

It would be interesting to know why the authors selected P. xuthus for comparisons. Is there a particular biological justification? Table 1 suggests it may be considered the best-assembled of the other Papilio genomes. Perhaps include a statement in the text explaining why this pairwise comparison was selected?
R: Thanks you for the valuable suggestion. As suggested, we improved the description in the revised manuscript as the followings: "Thirdly, we compared syntenic relationships of P. bianor genome with that of P. xuthus, which is the only chromosomal-level assembly (by linkage map methods) [9] among all Papilio reference genomes and thus was considered to the best-assembled one (Fig. 1c). We found that 61,082,412 bp of the P. bianor assembled genome can be aligned (1:1) with high-confidence (-m 0.01) to the P. xuthus reference genome." (Page 9, Line 183-188)

Fig1b is an excellent overview but the detail of the heatmap is hard to see on this scale. Perhaps consider releasing per-chromosome plots as part of supplementary material.
R: Thank you. We have added the per-chromosome plots as Fig. S2 in the supplementary material. (Line 660/1)

Figure S2. Heatmap of per-chromosomal interactions. Each scaffold is framed with green block.

In general, I dislike the statements of priority (e.g. in line 103- this is the 4th chromosomal-level reference genome) as these are prone to become quickly out of date and also are somewhat irrelevant.
R: Thank you. As suggested, we have removed the information about priority in our manuscript. (Page 6, Line 105/6)

Minor comments:
1) 26: change "i.e. Chinese peacock" to "commonly known as the Chinese peacock butterfly"
R: Thank you. We modified it as suggested. (Page 2, Line 27/8)

2) 28: southeast
R: Thank you. We corrected it as suggested. (Page 2, Line 30)

3) 31: consider changing "is thus important" to "is an important foundation for"
R: Thank you. We improved it as suggested. (Page 2, Line 33)

4) 32: and the evolution of
R: Thank you. We corrected it as suggested. (Page 2, Line 34)

5) 33: highly
R: Thank you. We corrected it as suggested. (Page 2, Line 35)

6) 34: consider removing the percentage heterozygosity from the abstract as it needs further explanation.
R: Thank you. As suggested, we have removed the "percentage heterozygosity" from the abstract. (Revisions is made in Page 2, Line 36 in the revised manuscript)

7) 36/7: (29 autosomes and 1 W sex chromosome)
R: Thank you. We corrected it as suggested. (Page 2, Line 38)

8) 37: In total, 15,375…
R: Thank you. We improved it as suggested. (Page 2, Line 39)

9) 38: Phylogenetic analyses indicated that…
R: Thank you. We improved it as suggested. (Page 2, Line 40)

10) 43: What exactly do you mean by climate diversity? Climate change?
R: We thank the reviewer for bringing out this valuable point. Yes, what we want to expreess is that population of this species has expanded owing to the decrease of its natural enemies and its adaptation to climate change. We changed "climate diversity" to "climate change". (Page 2, Line 44)

11) 46: Our results lay the foundation for exploring the genetic basis…
R: Thank you. We corrected it as suggested. (Page 2, Line 47)

12) 54: I am not keen on this statement "Butterflies are one of most charming animals". I would prefer "Butterflies are widely considered….". In a similar vein, I am not convinced by the use of "inventor" to describe Bate's contribution to mimicry theory (line 61).
R: Thank you for the good suggestions. As suggested, we have changed "Butterflies are one of most charming animals" (line 55) to "Butterflies are widely considered as one of most charming animals"; we have changed the word "inventor" (line 62) to word "pioneer".

13) 58: change to "have been regarded as important model organisms in …"
R: Thank you. We corrected it as suggested. (Page 4, Line 59)

14) 63: I think this sentence needs a bit more development. Presumably the argument is that

technology development has rendered possible direct analysis (and even manipulation) of the genomes of individuals sampled from natural habitats without the need to inbreed to reduce heterozygosity or to develop lab lines etc.
R: Thank you for the suggestive comments. We have rewritten the following sentences accordingly: "With the feasibility to dissect the heterozygous genomes of such wild insects like butterflies and to perform genetic manipulation on them [9-11], butterflies have been becoming a promising system to explore the genetics, evolution, morphological diversification and speciation." Changed to: "With the technology development, it is possible to conduct direct analysis (and even manipulation) of the genomes of individuals sampled from natural habitats without the need of inbreeding to reduce heterozygosity or to develop lab lines [9-11], so butterflies have been becoming a promising system to explore the genetics, evolution, morphological diversification and speciation." (Page 4, Line 64-68)

15) 69: have had reference genomes assembled, at the time of writing. [care needed here as this is an active area- there are many others in the process of being assembled]
R: Thank you for the suggestive comments. We have added the statistical date (until May 1, 2019) in the revised manuscript. (Page 4, Line 72)

16) 71: Nymphalidae (or nymphalids)
R: Thank you for careful checking. We changed "nymphids" to "nymphalids". (Page 4, Line 73)

17) 73: linkage map methods
R: Thank you. We corrected it as suggested. (Page 4, Line 75)

18) 75: "...which may often result from …"
R: Thank you. We corrected it as suggested. (Page 4, Line 77)

19) 76-77 "will provide a unique opportunity to promote the evolutionary biological studies on the famous butterfly system." This sentence remains a bit unclear- what exactly do you mean by "famous butterfly system"?
R: Thank you for the suggestive comments. We changed "famous butterfly system" to "butterflies as an important model system". (Page 5, Line 79)

20) 80: "paved the way for the dissection of…"
R: Thank you. We improved it as suggested. (Page 5, Line 81/2)

21) 81: high-throughput
R: Thank you. We corrected it as suggested. (Page 5, Line 83)

22) 82: "which was developed to identify…. and is now also …."
R: Thank you. We corrected it as suggested. (Page 5, Line 84/5)

23) 87: has been reported
R: Thank you. We corrected is as suggested. (Page 5, Line 89-90)

24) 90: Known as the Chinese.. or the Chinese peacock,"
R: Thank you. We corrected is as suggested. (Page 5, Line 93)

25) 92: southeast
R: Thank you. We corrected is as suggested. (Page 5, Line 95)

26) 93: Its larvae mainly feed on plants of the Rutaceae family, such as … and it completes its life cycle in 40-50 days.
R: Thank you. We corrected is as suggested. (Page 5, Line 96-98)

27) 96: perhaps "structurally-coloured scales"?
R: Thank you. We corrected is as suggested. (Page 5, Line 99)

28) 99: prothoracicotropic
R: Thank you. We corrected is as suggested. (Page 5, Line 102)

29) 100: species delimitation
R: Thank you. We corrected is as suggested. (Page 6, Line 103)

30) 101: PacBio SMRT
R: Thank you. We corrected is as suggested.(Page 6, Line 104)

31) 134: A sample. Does the reference in 35 also detail DNA extraction method? If not please include details.
R: Thank you. We corrected "The sample" to "A sample". Yes, the changed reference in 36 details the DNA extraction method. (Page 7, Line 137)

32) 147: "…using Illumina reads as follows: "
R: Thank you. We corrected is as suggested. (Page 7, Line 150)

33) 154: as the previously reported
R: Thank you. We corrected is as suggested. (Page 8, Line 159)

34) 157: 3D de novo assembly [37] softwares to improve the assembly
R: Thank you. We corrected is as suggested. (Page 8, Line 162)

35) 158: change "quite possibly" to "likely"
R: Thank you. We corrected is as suggested. (Page 8, Line 164)

36) 168: Please specify which BUSCO database you are using to assess completeness
R: Thank you for the good suggestion. We used the insecta_odb9 database as the BUSCO database. We have added it in the revised manuscript. (Page 8, Line 174/5)

37) 178: suggest contiguity not continuity
R: Thank you for careful checking. We corrected is as suggested. (Page 9, Line 191)

38) 225: "also searched all predicted gene sequences against GenBank"
R: Thank you. We corrected is as suggested. (Page 11, Line 250)

39) 244: "to reveal the phylogenetic position"
R: Thank you. We have added the word "the" in the revised manuscript. (Page 12, Line 269)

40) 251:"one-to-one single copy orthologs"
R: Thank you. We corrected is as suggested. (Page 12, Line 276)

41) 252: Suggested edits: their nucleic acid sequences were aligned using PRANK (version 3.8.31) [82]. Gene alignments were concatenated and phylogenetic trees were constructed using RAxML (version 7.2.8; RAxML, RRID:SCR 254 006086) [83] with the GTR+G+I model. The phylogeny was further analyzed PAML MCMCtree (version 4.5; PAML, RRID:SCR 014932) program [84], and calibrated with published timings for the divergence of different species [85].
R: Thank you for the good suggestion. We corrected is as suggested. (Page 13, Line 279-291)

42) 258: Xuthus
R: Thank you. Here, "xuthus" is a species name (P. xuthus). In original MS, "P." and "xuthus" were separated in different line, which produced a confusement.

43) 258: suggest "The Papilio genus is monophyletic"
R: Thank you. We corrected is as suggested. (Page 13, Line 293/4)

44) 264: suggest "We also inferred the demographic history of P. bianor by mapping Illumina short reads to the assembled genome and applying PSMC analysis."
R: We do appreciate reviewer's professional indication. We have revised it accordingly: "We also inferred the demographic histories of P. bianor applying the Pairwise Sequentially Markovian Coalescence (PSMC; PSMC, RRID:SCR_017229; with -p 64*1 parameters) analysis [93] (3.56 × 10-3 mutations per site per generation calculated by r8s [94]; three or four generations per year [48]), which was carried out by mapping Illumina short reads to the assembled genome with BWA (version 0.7.12-r1039; BWA, RRID:SCR_010910) [58] and calling variants with SAMtools (version 1.3.1; SAMTOOLS, RRID:SCR_002105; with samtools mepileup -C50 –uf parameters) [95]." (Page 13-14, Line 299-306)

45) 279: "The P. bianor diverged.."

R: Thank you. We corrected it as suggested. (Page 14, Line 319)

46) 594: "Obtained from Chinese group"- please provide appropriate citation
R: Thank you. We added the citation ([9]). (Line 630)

47) 599: Consider changing "segments" to "functional classes" or equivalent, more informative term
R: Thank you. We changed "segments" to "functional classes" (Line 635)

48) 602: Note plot is based on PSMC in legend
R: Thank you for the suggestive comments. We redescribed the legend of (d) as follows: (d) The dynamic changes of the effective population size are plotted using PSMC software, with 100 bootstrap replicates to test the robust variations. The parameter "g" represents the generation time in years, and the parameter "μ" means the per generation mutation rate. (Line 641-645)

49) 631: Suggest changing to: The Hi-C data were filtered by HiC-Pro software. In total, 6,690,421 pairs of reads, corresponding 68.04% of the total Hi-C dataset, were used in downstream analyses.
R: Thank you. We improved it as suggested. (Line 687/8)

50) Table S4: I suspect "Contiguity" not continuity is the term you mean.
R: Thank you. We corrected it as suggested. (Line 690)

51) Table S5: please state which BUSCO library you are using for comparison
R: Thank you. We improved it as suggested. (Line 693)
References
1. Stamatakis A. RAxML version 8: a tool for phylogenetic analysis and post-analysis of large phylogenies. Bioinformatics. 2014;30 9:1312-3. doi:10.1093/bioinformatics/btu033.
2. Mirarab S, Reaz R, Bayzid MS, Zimmermann T, Swenson MS and Warnow T. ASTRAL: genome-scale coalescent-based species tree estimation. Bioinformatics. 2014;30 17:I541-I8. doi:10.1093/bioinformatics/btu462.
3. Zhang W, Westerman E, Nitzany E, Palmer S and Kronforst MR. Tracing the origin and evolution of supergene mimicry in butterflies. Nature communications. 2017;8 doi:10.1038/S41467-017-01370-1.
4. Li XY, Fan DD, Zhang W, Liu GC, Zhang L, Zhao L, et al. Outbred genome sequencing and CRISPR/Cas9 gene editing in butterflies. Nature communications. 2015;6 doi:10.1038/Ncomms9212.
5. Smith A, Hubley R and Green P. RepeatMasker Open-4.0.(2013-2015). 2016.
6. Lavoie CA, Platt RN, Novick PA, Counterman BA and Ray DA. Transposable element evolution in Heliconius suggests genome diversity within Lepidoptera. Mobile DNA. 2013;4 1:21.
7. Platt RN, Blanco-Berdugo L and Ray DA. Accurate transposable element annotation is vital when analyzing new genome assemblies. Genome biology and evolution. 2016;8 2:403-10.
8. Edgar RC. MUSCLE: multiple sequence alignment with high accuracy and high throughput. Nucleic acids research. 2004;32 5:1792-7. doi:10.1093/nar/gkh340.
9. Capella-Gutierrez S, Silla-Martinez JM and Gabaldon T. trimAl: a tool for automated alignment trimming in large-scale phylogenetic analyses. Bioinformatics. 2009;25 15:1972-3. doi:10.1093/bioinformatics/btp348.
10. Rice P, Longden I and Bleasby A. EMBOSS: The European molecular biology open software suite. Trends In Genetics. 2000;16 6:276-7. doi:10.1016/S0168-9525(00)02024-2.

Close
