# Supplementary material for: Chromosomal-level reference genome of Chinese peacock butterfly (Papilio bianor) based on third-generation DNA sequencing and Hi-C analysis
Source: Gigascience. 2019 Nov 4;8(11):giz128. doi: 10.1093/gigascience/giz128 (PMC6827417; doi:10.1093/gigascience/giz128)
Supplement: giz128_Reviewer_1_Report_Original_Submission — Christopher Ward -- 5/14/2019 Reviewed [file giz128_reviewer_1_report_original_submission.pdf]

Reviewer Report

Title: Chromosomal-level reference genome of Chinese peacock butterfly (Papilio bianor) based on third-generation DNA sequencing and Hi-C analysis

Version: Original Submission **Date:** 5/14/2019

Reviewer name: Christopher Ward

Reviewer Comments to Author:

Dear Dr. Hongling Zhou,
Sihan Lu, Jie Yang, Xuelei Dai, Feiang Xie, et al assembled a high-quality reference genome of the Chinese Peacock Swallowtail, Papilio bianor. It was then used to carry out phylogenetic and demographic inference along with molecular dating.
In my opinion this paper is suitable for publication in GigaScience and will be of broad interest as: 1) Chromosome level assembly of a heterozygous insect genome is carried out, 2) shows an interesting expansion of repetitive elements and gene families in the P. bianor lineage, and 3) provides a good resource for phylogenetics and comparative genomics within the Lepidoptera. The manuscript is well laid out, however, proper expression is lacking in many places and the manuscript would be improved greatly if copy-edited.
The methods used for the assembly, annotation and evolutionary analyses are suitable for the results presented, but are generally too vague to allow for one to repeat the analysis.
I have some concerns with the manuscript that should be addressed before the paper would be suitable for publication. Most concerns relate to the text itself but some may involve reanalysis or extension of the analyses performed. Please see attached specific comments.

Level of Interest

Please indicate how interesting you found the manuscript: Choose an item.

Quality of Written English

Please indicate the quality of language in the manuscript: Choose an item.

Declaration of Competing Interests

Please complete a declaration of competing interests, considering the following questions:

- Have you in the past five years received reimbursements, fees, funding, or salary from an organisation that may in any way gain or lose financially from the publication of this manuscript, either now or in the future?

- Do you hold any stocks or shares in an organisation that may in any way gain or lose financially from the publication of this manuscript, either now or in the future?
- Do you hold or are you currently applying for any patents relating to the content of the manuscript?
- Have you received reimbursements, fees, funding, or salary from an organization that holds or has applied for patents relating to the content of the manuscript?
- Do you have any other financial competing interests?
- Do you have any non-financial competing interests in relation to this paper?

If you can answer no to all of the above, write 'I declare that I have no competing interests' below. If your reply is yes to any, please give details below.

I declare that I have no competing interests

I agree to the open peer review policy of the journal. I understand that my name will be included on my report to the authors and, if the manuscript is accepted for publication, my named report including any attachments I upload will be posted on the website along with the authors' responses. I agree for my report to be made available under an Open Access Creative Commons CC-BY license (http://creativecommons.org/licenses/by/4.0/). I understand that any comments which I do not wish to be included in my named report can be included as confidential comments to the editors, which will not be published.

Choose an item.

To further support our reviewers, we have joined with Publons, where you can gain additional credit to further highlight your hard work (see: https://publons.com/journal/530/gigascience). On publication of this paper, your review will be automatically added to Publons, you can then choose whether or not to claim your Publons credit. I understand this statement.

Yes Choose an item.
