# Supplementary material for: Chromosomal-level reference genome of Chinese peacock butterfly (Papilio bianor) based on third-generation DNA sequencing and Hi-C analysis
Source: Gigascience. 2019 Nov 4;8(11):giz128. doi: 10.1093/gigascience/giz128 (PMC6827417; doi:10.1093/gigascience/giz128)
Supplement: giz128_Reviewer_2_Report_Original_Submission — Annabel Charlotte Whibley -- 5/19/2019 Reviewed [file giz128_reviewer_2_report_original_submission.pdf]

Reviewer Report

Title: Chromosomal-level reference genome of Chinese peacock butterfly (Papilio bianor) based on third-generation DNA sequencing and Hi-C analysis

Version: Original Submission **Date:** 5/19/2019

Reviewer name: Annabel Charlotte Whibley

Reviewer Comments to Author:

Review of Lu et al submission: Chromosomal-level reference genome of Chinese peacock butterfly (Papilio bianor) based on third-generation DNA sequencing and Hi-C analysis

Lu et al describe the construction of a chromosomal-level reference genome assembly for Papilio bianor and set this in the context of other butterfly genome assemblies. This manuscript was a pleasure to review. The genome assembly project has been carefully conducted and a good level of supporting data is included. The manuscript requires some editing for language (almost all of my minor comments address language issues) but the intentions and the analyses that have been carried out are nevertheless clear and this assembly will be an important resource for the butterfly research community and beyond.

Major comments

Regarding repeat content, to what extent does your observation of such extremely high repeat content reflect your methods of ascertainment and methods used in other studies? For example, see Platt et al (2016) doi: 10.1093/gbe/evw009. How confident are you that this repeat expansion is genuine and not (partly or wholly) an artefact of ascertainment? Whilst this expansion sits neatly with the increased genome size, I would like to see some discussion of the limitations of making these broad comparisons between assemblies generated and analysed with different methodologies.

In a similar vein, it would also be interesting to see some discussion of how data from different wild-caught individuals was combined to generate the assembly draft. Especially in the context of such high heterozygosity this has the potential to cause difficulties in assembly and might inflate estimates of genome size. This was alluded to in 141-163, when criteria for merging contigs were set out. I think further discussion of whether this had an impact on the assembly process would be of general interest to the GigaScience readership.

It would be interesting to know why the authors selected P. xuthus for comparisons. Is there a particular biological justification? Table 1 suggests it may be considered the best-assembled of the other Papilio genomes. Perhaps include a statement in the text explaining why this pairwise comparison was selected?

Fig1b is an excellent overview but the detail of the heatmap is hard to see on this scale. Perhaps consider releasing per-chromosome plots as part of supplementary material.

In general, I dislike the statements of priority (e.g. in line 103- this is the 4th chromosomal-level reference genome) as these are prone to become quickly out of date and also are somewhat irrelevant.

Level of Interest

Please indicate how interesting you found the manuscript: Choose an item.

Quality of Written English

Please indicate the quality of language in the manuscript: Choose an item.

Declaration of Competing Interests

Please complete a declaration of competing interests, considering the following questions:

- Have you in the past five years received reimbursements, fees, funding, or salary from an organisation that may in any way gain or lose financially from the publication of this manuscript, either now or in the future?
- Do you hold any stocks or shares in an organisation that may in any way gain or lose financially from the publication of this manuscript, either now or in the future?
- Do you hold or are you currently applying for any patents relating to the content of the manuscript?
- Have you received reimbursements, fees, funding, or salary from an organization that holds or has applied for patents relating to the content of the manuscript?
- Do you have any other financial competing interests?
- Do you have any non-financial competing interests in relation to this paper?

If you can answer no to all of the above, write 'I declare that I have no competing interests' below. If your reply is yes to any, please give details below.

I declare that I have no competing interests

I agree to the open peer review policy of the journal. I understand that my name will be included on my report to the authors and, if the manuscript is accepted for publication, my named report including any attachments I upload will be posted on the website along with the authors' responses. I agree for my report to be made available under an Open Access Creative Commons CC-BY license (http://creativecommons.org/licenses/by/4.0/). I understand that any comments which I do not wish to be included in my named report can be included as confidential comments to the editors, which will not be published.

Choose an item.

To further support our reviewers, we have joined with Publons, where you can gain additional credit to further highlight your hard work (see: https://publons.com/journal/530/gigascience). On publication of this paper, your review will be automatically added to Publons, you can then choose whether or not to claim your Publons credit. I understand this statement.

Yes Choose an item.
